# Functional Gallic Acid-Based Dendrimers as Synthetic Nanotools to Remodel Amyloid-β-42 into Noncytotoxic Forms

**DOI:** 10.1021/acsami.1c17823

**Published:** 2021-12-07

**Authors:** Ana R. Araújo, Juan Correa, Vicente Dominguez-Arca, Rui L. Reis, Eduardo Fernandez-Megia, Ricardo A. Pires

**Affiliations:** † 3B’s Research Group, I3Bs − Research Institute on Biomaterials, Biodegradables and Biomimetics, University of Minho, Headquarters of the European Institute of Excellence on Tissue Engineering and Regenerative Medicine, AvePark, Parque de Ciência e Tecnologia, Zona Industrial da Gandra, 4805-017 Barco, Portugal; ‡ ICVS/3B’s − PT Government Associate Laboratory, 4805-017 Braga/Guimarães, Portugal; § Centro Singular de Investigación en Química Biolóxica e Materiais Moleculares (CIQUS), Departamento de Química Orgánica, Universidade de Santiago de Compostela, Jenaro de la Fuente s/n, 15782 Santiago de Compostela, Spain; ∥ Biophysics and Interfaces Group, Department of Applied Physics, Faculty of Physics, University of Santiago de Compostela, 15782 Santiago de Compostela, Spain

**Keywords:** dendrimers, gallic acid, amyloid-β, supramolecular assembly, Alzheimer’s disease

## Abstract

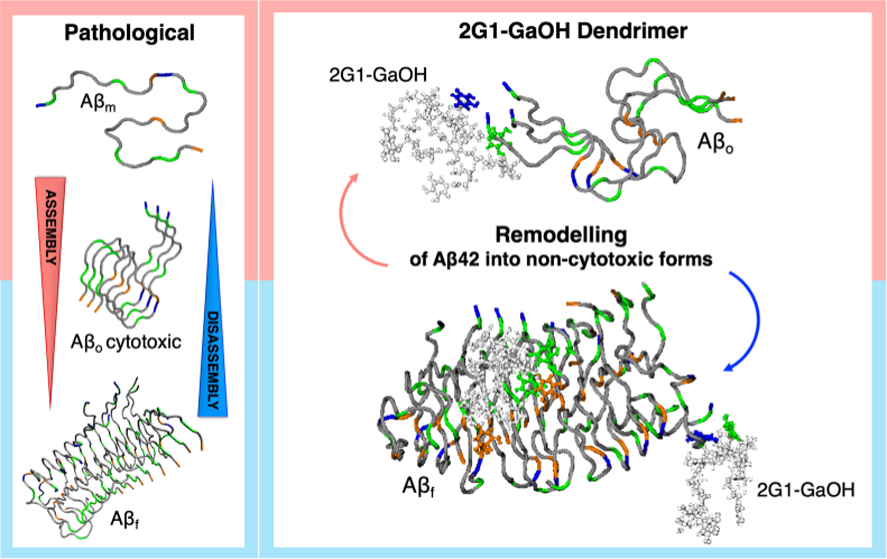

The self-assembly of amyloid-β (Aβ) generates cytotoxic oligomers linked to the onset and progression of Alzheimer’s disease (AD). As many fundamental molecular pathways that control Aβ aggregation are yet to be unraveled, an important strategy to control Aβ cytotoxicity is the development of bioactive synthetic nanotools capable of interacting with the heterogeneous ensemble of Aβ species and remodel them into noncytotoxic forms. Herein, the synthesis of nanosized, functional gallic acid (Ga)-based dendrimers with a precise number of Ga at their surface is described. It is shown that these Ga-terminated dendrimers interact by H-bonding with monomeric/oligomeric Aβ species at their Glu, Ala, and Asp residues, promoting their remodeling into noncytotoxic aggregates in a process controlled by the Ga units. The multivalent presentation of Ga on the dendrimer surface enhances their ability to interact with Aβ, inhibiting the primary and secondary nucleation of Aβ fibrillization and disrupting the Aβ preformed fibrils.

## 1 Introduction

Pathological protein/peptide aggregation is on the basis of several neurodegenerative processes, such as the ones leading to Alzheimer’s disease (AD).^1^ In AD, amyloid-β (Aβ) peptides of different lengths (between 38 and 43 amino acids) are at the onset and progression of the disease. These amyloidogenic Aβ species are highly prone to aggregate into a diverse set of supramolecular assemblies that range from short oligomers to long fibrils. Aβ peptides are produced by cleavage of the amyloid precursor protein (APP) promoted by the γ-secretase complex.^2^ The resulting peptides are reported to produce thermodynamically unstable oligomers (Aβ_o_) with a high propensity to self-assemble into stable β-sheet structures that present a fibril-like morphology (Aβ_f_). While high-molecular-weight fibrils have been shown to be relatively inactive, smaller aggregates (i.e., Aβ_o_) have emerged as potent cytotoxins with the ability to trigger neuronal cell death, both *in vitro* and *in vivo*.^3,4^ In fact, Aβ_o_ species can diffuse through the intracellular and pericellular spaces and interact with fundamental cellular motifs, triggering, for example, the hyperphosphorylation of Tau and hampering its ability to maintain the microtubules of neurons, which leads to their disassembly and neuronal cell death.^5^ The growth of Aβ_f_ in the cellular milieu occurs at the fibril surface, an autocatalytic process usually referred to as secondary nucleation, which is mediated by Aβ_o_ species.^6^ In addition to the size, Aβ_o_ differs from Aβ_f_ in the intermolecular arrangement of the β-strands (antiparallel for Aβ_o_ vs parallel β-sheet usually for Aβ_f_).^7^ These supramolecular assemblies are typically maintained through H-bonding between the backbones of nearby peptide monomers, as well as by π–π stacking between aromatic amino acid residues, making them key targets for the inhibition of the Aβ supramolecular assembly.^8^

**Figure 1:**
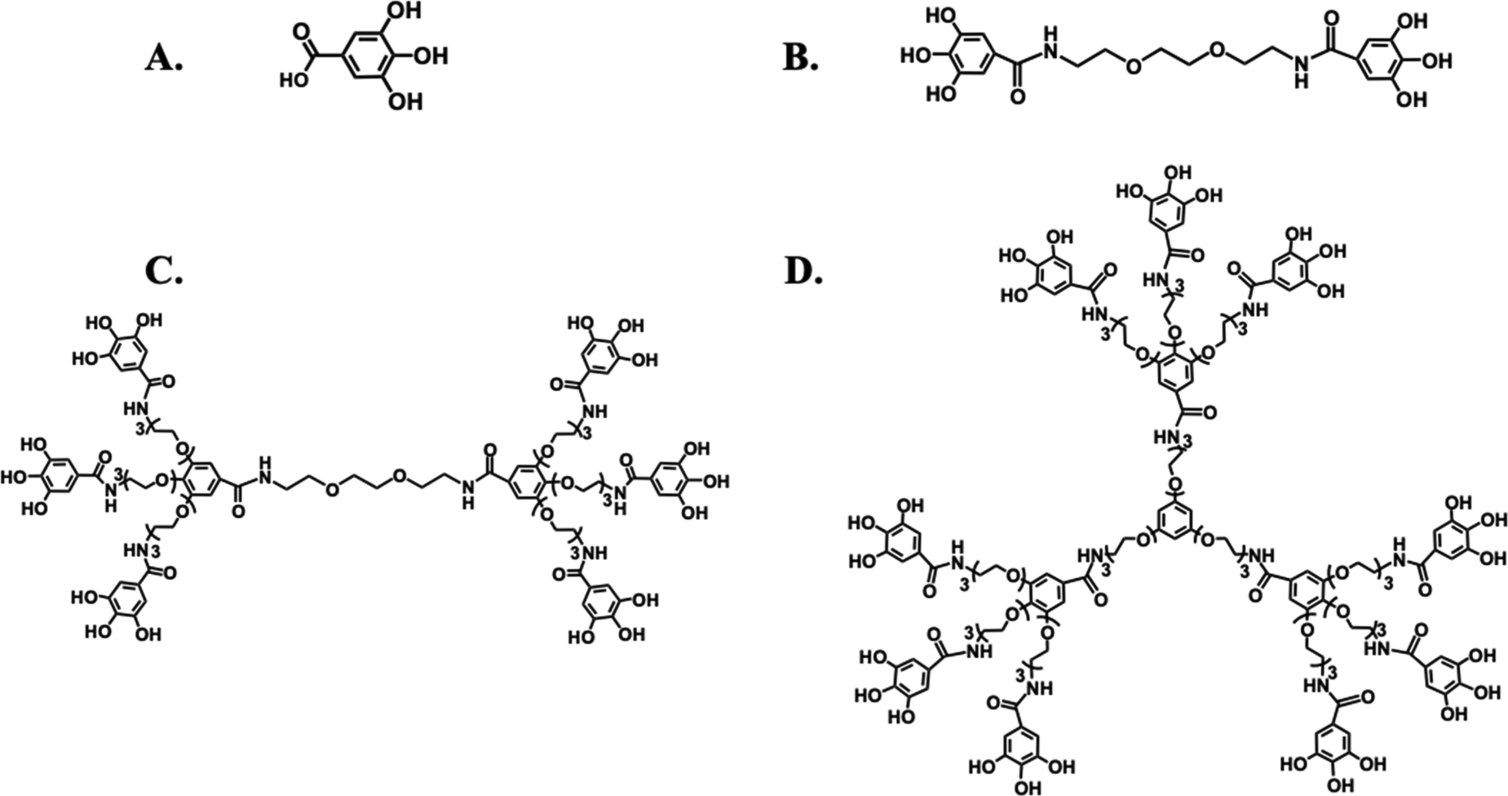
Chemical structure of the (A) Ga molecule and Ga-terminated dendrimers synthesized in the present work: (B) 2G0-GaOH, (C) 2G1-GaOH, and (D) 3G1-GaOH.

Natural polyphenols (e.g., epigallocatechin gallate,^9^ resveratrol,^10^ or vescalagin^11^) have been reported to remodel the supramolecular structure of amyloidogenic proteins/peptides.^12^ They usually interfere with the H-bonding and hydrophobic interactions (e.g., π–π stacking) between the protein/peptide chains that are responsible for the stabilization of β-sheets present in the Aβ supramolecular species. These perturbations usually lead to the formation of disordered Aβ assemblies of low toxicity.^9^ Importantly, this ability of polyphenols to interfere with the Aβ supramolecular assembly is reported to be mediated by gallic acid (Ga) and other phenolic units in their structures.^13^ Based on this bioactive potential, herein, we mimicked the structure of discrete polyphenols by designing nanosized dendrimers functionalized with an increasing number of Ga units (Figure 1A–D).

## 2 Materials and Methods

### 2.1 Materials

All chemicals were purchased from Sigma-Aldrich or Acros Organics and were used without further purification. All solvents were of HPLC grade, purchased from Scharlab and Fisher Chemical and used without further purification. DMSO and Et_3_N were dried under 4 Å molecular sieves. H_2_O of Milli-Q grade was obtained from a Millipore water purification system. 2[G1]-NH_2_·HCl and 3[G1]-NH_2_·HCl were prepared following previously reported procedures by our group.^14^

### 2.2 Synthesis of Ga-Terminated Dendrimers

The dendrimers used were synthesized divergently from gallic acid (Ga) repeating units. These dendrimers are composed of a hydrophilic triethylene glycol molecule carrying a Ga group at its terminals. The detailed synthesis of each Ga-terminated dendrimer can be found in the Supporting Information.

### 2.3 Dendrimer Characterization

We used column chromatography, NMR spectroscopy, infrared spectroscopy, mass spectrometry (MS), and gel permeation chromatography (GPC) to confirm synthesis of the Ga-terminated dendrimers, as shown in Figures S1–S24.

Therefore, in the column chromatography studies, an automated column chromatography was performed on a MPLC Teledyne ISCO CombiFlash RF 200 psi with RediSep Rf columns refilled with silica 40 mm (from VWR Chemicals) or neutral aluminum oxide 60-mesh (from Alfa Aesar). Samples were adsorbed onto silica 40 mm or neutral aluminum oxide 60-mesh into solid cartridges. The NMR spectra were recorded on a Varian Mercury 300 MHz spectrometer. Chemical shifts are reported in ppm (δ units) downfield from internal tetramethylsilane (CDCl_3_), or the residual solvent peak (CD_3_OD or DMSO-*d*_6_). Mestre Nova 9.0 software (Mestrelab Research) was used for spectral processing. The FT-IR spectra were recorded on a PerkinElmer Spectrum Two spectrophotometer equipped with a UATR accessory or Bruker Vertex 70v using KBr pellets. The mass spectra were recorder on a Bruker Microtof spectrometer coupled to a HPLC Agilent 1100 using atmospheric-pressure chemical ionization (APCI). Samples were injected via flow injection analysis (FIA) using a MeOH/aqueous solution of formic acid 0.1% 1:1, flow 0.2 mL/min. Finally, the GPC experiments were performed on an Agilent 1100 series separation module using a PSS SDV precolumn (5 μm, 8 × 50 mm^2^), a PSS SDV Linear S column (5 μm, 8 × 300 mm^2^), and a PSS SDV Lux Linear M column (5 μm, 8 × 300 mm^2^) equipped with an Agilent 1100 series refractive index and UV detectors. THF was used as the eluent at 1 mL/min. The samples were filtered through a 0.45 μm PTFE filter before injection.

### 2.4 Molecular Dynamics Simulations

All simulations were carried out during 80 ns. The systems were formed with Aβ_f_ fibril (2NAO pdb file^15^) composed of two fibril layers of three peptides each (1–42 residues, total of six peptide molecules) and a 1:1 ratio of each of the dendrimer:

- 2NAO solvated and neutralized;
- 2NAO + 6 Ga solvated and neutralized;
- 2NAO + 6 2G0-GaOH solvated and neutralized;
- 2NAO + 6 2G1-GaOH solvated and neutralized;
- 2NAO + 6 3G1-GaOH solvated and neutralized.

2G0-GaOH, 2G1-GaOH, and 3G1-GaOH were parametrized and their topologies were built by merging dendrons whose topology was built using ATB.^16^ The final system was minimized and simulated using GROMACS with the force field Gromos54a7.^17^

### 2.5 Peptide Preparation

Human amyloid-β peptide (1–42) was obtained by custom synthesis from GeneCust Europe (Luxembourg). Stock solutions of 0.45 mg were prepared by dissolving 10 mg of amyloid-β peptide (1–42, Aβ) in 2.217 mL of HFIP (Fluorochem Ltd., UK) according to the protocol described by Stine et al.^18^ In brief, Aβ was dissolved in HFIP (5 mg/mL) during 30 min at room temperature. HFIP was allowed to evaporate in open tubes overnight in the fume hood, and later during an additional 1 h under vacuum. Aβ aliquots were then stored at −20 °C and reconstituted immediately before use: (i) for the monomeric Aβ (Aβ_o_) studies, 20 μL of DMSO (sonicated for 10 min in an ultrasound bath) was added and immediately afterward, ice-cold water was added (followed by 15 s of vortex) to a final concentration of 100 μM; (ii) for the fibrillar Aβ (Aβ_f_) experiments, 20 μL of DMSO (sonicated for 10 min in an ultrasound bath) was added and immediately afterward, 10 mM HCl was added at room temperature, diluting to a final concentration of 100 μM of Aβ, followed by vortexing for 15 s and then incubated for 24 h at 37 °C.

### 2.6 Thioflavin-T (ThT) Assay

**Figure 2:**
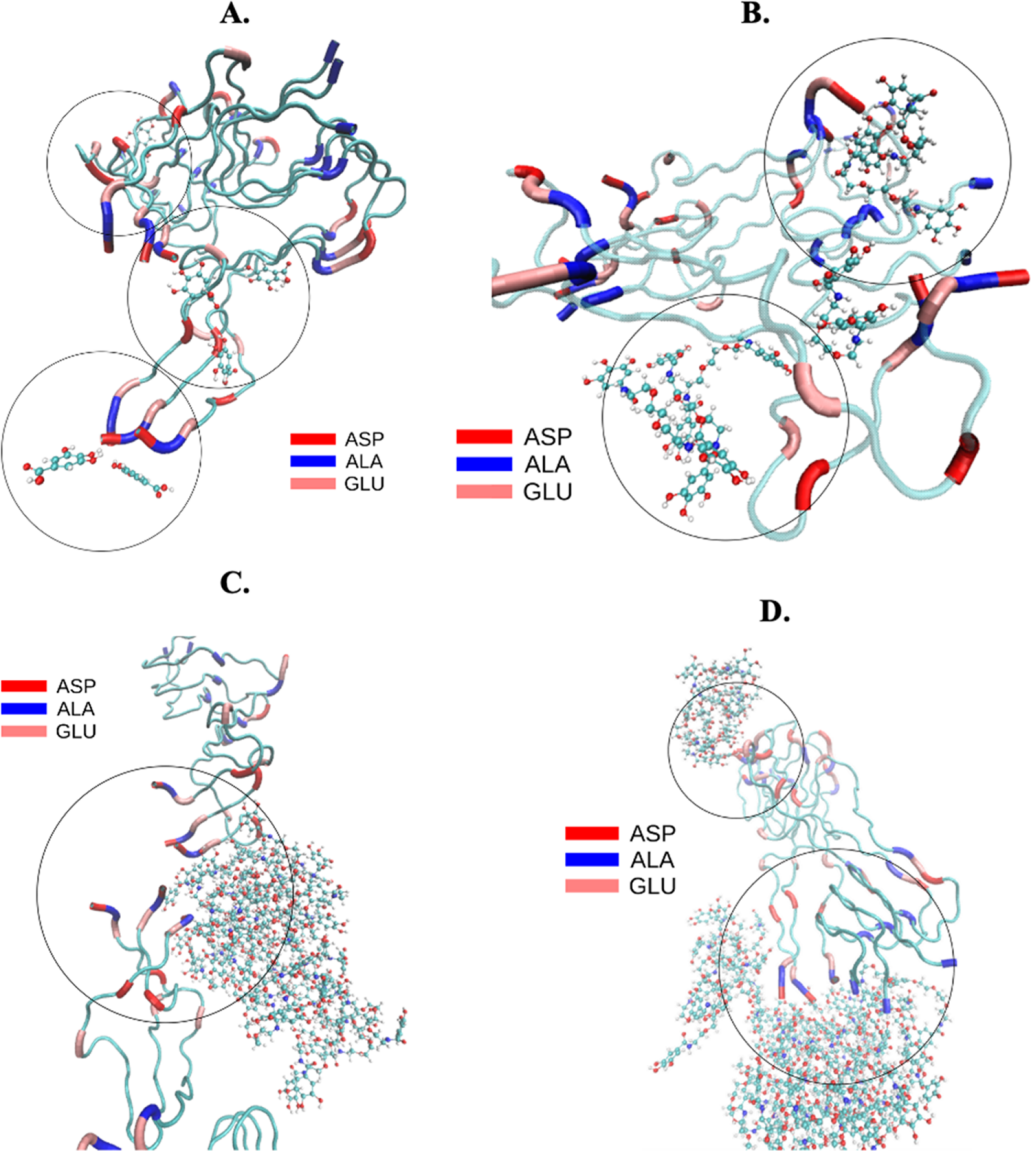
Optimized geometry from MD simulations of (A) Ga, (B) 2G0-GaOH, (C) 2G1-GaOH, and (D) 3G1-GaOH interacting with the full-length Aβ_f_ at its ASP (red), ALA (blue), and GLU (light pink) residues.

Fibril formation for Aβ_o_ was followed by the ThT assay (assembly—Figure 2A) during several days. Aβ peptide stock solution was prepared as described above. ThT fluorescence was monitored by mixing Aβ_o_ solution (final concentration of 25 μM on phosphate buffer 5 mM, with 0.1% sodium azide, pH 7.2) with ThT (final concentration of 40 μM) and different concentrations of Ga, 2G0-GaOH, 2G1-GaOH, and 3G1-GaOH, i.e., concentration ratios Aβ:dendrimers of 1:0.5; 1:1, and 1:2. ThT fluorescence was then recorded in a fluorescence spectrometer (Jasco, FP-8500, Japan) during 6 and 8 days using an excitation wavelength of 435 nm and an emission wavelength of 465 nm. Each experiment was repeated in triplicate. The experiments for the disassembly of Aβ fibrils, i.e., Aβ_f_, were performed for 5 days using the same experimental protocol.

### 2.7 Circular Dichroism (CD)

CD was performed using a 1 mm path length cell at 37 °C in a CD spectrometer (Jasco, J1500, Japan). Spectra were recorded in the range between 200 and 260 nm with a scan rate of 10 nm/min and a response time of 1 s. Three scans were accumulated for each spectrum. For all of the CD experiments, the Aβ_o_/Aβ_f_ concentration was 25 μM with or without each dendrimer for the different ratios: 1:0.5; 1:1, and 1:2.

### 2.8 Atomic Force Microscopy (AFM)

For the acquisition of AFM images, freshly cleaved mica was functionalized with a drop of (3-aminopropyl)triethoxysilane (APTES, 200 μL) during 30 min at room temperature. Afterward, the micas were rinsed with deionized water and dried under a nitrogen flux. Each sample, Aβ_f_ peptide (30 μM) in the presence and absence of dendrimers, was spotted onto the functionalized mica during 30 min, washed with water and dried under nitrogen.

AFM images were acquired using a JPK Nanowizard 3 (JPK, Germany) in air at room temperature under the AC mode. The scans were acquired at a 512 × 512 pixel resolution using ACTA-SS probes (*k* ∼ 37 N/m, AppNano), a drive frequency of ∼254 kHz, a setpoint of ∼0.5 V, and a scanning speed of 1.0 Hz.

### 2.9 Scanning Transmission Electron Microscopy (STEM)

For the acquisitions of the STEM images, Aβ_f_ peptide (30 μM) in the presence and absence of dendrimers, was spotted onto the TEM grids, Carbon Type-B 400M Cu (IESMAT, Spain), during 3 min, followed by the classic staining with UranyLess (10 μL each sample, for 2 min) (EMS, UK). All samples were washed with water and dried under nitrogen. STEM images were obtained using a high-resolution field emission scanning electron microscope (SEM—Auriga Compact, Zeiss, Germany). The scans were acquired at a resolution of 2048 × 1536 pixels, EHT = 3000 kV, WD = 2.9 mm, and magnification = 5000 K.

### 2.10 Western Blot (WB) Analysis

To assess the effect of dendrimers in the size of the Aβ species, produced in the presence of different Aβ:dendrimer ratios, i.e., 1:0.5; 1:1, and 1:2 (during 1 day and 5 days), we ran a 12% Bis–Tris Gel Invitrogen NuPAGE, with MES SDS Running Buffer gel (gel loading buffer without DTT), followed by its transference to nitrocellulose membranes using the iBlot 2 System. Next, the nitrocellulose membranes were blocked with 4% BSA for 1.5 h in TBS-T (TBS with 0.1% Tween 20%). After the blocking, the membranes were incubated (overnight at 4 °C) with the 6E10 antibody (Aβ 1–16—1:1000 dilution in 4% BSA in TBS-T). After washing three times in TBS-T, the membranes were incubated (during 1.5 h at RT) in IRDye 800CW goat anti-mouse IgG secondary antibody (1:10000 dilution in TBS-T). Finally, the WB lanes were detected using an Odyssey Fc Imaging System (LI-COR Inc., NE). The comparisons between bands in different lanes were quantified using Fiji software.

### 2.11 Cell Studies

Neuroblastoma SH-SY5Y cells were cultured at 37 °C in a humidified 95/5% air/CO_2_ atmosphere using Dulbecco’s modified Eagles medium F-12 (Gibco, UK) supplemented with 10% FBS (Gibco, UK) and 1% ATB (Gibco, UK) solution. The cell medium was replaced every 2 days and cells were subcultured once they reached 90% confluence. Cells were plated at a density of 25 000 cells per well on 96-well plates containing DMEM/F-12 media (for AlamarBlue assay) and plated at a density of 50 000 cells per well on 24-well plates containing DMEM/F-12 media (for the live/dead assay). A typical experiment included the culture of the neuroblastoma cell line (SH-SY5Y) during 24 h or 5 days in the absence or presence of dendrimers at different concentrations. Afterward, Aβ_o_/Aβ_f_ was added to the culture medium and, after an additional 24 h, the cells were evaluated for their metabolic activity. The dendrimers and Aβ_o_/Aβ_f_ peptide were reconstituted in DMSO (0.02%), diluted into DMEM/F-12 media, sterilized by UV, and immediately added to the cells.

### 2.12 AlamarBlue Assay

Aβ_o_/Aβ_f_ cytotoxicity was evaluated by measuring the cell metabolic activity 24 h after the addition of Aβ_o_/Aβ_f_ at a concentration of 25 μM using AlamarBlue (indicator dye BUF012B, Bio-Rad) according to the manufacturer’s instructions. The fluorescence intensity of each experimental condition was measured using an excitation wavelength of 530 nm and an emission wavelength of 590 nm with a Synergy HT microplate reader (BioTek Instruments). *p*-values were calculated using a two-tailed *t*-test. The results are presented as mean ± SEM of six independent experiments for each experimental condition.

### 2.13 Live/Dead Assay

Cell viability was also evaluated by the live/dead assay using calcein AM (Life Technologies) to stain live cells and propidium iodide (PI, Biotium, Inc.) to stain dead cells. Viable cells were stained in green and dead cells were stained in red. Briefly, cells were incubated for 20 min with both dyes and then observed under a fluorescence microscope (Axio Imager Z1m, Zeiss, Germany).

### 2.14 Quantification of the β-Sheet Content under Cell Culture Determined by the ThT Assay

Cells were cultured with Aβ_o_/Aβ_f_ and dendrimers during 24 h. Afterward, 1% thioflavin-T (ThT, in sterile D-PBS) was added to the wells. After 20 min, the ThT fluorescence intensity was measured using a fluorescence spectrometer (Jasco, FP-8500, Japan), using an excitation wavelength of 435 nm and an emission wavelength of 465 nm with a bandwidth of 10 nm. Each condition was repeated in triplicate and using data from two independent experiments.^19^

### 2.15 Protein Expression

For immunostaining, fluorescence images were collected after 1 and 5 days of cell culture. Samples were washed twice with PBS, fixed in 10% neutral buffered formalin for 30 min at 4 °C, permeabilized with 0.1% Triton X-100 in PBS for 5 min, and blocked with 3% BSA in PBS for 30 min at room temperature. To evaluate the accumulation of Aβ_o_, a primary antibody (oligomer polyclonal antibody, clone A11, rabbit, 1:200 dilution in 1% w/v BSA/PBS, Life Technologies) against Aβ oligomeric forms or a primary antibody against Aβ_f_ aggregates (biotin anti-β-amyloid, 1–16 antibody, mouse IgG1, 1:200 dilution in 1% w/v BSA/PBS, Biolegend) was employed, followed by the secondary antibody rabbit anti-mouse Alexafluor-488 (1:500 dilution in 1% w/v BSA/PBS, anti-mouse, Invitrogen). A phalloidin-TRITC conjugate was used (1:200 dilution in PBS for 30 min, Sigma) to assess the cytoskeleton organization. Nuclei were counterstained with 1 mg/mL of 4,6-diamidino-2-phenylindole (DAPI, Sigma) for 30 min. Samples were washed with PBS and placed in an imaging dish for confocal microscopy (Leica, TCS SP8).

### 2.16 Bio-AFM Experiments

The cell nanomechanical analysis was performed using a JPK Nanowizard 3 (JPK, Germany) under PBS at 37 °C. Force curves were acquired using sQube cantilever (with tips of borosilicate spheres of 5 μm diameter, CP-qp-CONT-BSG, sQube), presenting *k* ∼ 0.1 N/m. All cantilevers were calibrated before performing the analysis using the JPK noncontact method. For each experimental condition, at least 15 fixed cells were analyzed and it was acquired at least 10 curves per cell. All force curves were fitted using the Hertz model to obtain Young’s modulus.

For the cell height analysis, at least 10 cells per experimental condition were analyzed under the JPK QI Imaging Mode, using cantilevers qp-BioAC-CB1 (NanoSensors, Germany). To obtain cell height data, cellular cross sections were retrieved from the AFM height images. All of the presented data are averages of 10 cells with the corresponding standard deviations.

## 3 Results and Discussion

### 3.1 Synthesis of Ga-Terminated Dendrimers

For the preparation of dendrimers functionalized with Ga units on the periphery, we selected dendritic scaffolds of the gallic acid–triethylene glycol (GATG) family^14,20^ with terminal amine groups. GATG dendrimers are composed of Ga cores, responsible of the multivalency, and long triethylene glycol spacer arms intended to give flexibility to the macromolecular structure. Depending on the nature of the dendritic core, divalent or trivalent, GATG dendrimers are referred to as 2Gn or 3Gn (n is the dendrimer generation). Amide coupling of the 2G0 and 2G1 aminodendrimers (EDC, HOBt) with a Ga derivative with hydroxyl groups protected as benzyl ethers, followed by deprotection (H_2_, Pd/C), afforded 2G0-GaOH (2 Ga units) and 2G1-GaOH (6 Ga units). We tested the synthesis of the 2G2-GaOH dendrimer (18 Ga units, not shown); however, its high hydrophobic character made it insoluble in water. To generate a dendrimer of higher Ga valency, the same strategy was applied to a 3G1 dendrimer, leading to 3G1-GaOH (9 Ga units). All of the functional Ga-based dendrimers were obtained in very good yields. Their chemical characterization is described with convincing evidence of a successful synthesis by ^1^H, ^13^C NMR, and IR spectroscopy. Furthermore, their purity and monodispersity were confirmed by gel permeation chromatography (Figures S1–S24).

### 3.2 Molecular Dynamics Simulations of Dendrimer–Aβ Interactions

The ability of 2G0-GaOH, 2G1-GaOH, and 3G1-GaOH was assessed to modulate the supramolecular assembly of Aβ and to promote the formation of noncytotoxic aggregates. We started by modeling the potential of the dendrimers to disassemble the full-length Aβ_f_ by coarse-grain all-atom molecular dynamics (MD) simulations (i.e., 2NAO PDB structure,^15^Figure S25). For this analysis, Aβ_o_ was not considered since their dynamic character largely limits the reproducibility of the data obtained during the relative short timeframes of the simulations.^21^ We then assessed the stability of Aβ_f_ in the presence and absence of dendrimers (Figures 2 and S26–S29 and Table S1).

All dendrimers exhibited strong and stable H-bonds with the Glu, Ala, and Asp residues of the Aβ_f_ structure. The placement of dendrimers in close proximity to the region 3–8 (i.e., Glu–Phe–Arg–His–Asp–Ser) increases the spacing between Aβ strands (Figure 2 Asp (red), Ala (orange), and Glu (light pink)), compromising the formation of stable β-sheets and other secondary conformations maintained by the H-bonding network.^22^ While Ga and the dendrimer with lower number of Ga units (i.e., 2G0-GaOH) did not alter significantly, the solvent-accessible surface area (SASA, Table S1) of Aβ_f_, the dendrimers presenting six Ga units (i.e., 2G1-GaOH) significantly increased the Aβ SASA value. Further increment in the number of Ga units, i.e., 3G1-GaOH (nine units), reduced the SASA probably due to its high hydrophobic character, that induces a higher interaction between the dendrimers themselves at the expense of Aβ–dendrimer interactions.

The number of H-bonds between Aβ and different dendrimers proportionally increase with the number of Ga units in the dendritic structure. Despite the constraints derived from the increased hydrophobic character with multivalency, our results clearly show that the Ga units are the chemical moieties that mediate the interaction between the dendrimers and Aβ. Importantly, in general, MD simulations indicate that the dendrimers are able to interact with Aβ_f_, 2G1-GaOH being the most effective modulator of its supramolecular structure under physiological conditions.

### 3.3 Interference of Dendrimers in the Aβ Supramolecular Assembly

**Figure 3:**
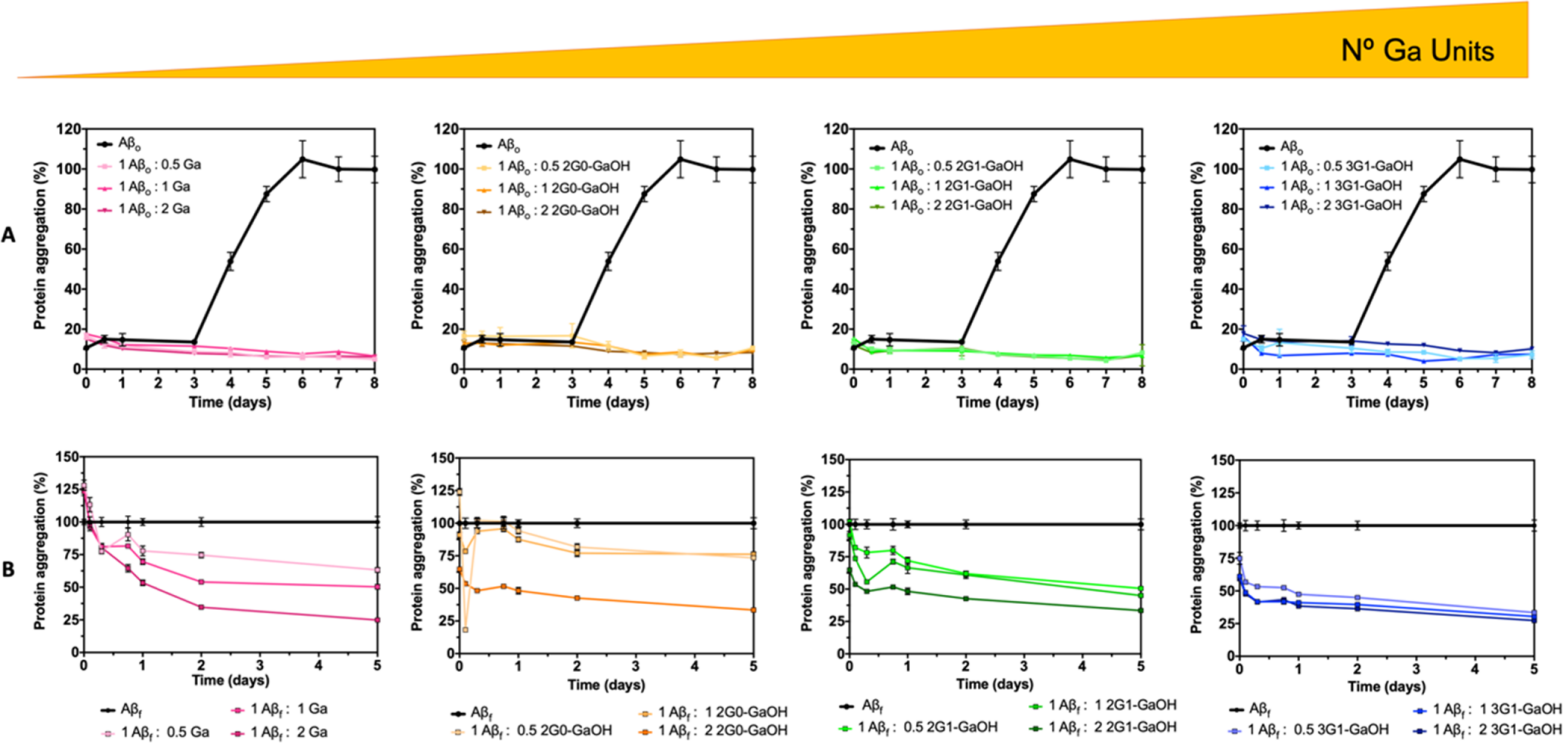
(A) Inhibition of the Aβ_o_ aggregation kinetics and (B) disassembling of Aβ_f_ fibrils evaluated using the ThT assay. Ga, 2G0-GaOH, 2G1-GaOH, and 3G1-GaOH, inhibited the Aβ fibril elongation and promote the rupture of Aβ_f_ fibrils at different molar ratios: 1:0.5, 1:1, and 1:2. Dendrimers were mixed with Aβ_o_ in the lag-phase of the Aβ fibril formation or with Aβ_f_ in the plateau phase, and fluorescence was measured over 8 days (Aβ_o_) or 5 days (Aβ_f_). All experiments were performed at room temperature.

The ability of the dendrimers to interfere with the assembly of Aβ *in vitro* was then assessed using the thioflavin-T (ThT) fluorescence assay. Fluorescence spectra of the dendrimers revealed no major interference with the ThT emission spectrum (Figures S30–S33). We analyzed the dendrimers under both assembly (Aβ_o_) and disassembly (Aβ_f_) conditions (Figure 3) to screen their potential to block the Aβ supramolecular assembly, as well as to disrupt the preassembled Aβ structures. The supramolecular assembly of Aβ_o_ over time presents the typical sigmoidal curve (characteristic of secondary nucleation and fibril elongation),^23^ which is abolished in the presence of all dendrimers at any of the tested concentrations. After the exponential growth phase of Aβ_o_ (at day 3–4), the fluorescence reaches a plateau (at day 7). When the inhibition of assembly is analyzed on a per Ga unit (Aβ:Ga unit, 1:1 Figure S34A), we observe the same tendency for the 2G1-GaOH, presenting a lower ThT fluorescence, which is in agreement with the results presented in Figure 3. While our results are compatible with this conclusion, it is important to note that ThT binding assays are not always reliable. ThT does not recognize all types of Aβ supramolecular assemblies, which make it necessary that the analysis of the interaction is performed using other techniques, such as CD and/or WB. Nonetheless, our initial results are consistent with a direct relationship between multivalency and activity. Indeed, the rational design of highly selective multivalent nanosystems has several advantages,^24^ including a reduction of the concentration needed to exert a specific bioactivity. Also, multivalency drives the targeted activity to the area of interest by increasing the local concentration of bioactive moieties (Ga units in our case). The impact of the dendrimers in the disassembly pathway (Aβ_f_, Figures 3B and S34B) is also dependent on the number of Ga units per dendrimer and their concentration, a result consistent with the disruption of the interstrand H-bonding of the β-sheets. These interactions lead to a new thermodynamic equilibrium, i.e., formation of noncytotoxic aggregates that are not recognized by ThT. As previously referred, it is known that ThT does not bind to β-sheet rich globular proteins due to their highly twisted β-sheets with a lower content of β-strands.^25^

**Figure 4:**
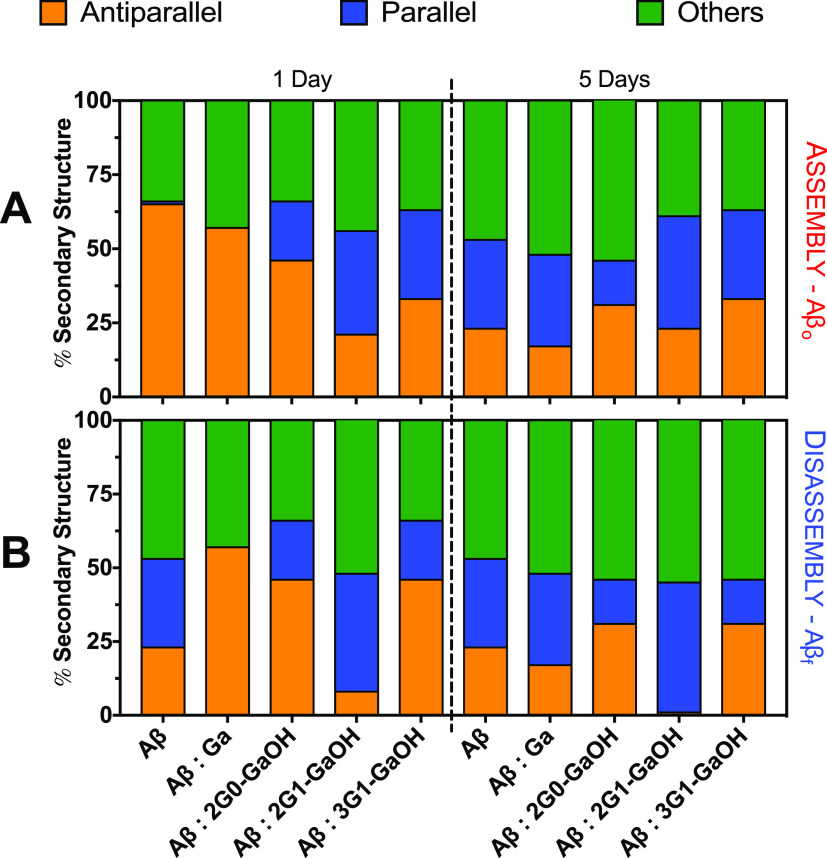
Loss of antiparallel β-sheets for the (A) aggregation pathway (Aβ_o_) and (B) disaggregation of (Aβ_f_) following CD during 1 and 5 days. All experiments were conducted using a Aβ:dendrimers ratio of 1:1, [Aβ] = 25 μM, and under constant agitation at 37 °C. Error bars = SD; *n* = 3. CD data were fitted using BeStSel—RMSD: 1.0283; NRMSD: 0.04966.

We then used circular dichroism (CD) spectroscopy to evaluate the type of β-sheets present in the Aβ supramolecular species generated upon interaction with the dendrimers. CD showed that all dendrimers perturbed the Aβ secondary structure (Figures S35–S42), probably leading to the formation of noncytotoxic oligomers. CD spectra were fitted using the BeStSel method^26^ for secondary structure estimation (Figure 4). After 1 day of incubation, a decrease in the antiparallel β-sheets for all Aβ_o_:dendrimer ratios was already visible, which were further reduced over 5 days of incubation. Remarkably, in the disassembly Aβ_f_:dendrimer experiments, 2G1-GaOH was able to abolish the antiparallel β-sheet content to undetectable levels after 5 days of incubation. When the activity was analyzed on a per Ga unit (Figure S43), 2G1-GaOH and 3G1-GaOH, presenting higher number of Ga units per dendrimer, revealed to be more efficient than Ga itself in reducing the antiparallel conformations associated with the Aβ_o_ cytotoxic forms, clearly showing that the multivalent presentation of Ga units enhances the ability of the dendrimers to interact with Aβ_o_. These results are in accordance with the ThT data, indicating a clear relationship between the concentration and multivalency of the dendrimers and their ability to interfere with the assembly of Aβ. These findings support the superior capacity of 2G1-GaOH to inhibit the catalytic mechanism that combines the growth of insoluble amyloid fibrils and the generation of soluble oligomeric aggregates and consequently secondary nucleation.

**Figure 5:**
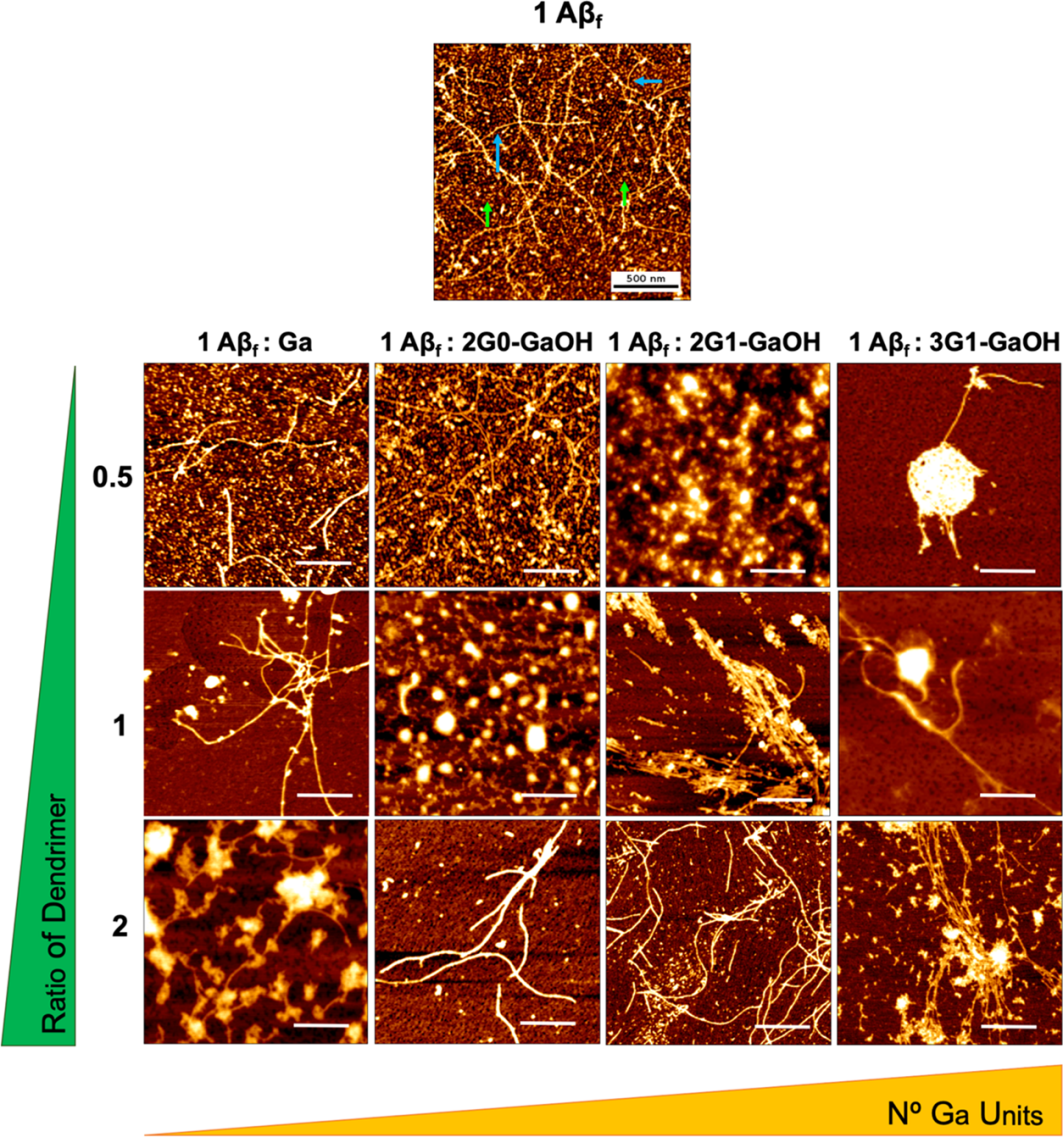
Representative AFM images of Aβ_f_. Each dendrimer was added into an Aβ_f_ solution (at Aβ:dendrimer concentration molar ratios of 1:0.5, 1:1, and 1:2) and left to incubate for 24 h under constant agitation. Both compounds directly altered Aβ_f_ presentation. Fibers marked with blue arrows and oligomers marked with green arrows in the Aβ_f_ control sample. Scale bars = 500 nm.

Next, we evaluated the morphological changes of the Aβ_f_ aggregates upon contact with the dendrimers by AFM (Figure 5) and STEM (Figure S44). As expected, a reduction in the number of small aggregates is observed, a morphological presentation compatible with cytotoxic oligomers or other small Aβ species. In addition, elongated fibrils are replaced by unstructured aggregates (condensed and less organized) and/or shorter fibers as a result of the interaction between Aβ_f_ and the dendrimers. When the interaction was studied on a per Ga unit basis by AFM, the dendrimers with higher number of Ga units revealed to be more effective than monovalent Ga to disrupt the Aβ_f_ assemblies (Figure S45). Overall, the dendrimers were able to remodel the Aβ aggregation state into species with a morphology compatible with a reduced cytotoxicity. This remodeling is in agreement with CD and ThT data and clearly supports the importance of a Ga multivalent presentation to increase the ability of the dendrimers to modulate the Aβ supramolecular assembly.

**Figure 6:**
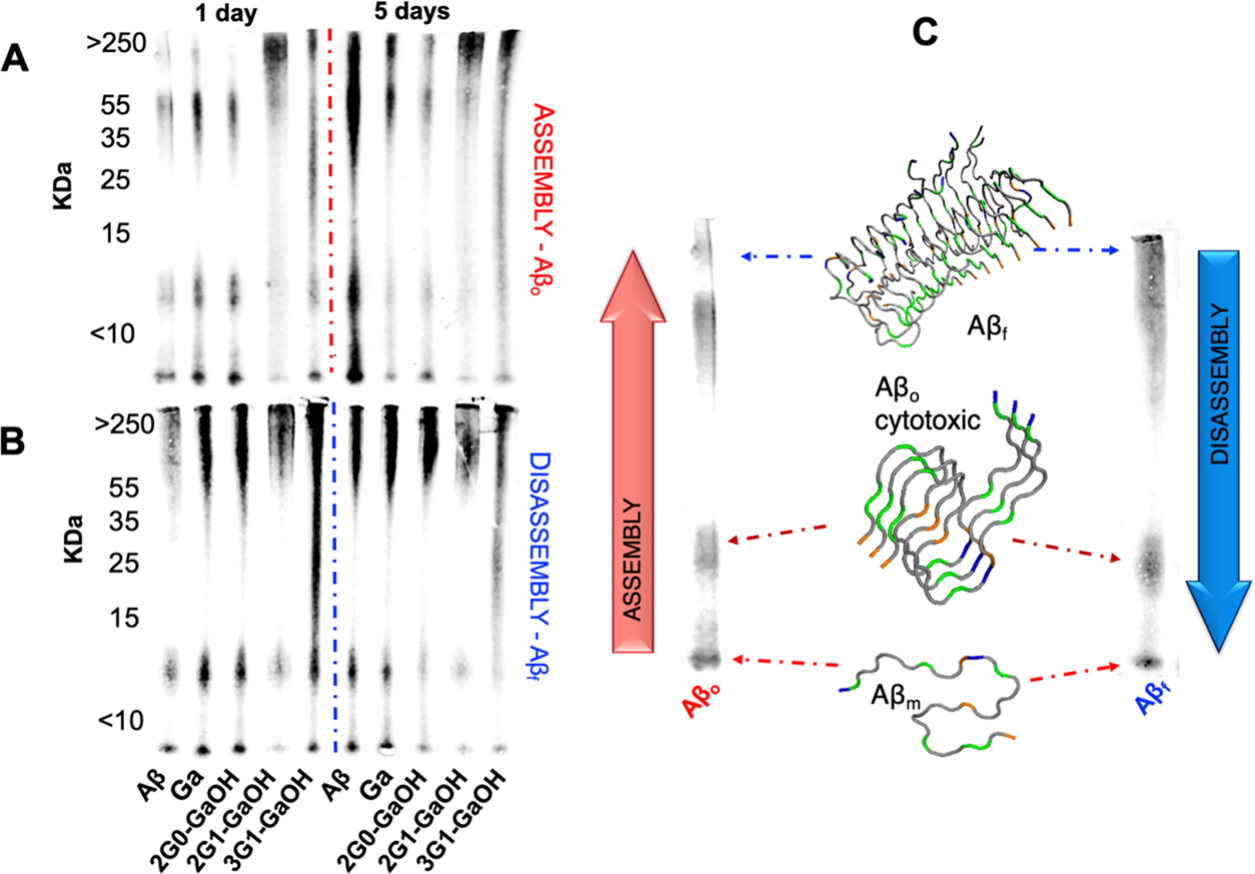
Relative densitometric bar graphs of (A) Aβ_o_ and (B) Aβ_f_ quantified by WB (using the antibody 6E10). Experiments performed using 25 μM of Aβ, 1:1 molar ratio of Aβ and dendrimers, at 37 °C in PBS, for 1 and 5 days. (C) Aβ peptide assembly and disassembly pathways.^4^

Western blot (WB, using the 6E10 antibody) allowed the quantification of the aggregates of different sizes, i.e., monomers, oligomers, and fibrils, produced during the assembly (Aβ_o_) and disassembly (Aβ_f_) processes (Figure 6). In both cases, 2G1-GaOH shows the highest reduction in the oligomeric and monomeric species after 1 and 5 days of incubation (Figures S46 and S47). The antibody 6E10 is specific for the 1–16 amino acids of the Aβ sequence, requiring an intact N-terminal epitope that encloses the amino acid sequence 3–8: Glu–Phe–Arg–His–Asp–Ser. From the MD simulations, we found that dendrimers exhibit strong and stable H-bonding interactions with Glu residues, so these results are compatible with the ability of 2G1-GaOH to interact directly with the Aβ N-terminal, blocking the epitope recognized by the 6E10 antibody.

### 3.4 Ability of Dendrimers to Modulate Aβ Cytotoxicity

After showing that the dendrimers (and particularly 2G1-GaOH) are able to modulate the supramolecular assembly of Aβ, both under assembly and disassembly conditions, we selected a neuroblastoma cell line (SH-SY5Y) to evaluate the impact of this activity under cell culture conditions.

**Figure 7:**
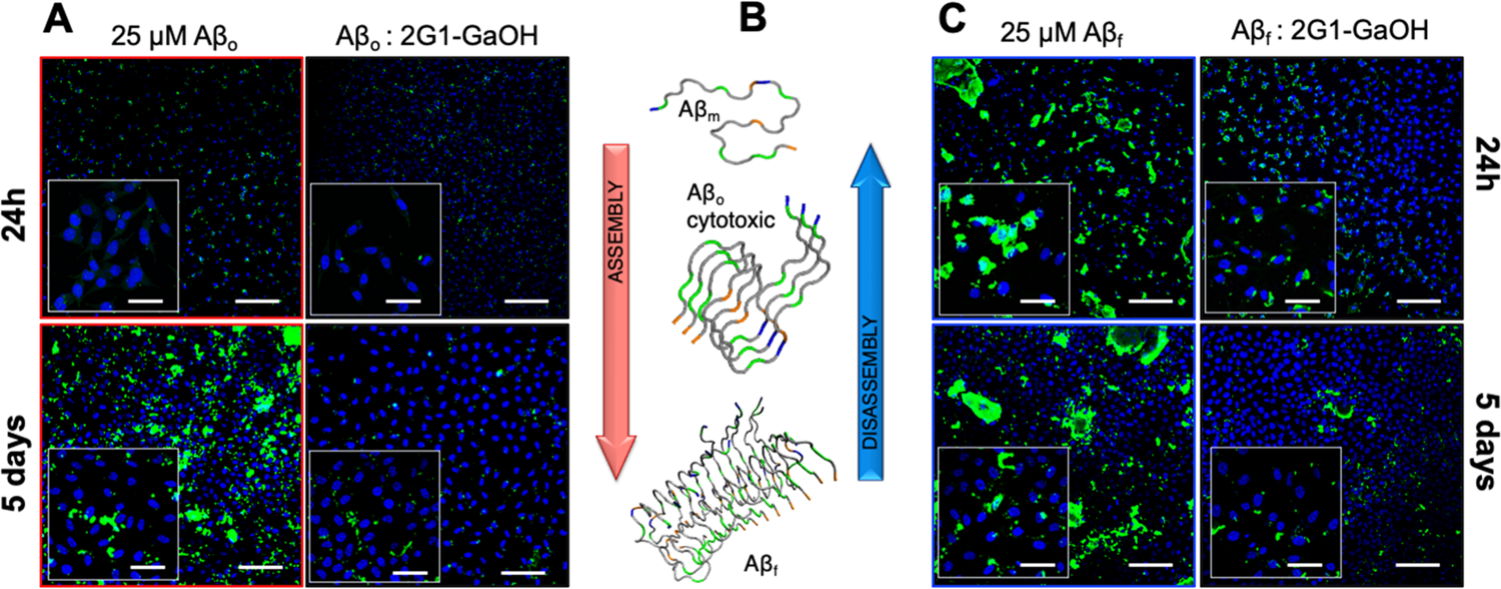
Immunofluorescence analysis of (A) Aβ_o_ (assembly) and (C) Aβ_f_ (disassembly) species in the SH-SY5Y cell culture (mAb 6E10) after incubation with 2G1-GaOH for 1 and 5 days (Aβ: green, cell nuclei: blue). Scale bar = 200 μm (insets = 50 μm). (B) Aβ peptide aggregation pathway.

All of the dendrimers were cytocompatible and able to rescue cells from the cytotoxicity of Aβ_f_ and Aβ_o_ (measured by AlamarBlue and live/dead assays observed under confocal microscopy, at 1 and 5 days of cell culture conditions, Figures S48–S57). Not surprisingly, when ThT was used to quantify the β-sheets present under cell culture conditions for both the assembly (Aβ_o_) and disassembly (Aβ_f_) experiments, ThT fluorescence reduced by ∼50–60% in the presence of the dendrimers (Figures S52–S54). Next, we used the 6E10 (stains Aβ_f_, Figures 7, S55, and S56) and A11 (stains Aβ_o_, Figures S57 and S58) antibodies to assess the presence of Aβ species in the pericellular space. We noticed that Aβ tends to aggregate (Aβ_f_ stained by 6E10) over the cell membrane, while the smaller species (Aβ_o_ stained by A11) are internalized by the cells. For both pathways, the 2G1-GaOH dendrimer reduced the amount of Aβ supramolecular assemblies in the cellular environment (Figure 7A,C).

**Figure 8:**
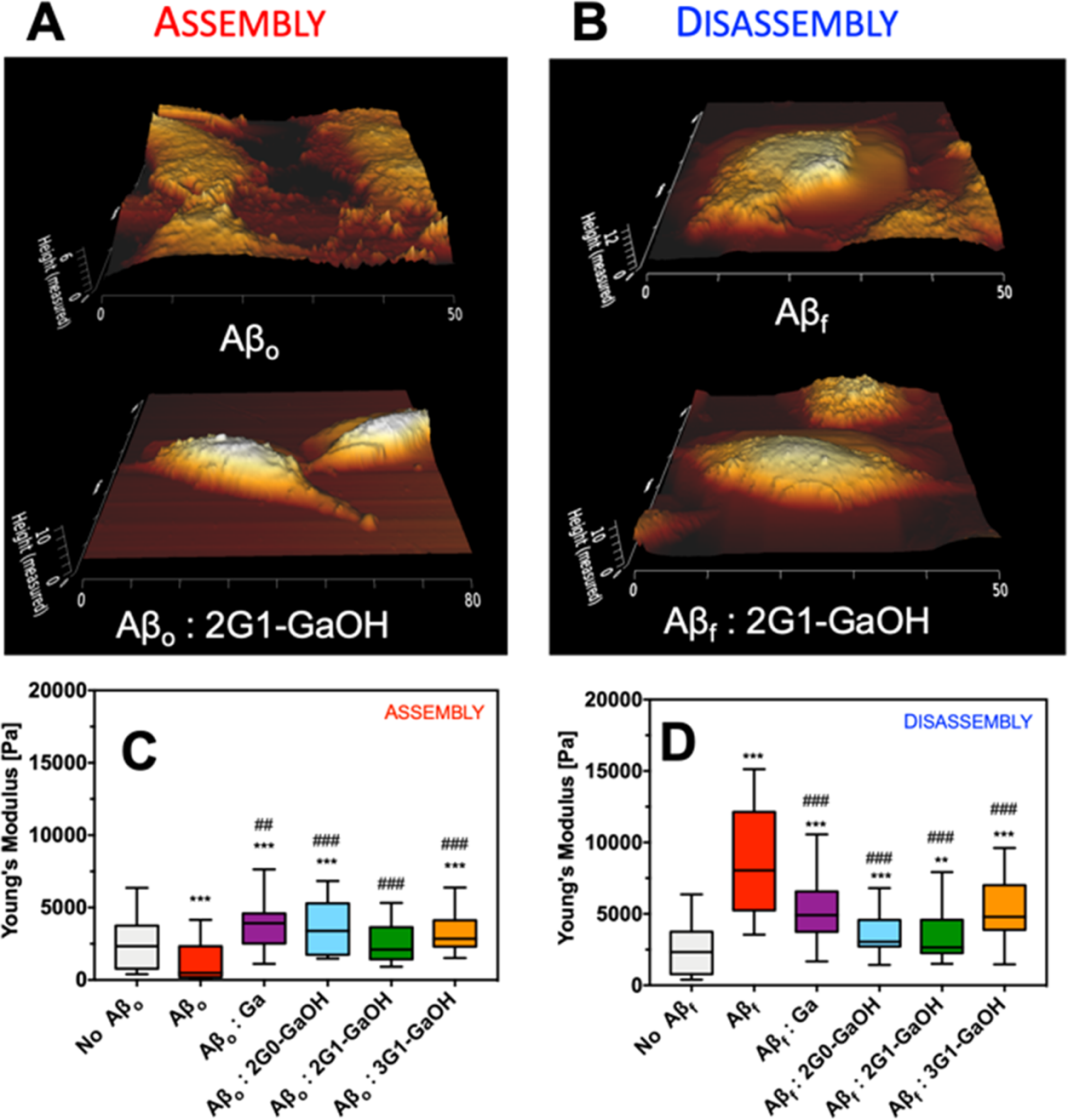
Representative Bio-AFM topographic images of SH-SY5Y cells ((A) assembly and (B) disassembly) cultured in the presence of Aβ and a 1:1 molar ratio of 2G1-GaOH. ((C) assembly and (D) disassembly) Young’s modulus of SH-SY5Y cells as a function of the presence of Aβ and dendrimers (molar ratio 1:1). Experiments were recorded after 24 h of incubation; Aβ concentration was set at 25 μM. Error bars = SD, ***p < 0.001, and **p < 0.01 (all vs control of no Aβ); ^###^*p* < 0.001 and ^##^*p* < 0.01 (all vs control of Aβ).

Recent reports^27^ have shown that Aβ species interact with the phospholipid bilayer changing the lipid environment. It has been proposed that the transformation of the Aβ secondary structure results in hydrophobic segments being exposed, which increases their tendency to attach onto the membrane. This interaction with the cell membrane disturbs the cytoskeletal organization (i.e., assembly of actin filaments and microtubules) influencing the cell mechanical properties, which results in changes in cellular morphology and mechanics (e.g., cell height and Young’s modulus).^28^ Therefore, we used Bio-AFM to measure the nanomechanical properties of the cells in the presence and absence of Aβ and dendrimers (Figures 8, S59, and S60). Our results indicate that the presence of Aβ_f_ increases the stiffness of the cell body, while Aβ_o_ (being more toxic) decreases Young’s modulus and cell height probably via Tau hyperphosphorylation leading to the disassembly of microtubules. In all of the cases, the presence of dendrimers ameliorated the perturbations promoted by Aβ leading to Young’s modulus and cell height closer to the control experiment, i.e., without the addition of Aβ and/or dendrimer.

Our results confirm that the bioactivity of the dendrimers directly relates to the number of Ga units. This was clear comparing the results obtained for 2G0-GaOH and 2G1-GaOH, where higher multivalency led to increased bioactivity. The lower performance of 3G1-GaOH (the highest multivalency dendrimer analyzed) is explained by its higher hydrophobic character that limits its solubility in water, reducing the dendrimer–Aβ interactions. Importantly, the increase of Ga units at the surface of the water-soluble dendrimers increases the local concentration of Ga units in their vicinity, making them more effective in modulating the supramolecular aggregation of Aβ, as observed for 2G1-GaOH. Importantly, in the presence of 2G1-GaOH, the kinetics of assembly/disassembly of the oligomers seems to be affected, and the formation of Aβ oligomers is no longer catalyzed by the preexisting Aβ fibrils. Our results are consistent with the inhibition of the self-replication of Aβ aggregates and of the generation of toxic oligomers. Moreover, increased cellular oxidative stress is usually considered a consequence of Aβ toxicity.^29^ Importantly, polyphenols (e.g., tannic acid, TA)^30^ are known to reduce the concentration of reactive oxygen species present in the (peri)cellular milieu. The proposed dendrimers (e.g., 2G1-GaOH), by reducing the number of cytotoxic aggregates, indirectly contribute to the maintenance of the cellular oxidative stress within the healthy physiological range. Finally, the higher hydrophobic character of the dendrimers might be an advantage to cross the blood–brain barrier when compared with natural polyphenols, such as TA, that are known to present a low BBB permeability, to be metabolized to lower-molecular-weight hydrolysable tannins and readily metabolized by the body through sequential enzymatic activity.^31^

## 4 Conclusions

We developed a strategy based on Ga-terminated dendrimers to inhibit the primary and secondary nucleation of Aβ fibrillization, as well as to disrupt the Aβ preformed fibrils. As in the case of natural polyphenols, the activity of the dendrimers was found to be proportional to the number of Ga units. We also prove that the multivalent presentation of these chemical motifs increases their capacity to remodel the Aβ secondary structure. Since increasing the number of Ga units at the surface of the dendrimer also increases its hydrophobic character and reduces its solubility in water, 2G1-GaOH (6 Ga units) emerges with a correct balance between solubility and bioactivity. Indeed, 2G1-GaOH maximizes the interaction with the Glu, Ala, and Asp amino acid residues (as shown by MD simulations), while reducing the Aβ cytotoxicity *in vitro*. The strategy herein presented can be extended to the development of dendrimers with alternative structural cores and other multivalent scaffolds aimed at abolishing the toxicity of Aβ assemblies in the context of AD. Also, we believe that these dendrimers represent relevant synthetic nanotools to study the assembly mechanisms of different Aβ peptide sequences, as well as to find structure–activity relationships of the Aβ cytotoxic oligomeric populations that have been linked to AD. Finally, our results set a strong background for testing the ability of the proposed dendrimers to cross the blood–brain barrier and reach the affected region of the brain, as well as to evaluate them under an *in vivo* scenario.

## Supporting Information Available

Synthesis and characterization of dendrimers, studies of interference of dendrimers in the Aβ supramolecular assembly, and cell studies (PDF)
