# Supplementary material for: Functional Gallic Acid-Based Dendrimers as Synthetic Nanotools to Remodel Amyloid-β-42 into Noncytotoxic Forms
Source: ACS Appl Mater Interfaces. 2021 Dec 7;13(50):59673–82. doi: 10.1021/acsami.1c17823 (PMC8704170; doi:10.1021/acsami.1c17823)
Supplement: Supplementary file 1 — am1c17823_si_001.pdf [file am1c17823_si_001.pdf]

SUPPLEMENTARY INFORMATION

Functional gallic acid-based dendrimers as synthetic nanotools to remodel amyloid-beta 42 into non-cytotoxic forms

Ana R. Araújo[1, 2*], Juan Correa[3], Vicente Dominguez-Arca[4], Rui L. Reis[1, 2], Eduardo Fernandez-Megia[3*], Ricardo A. Pires[1, 2*]

[1]3B's Research Group, I3Bs – Research Institute on Biomaterials, Biodegradables and Biomimetics, University of Minho, Headquarters of the European Institute of Excellence on Tissue Engineering and Regenerative Medicine, AvePark, Parque de Ciência e Tecnologia, Zona Industrial da Gandra, 4805-017 Barco, Portugal

[2]ICVS/3B's–PT Government Associate Laboratory, Braga/Guimarães, Portugal

[3]Centro Singular de Investigación en Química Biolóxica e Materiais Moleculares (CIQUS), Departamento de Química Orgánica, Universidade de Santiago de Compostela, Jenaro de la Fuente s/n, 15782 Santiago de Compostela, Spain

[4]Biophysics and Interfaces Group, Department of Applied Physics, Faculty of Physics, University of Santiago de Compostela, 15782 Santiago de Compostela, Spain

* E-mail: rpires@i3bs.uminho.pt; ef.megia@usc.es; anarita.araujo@i3bs.uminho.pt

Table of Contents

1 Synthesis and characterization studies *3*

Synthesis of Ga-terminated dendrimers **3**

Synthesis of Benzyl 3,4,5-tris(benzyloxy)benzoate 3
Synthesis of 3,4,5-Tris(benzyloxy)benzoic acid 3
Synthesis of 2[G0]-GaOBn 3
Synthesis of 2[G0]-GaOH 4
Synthesis of 2[G1]-GaOBn 4
Synthesis of 2[G1]-GaOH 4
Synthesis of 3[G1]-GaOBn 5
Synthesis of 3[G1]-GaOH 5

Characterization of Ga-terminated dendrimers **6**

Interference of the dendrimers in the Aβ supramolecular assembly **28**

2 Cell studies *49*

Ability of the dendrimers to modulate the Aβ cytotoxicity **49**

1 SYNTHESIS AND CHARACTERIZATION STUDIES

Synthesis of Ga-terminated dendrimers

Synthesis of Benzyl 3,4,5-tris(benzyloxy)benzoate: K_2CO_3 (12.04 g, 87.15 mmol), benzyl bromide (14.91 g, 87.15 mmol) and NaI (2.61 g, 17.43 mmol) were added to a stirred solution of gallic acid (3.0 g, 17.43 mmol) in MeOH (180 mL). The reaction was refluxed for 16 h, cooled down to rt and then, H_2O (30 mL) was added. After evaporation, the mixture was diluted with H_2O (50 mL) and extracted with EtOAc (3 x 20 mL). The combined organic layers were dried and concentrated to give a crude product that was recrystallized from EtOH to give benzyl 3,4,5-tris(benzyloxy)benzoate as a white solid (8.19 g, 88%). ^1H NMR (300 MHz, DMSO-*d6*) δ: 7.63-7.17 (m, 22H), 5.33 (s, 2H), 5.17 (s, 4H), 5.06 (s, 2H). ^{13}C NMR (75 MHz, DMSO-*d6*) δ: 165.3, 152.3, 141.7, 137.5, 136.9, 136.4, 128.7, 128.6, 128.4, 128.2, 128.1, 127.9, 127.8, 124.8, 108.4, 74.3, 70.4, 66.2. HRMS (APCI-FIA-TOF, positive mode, *m/z*): 531.2166. Calculated for $[M+H]^+$, $C_{35}H_{31}O_5$: 531.2171. IR (neat, ATR) ν_{max}: 3091, 3033, 1713, 1590, 1498, 1109 cm^{-1}.

Synthesis of 3,4,5-Tris(benzyloxy)benzoic acid: A stirred solution of benzyl 3,4,5-tris(benzyloxy)benzoate (5.69 g, 10.7 mmol) in a 3M KOH solution in MeOH (285 mL) was refluxed overnight. The mixture was cooled down to rt and acidified (pH= 3-4) by addition of 3M HCl. The resulting precipitate was filtered, dissolved in EtOAc (80 mL) and washed with H_2O (40 mL). The aqueous phase was extracted with CH_2Cl_2 (3 x 30 mL). The combined organic phase was dried and concentrated. The crude product was purified by automated MPLC (gradient from hexane to EtOAc, silica, 40 min) to afford 3,4,5-tris(benzyloxy)benzoic acid as a white solid (4.20 g, 89%). ^1H NMR (300 MHz, DMSO-*d6)* δ: 7.53-7.22 (m, 17H), 5.18 (s, 4H), 5.03 (s, 2H). ^{13}C NMR (75 MHz, DMSO-*d6)* δ: 166.9, 152.0, 140.9, 136.9, 128.4, 128.2, 128.1, 127.9, 127.6, 126.2, 108.2, 74.2, 70.2. HRMS (APCI-FIA-TOF, positive mode, *m/z*): 441,1694. Calcd for $[M+H]^+$, $C_{28}H_{25}O_5$: 441.1702. IR (neat, ATR) ν_{max}: 3029, 1686, 1595, 1128 cm^{-1}.

Synthesis of 2[G0]-GaOBn: 3,4,5-Tris(benzyloxy)benzoic acid (334 mg, 0.76 mmol), HOBt (103 mg, 0.76 mmol) and EDC (145 mg, 0.76 mmol) were added to a solution of 2,2'-(ethylenedioxy)bis(ethylamine) (45 mg, 0.30 mmol) in DMSO (6 mL). After 20 h of stirring at rt, the reaction mixture was distributed between EtOAc (30 mL) and brine (30 mL). The aqueous phase was extracted with EtOAc (3 x 30 mL). The combined organic phase was washed

with H_2O (30 mL), dried and evaporated under vacuum. The crude product was purified by automated MPLC (gradient from hexane to 100% EtOAc and then to 100% CH_2Cl_2, neutral alumina, 25 min) to afford 2[G0]-GaOBn (255 mg, 85%) as a white solid. ^1H NMR (300 MHz, $CDCl_3$) δ: 7.47-7.18 (m, 30H), 7.11 (s, 4H), 6.62-6.53 (m, 2H), 5.08 (s, 8H), 5.07 (s, 4H), 3.69-3.50 (m, 12H). ^{13}C NMR (75 MHz, $CDCl_3$) δ: 167.2, 152.8, 141.4, 137.5, 136.8, 129.9, 128.6, 128.2, 128.1, 128.0, 127.6, 107.1, 75.1, 71.4, 70.2, 69.8, 39.8. IR (neat, ATR) ν_{max}: 3310, 3064, 3033, 1634, 1581, 1112 cm^{-1}.

Synthesis of 2[G0]-GaOH: Pd/C (16 mg, 10%) was added to a solution of 2[G0]-GaOBn (160 mg, 0.16 mmol) in a mixture of DMF-MeOH 3:1 (4 mL). The resulting mixture was stirred under H_2 (1 atm) for 20 h. The catalyst was removed by filtration through Celite. The filtrate was concentrated and then purified by short column chromatography (MeOH-CH_2Cl_2-H_2O 3:3:0.2, silica) to give 2[G0]-GaOH as a green solid (71 mg, 98%). ^1H NMR (300 MHz, CD_3OD) δ: 6.85 (s, 4H), 3.67-3.57 (m, 8H), 3.50 (t, J=5.5 Hz, 4H). IR (KBr) ν_{max}: 3319, 1636, 1592, 1338, 1033 cm^{-1}.

Synthesis of 2[G1]-GaOBn: 3,4,5-Tris(benzyloxy)benzoic acid (466 mg, 1.06 mmol), Et_3N (0.224 mL, 1.06 mmol), HOBt (143 mg, 1.06 mmol) and EDC (210 mg, 1.06 mmol) were added to a solution of 2[G1]-NH_2·HCl (205 mg, 0.14 mmol) in DMSO (8 mL). After 24 h of stirring at rt, the reaction mixture was distributed between EtOAc (50 mL) and brine (50 mL). The aqueous phase was extracted with EtOAc (3 x 50 mL). The combined organic phase was washed with 0.1M HCl (30 mL) and H_2O (50 mL), dried and evaporated under vacuum. The crude product was purified by automated MPLC (gradient from EtOAc to 25% MeOH, neutral alumina, 25 min) to afford 2[G1]-GaOBn (428 mg, 75%) as a pale yellow solid. ^1H NMR (300 MHz, $CDCl_3$) δ: 7.40-7.10 (m, 102H), 6.97 (s, 4H), 5.03-4.94 (m, 36H), 4.07 (t, J=3.5 Hz, 4H), 3.96 (t, J=3.7 Hz, 8H), 3.75-3.38 (m, 72H). ^{13}C NMR (75 MHz, $CDCl_3$) δ: 167.2, 167.1, 152.5, 152.1, 140.9, 137.4, 136.7, 129.8, 128.4, 128.1, 127.8, 127.7, 127.4, 106.9, 75.0, 72.2, 71.1, 70.5, 70.3, 70.1, 70.0, 69.8, 69.6, 69.5, 68.6, 40.7, 39.7. IR (neat, ATR) ν_{max}: 3314, 3065, 3032, 1634, 1580, 1100 cm^{-1}.

Synthesis of 2[G1]-GaOH: Pd/C (60 mg, 20%) was added to a solution of 2[G1]-GaOBn (300 mg, 0.075 mmol) in a mixture of EtOAc-MeOH 1:1 (8 mL). The resulting mixture was stirred under H_2 (1 atm) for 14 h. The catalyst was removed by filtration through Celite. The filtrate was concentrated and then purified by short column chromatography (MeOH-CH_2Cl_2-H_2O

3:3:0.2, silica) to give 2[G1]-GaOH as a green solid (133 mg, 82%). ^{1}H NMR (300 MHz, CD_3OD) δ: 7.10 (s, 4H), 6.84 (s, 12H), 4.15-4.03 (m, 12H), 3.82-3.43 (m, 72H). IR (KBr) ν_{max}: 3296, 1625, 1593, 1330, 1116, 1097 cm^{-1}.

Synthesis of 3[G1]-GaOBn: 3,4,5-Tris(benzyloxy)benzoic acid (268 mg, 0.61 mmol), Et_3N (0.128 mL, 0.61 mmol), HOBt (82 mg, 0.61 mmol) and EDC (120 mg, 0.61 mmol) were added to a solution of 3[G1]-$NH_2\cdot HCl$ (134 mg, 0.05 mmol) in DMSO (4.9 mL). After 36 h of stirring at rt, the reaction mixture was distributed between EtOAc (50 mL) and brine (50 mL). The aqueous phase was extracted with EtOAc (3 x 50 mL). The combined organic phase was washed with 0.1M HCl (30 mL) and H_2O (50 mL), dried and evaporated under vacuum. The crude product was purified by automated MPLC (gradient from EtOAc to 25% MeOH, neutral alumina, 25 min) to afford 3[G1]-GaOBn (413 mg, 84%) as a pale yellow solid. ^{1}H NMR (300 MHz, $CDCl_3$) δ: 7.37-7.08 (m, 153H), 6.89 (s, 6H), 5.91 (s, 3H), 4.93 (s, 54H), 4.04-3.81 (m, 24H), 3.67-3.33 (m, 120H). ^{13}C NMR (75 MHz, $CDCl_3$) δ: 167.2, 160.4, 152.6, 152.1, 140.9, 137.5, 136.7, 129.9, 129.8, 128.4, 128.1, 127.9, 127.5, 107.0, 106.8, 94.3, 75.1, 72.2, 71.2, 71.1, 70.5, 70.3, 70.1, 69.8, 69.5, 68.6, 67.3, 40.0. IR (neat, ATR) ν_{max}: 3319, 3069, 3034, 1635, 1581, 1108 cm^{-1}.

Synthesis of 3[G1]-GaOH: Pd/C (10 mg, 20%) was added to a solution of 3[G1]-GaOBn (49 mg, 0.008 mmol) in a mixture of DMF-MeOH 4:1 (3 mL). The resulting mixture was stirred under H_2 (1 atm) for 24 h. The catalyst was removed by filtration through Celite. The filtrate was concentrated and then purified by short column chromatography (MeOH-CH_2Cl_2-H_2O 3:3:0.2, silica) to give 3[G1]-GaOH as a green solid (26 mg, 90%). ^{1}H NMR (300 MHz, DMSO-d6) δ: 7.17 (s, 6H), 6.81 (s, 18H), 6.07 (s, 3H) 4.18-3.94 (m, 24H), 3.80-3.20 (m, 120H). IR (KBr) ν_{max}: 3334, 1629, 1597, 1336, 1109, 1097 cm^{-1}.

Characterization of Ga-terminated dendrimers

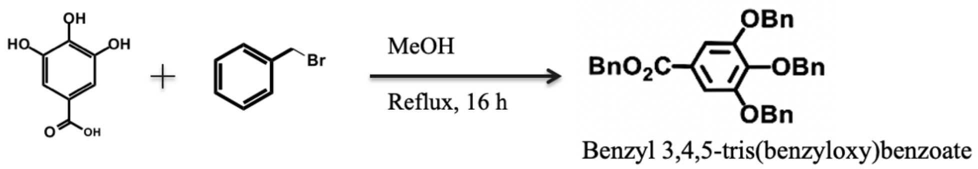

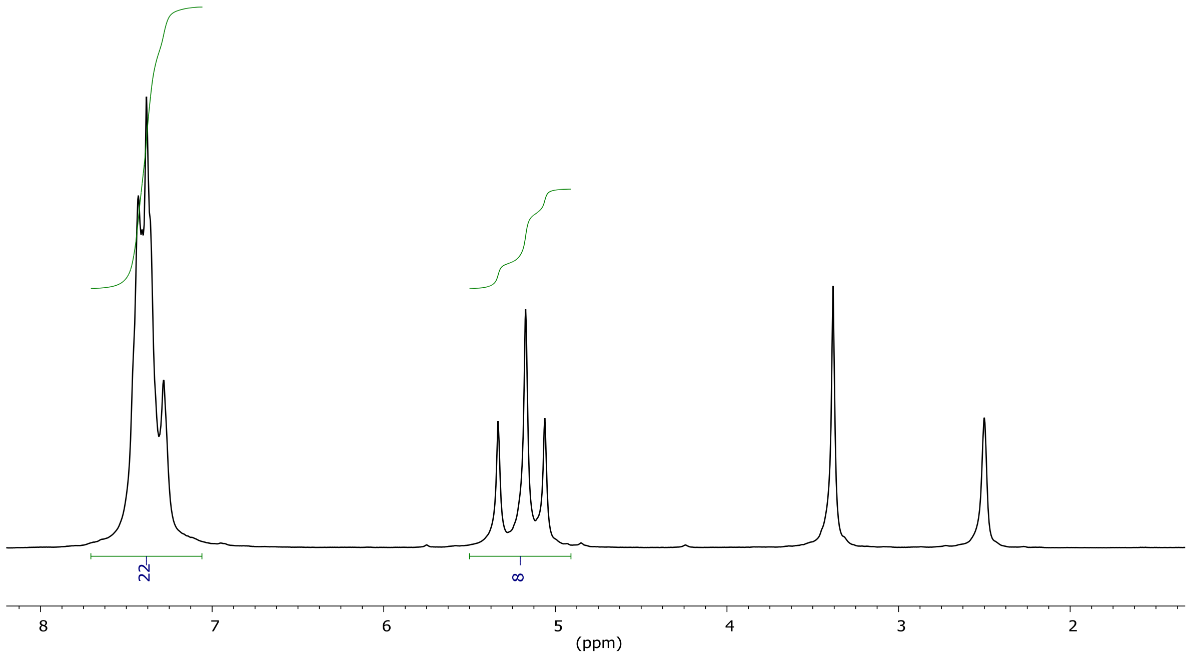

Figure S1. ^{1}H NMR spectrum (DMSO-d_6) of benzyl 3,4,5-tris(benzyloxy)benzoate**.**

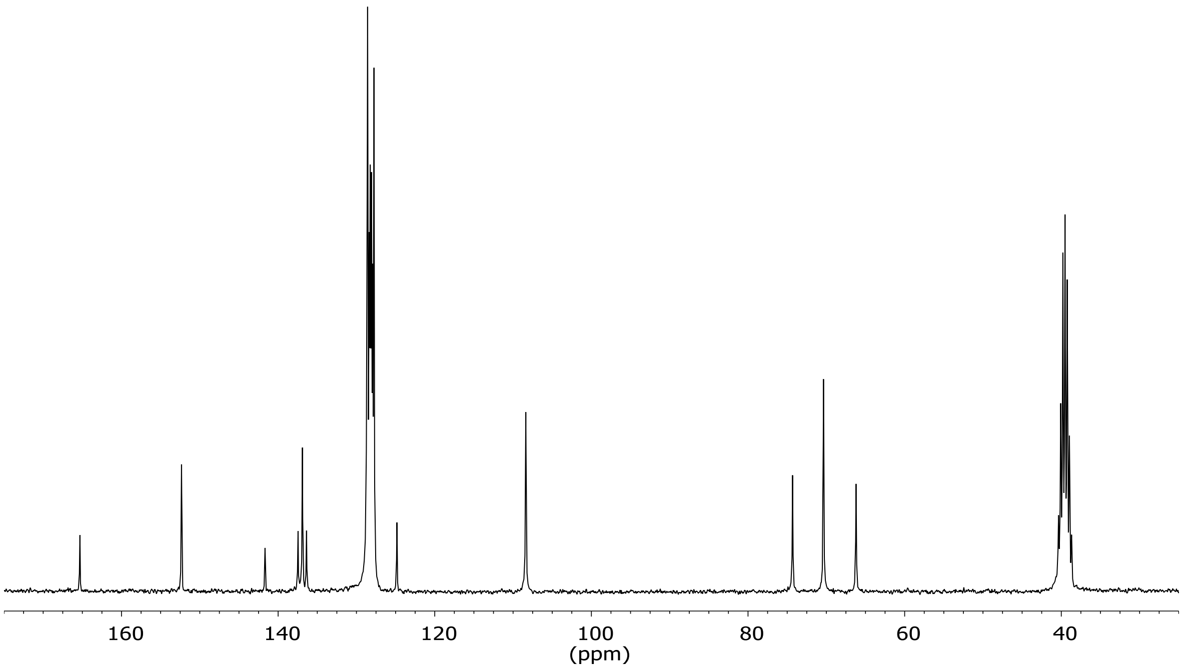

Figure S2. ^{13}C NMR spectrum (DMSO-d_6) of benzyl 3,4,5-tris(benzyloxy)benzoate.

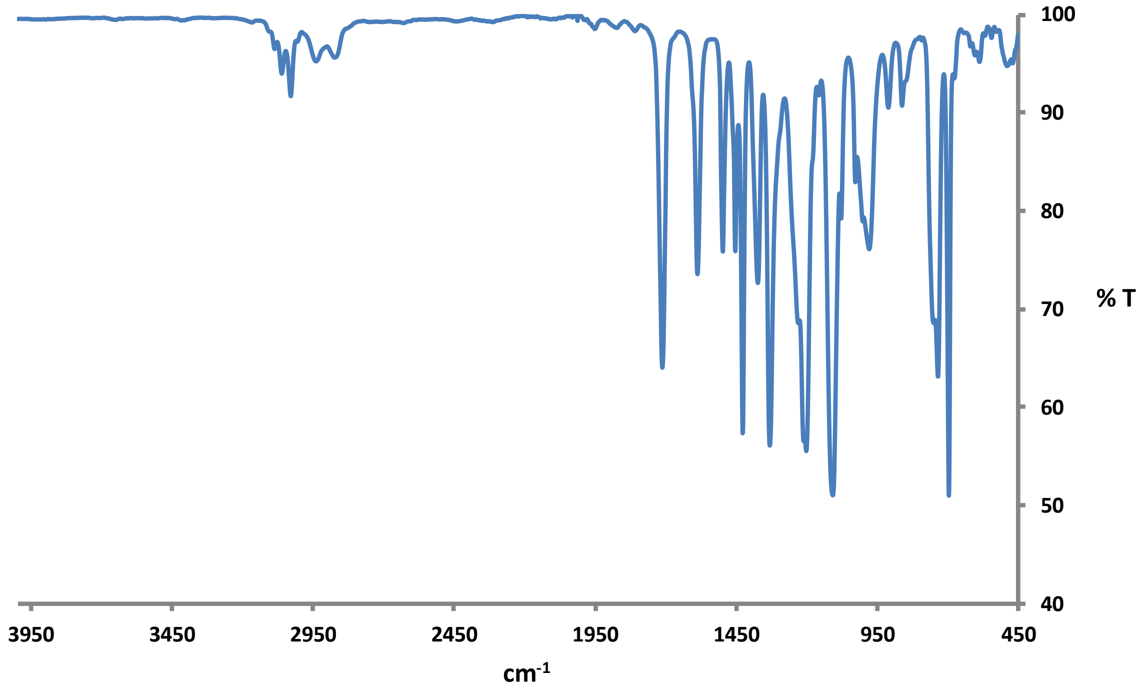

Figure S3. IR spectrum of benzyl 3,4,5-tris(benzyloxy)benzoate.

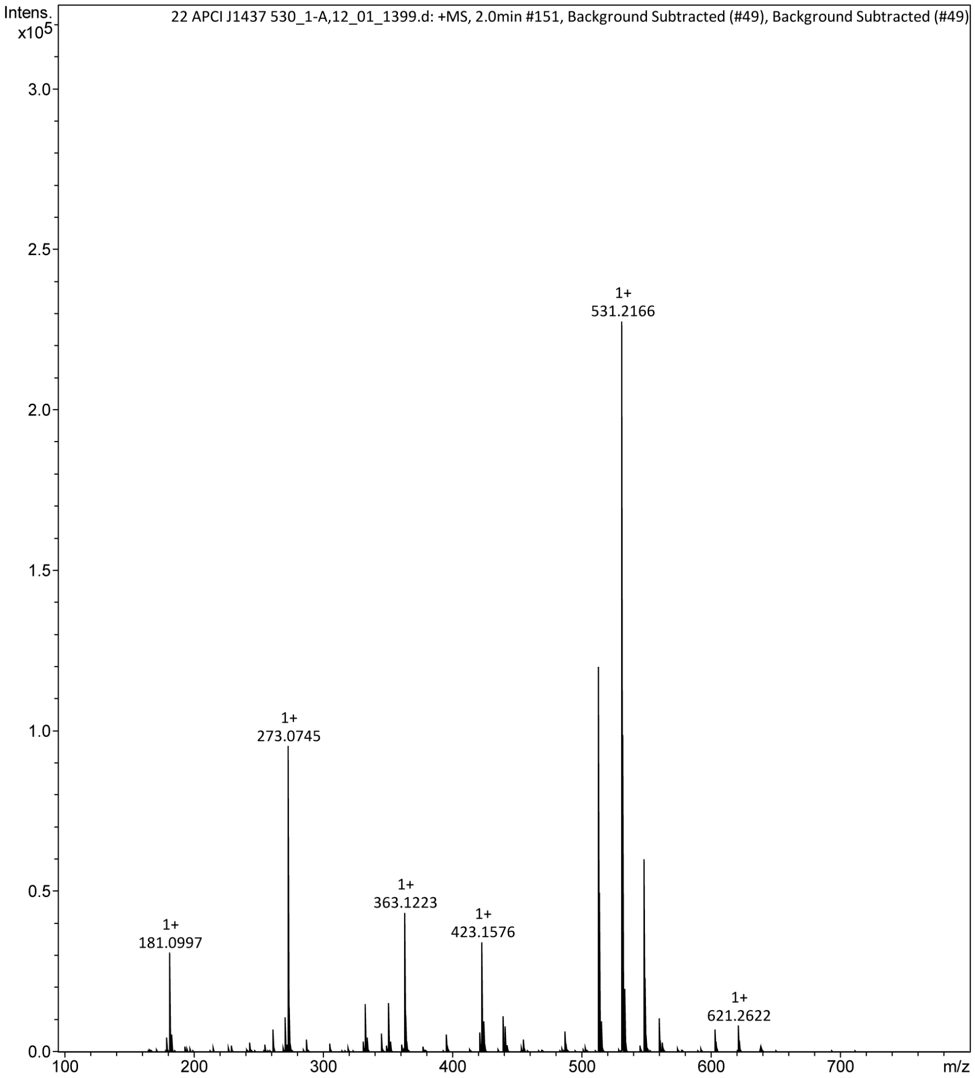

Figure S4. HR-MS of benzyl 3,4,5-tris(benzyloxy)benzoate.

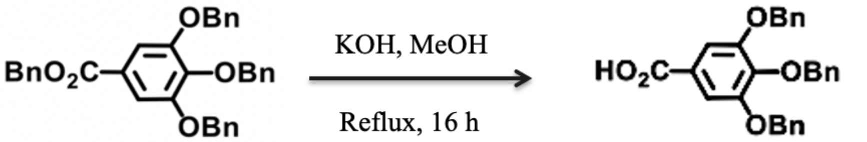

3,4,5-tris(benzyloxy)benzoic acid

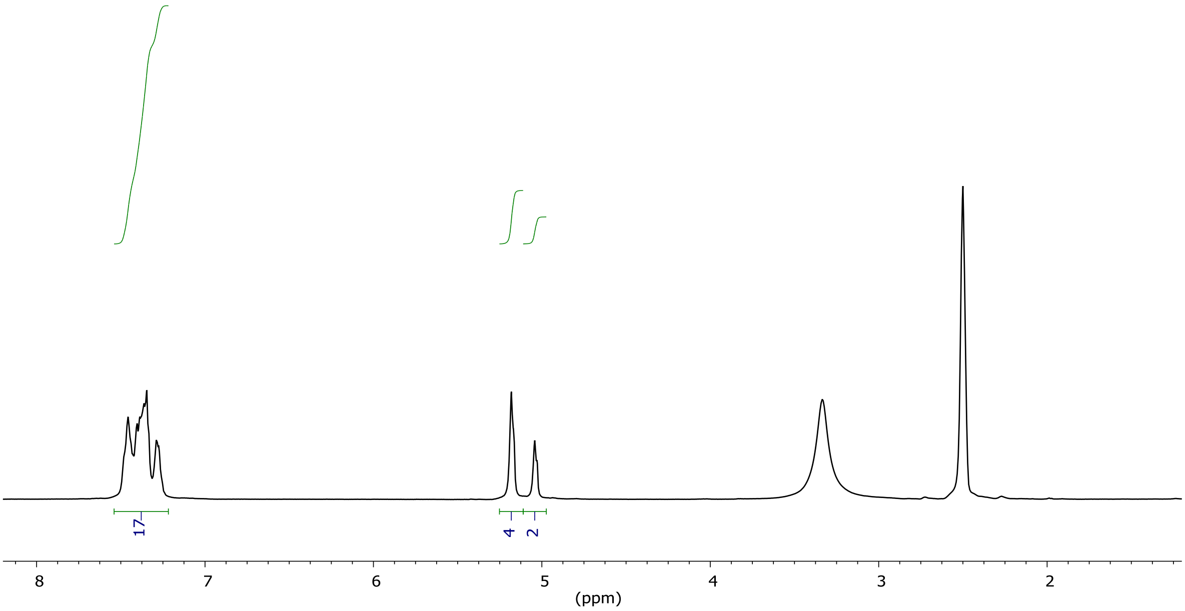

Figure S5. ^{1}H NMR spectrum (DMSO-d_6) of 3,4,5-tris(benzyloxy)benzoic acid.

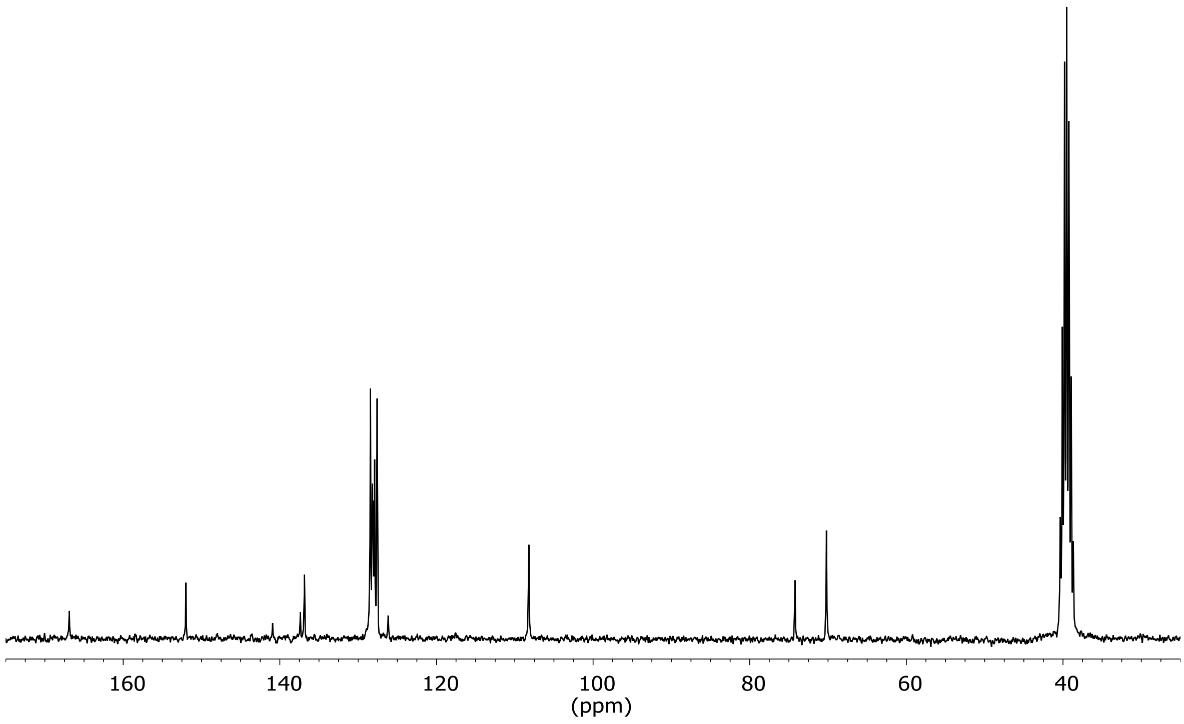

Figure S6. ^{13}C NMR spectrum (DMSO-d_6) of 3,4,5-tris(benzyloxy)benzoic acid.

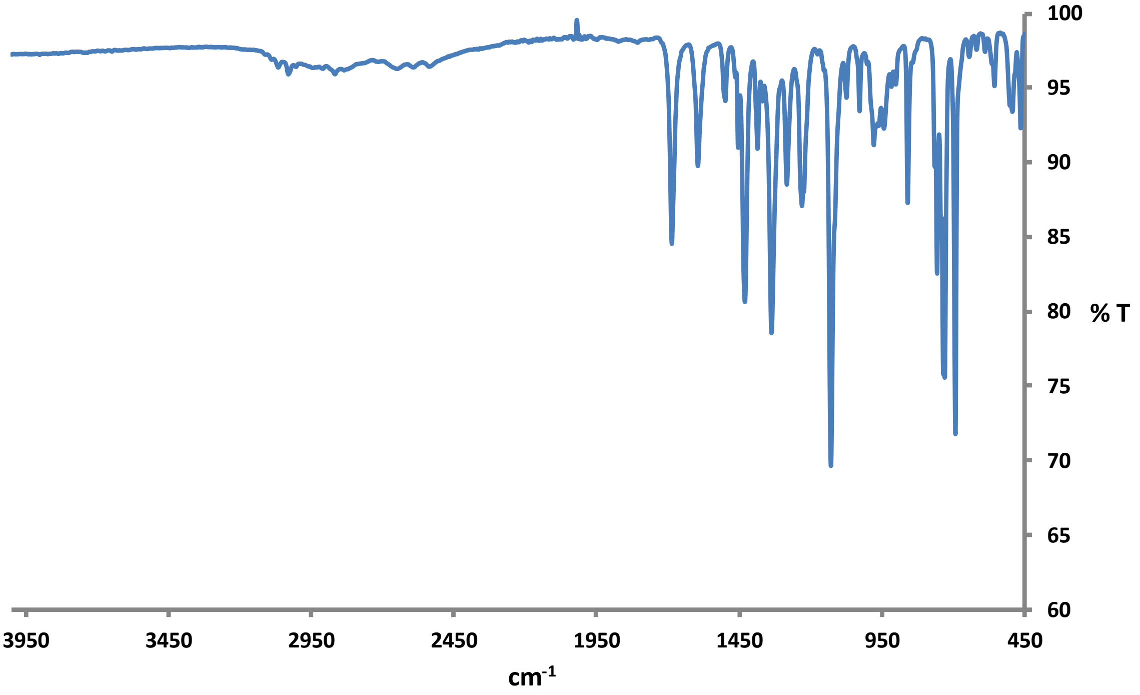

Figure S7. IR spectrum of 3,4,5-tris(benzyloxy)benzoic acid.

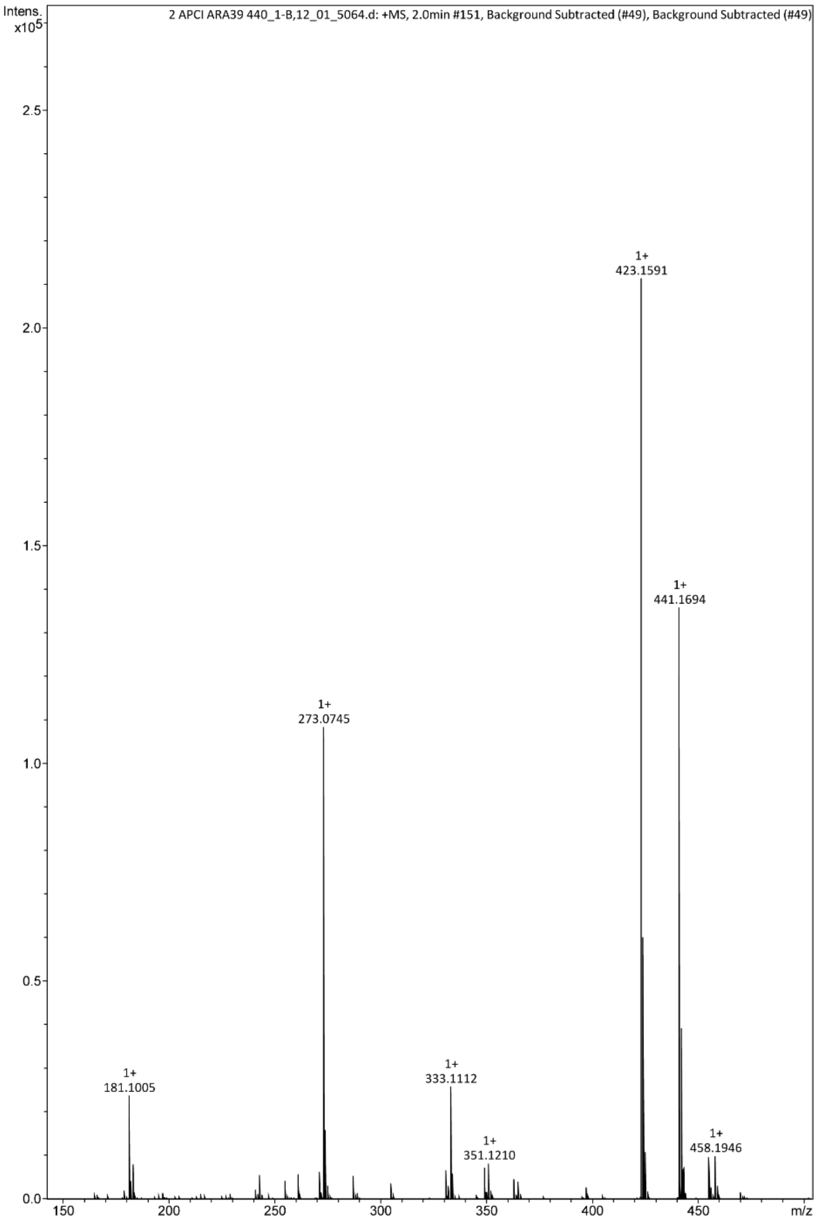

Figure S8. HR-MS of 3,4,5-tris(benzyloxy)benzoic acid.

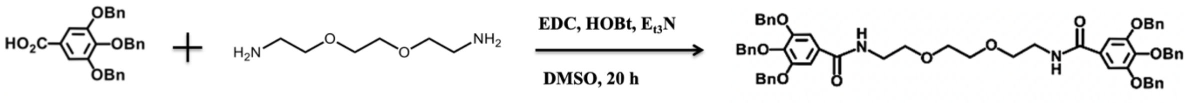

2[G0]-GaOBn.

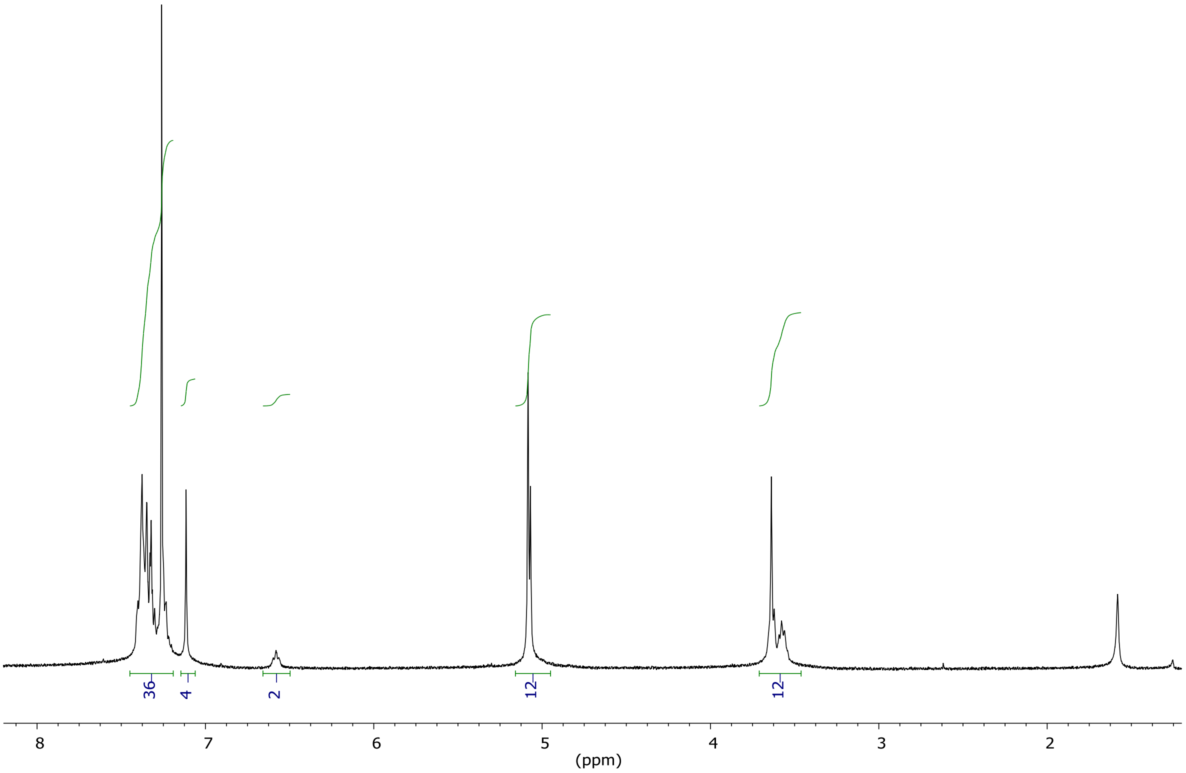

Figure S9. ^{1}H NMR spectrum ($CDCl_3$) of 2[G0]-GaOBn.

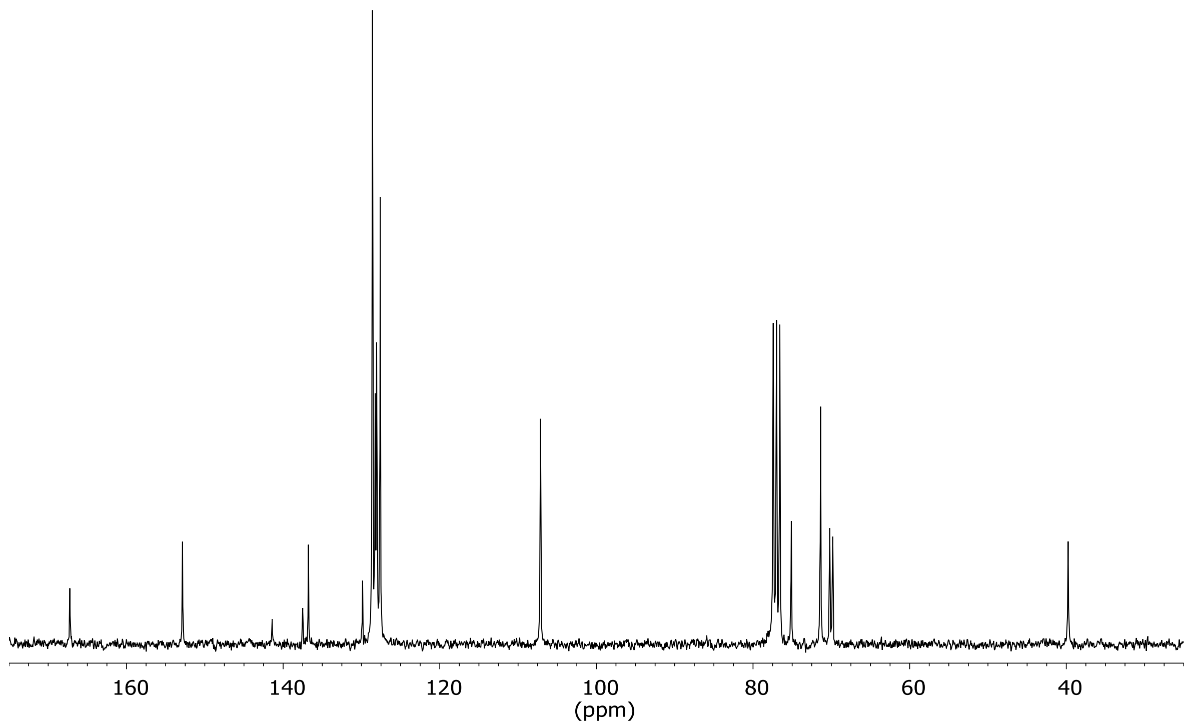

Figure S10. ^{13}C NMR spectrum ($CDCl_3$) of 2[G0]-GaOBn.

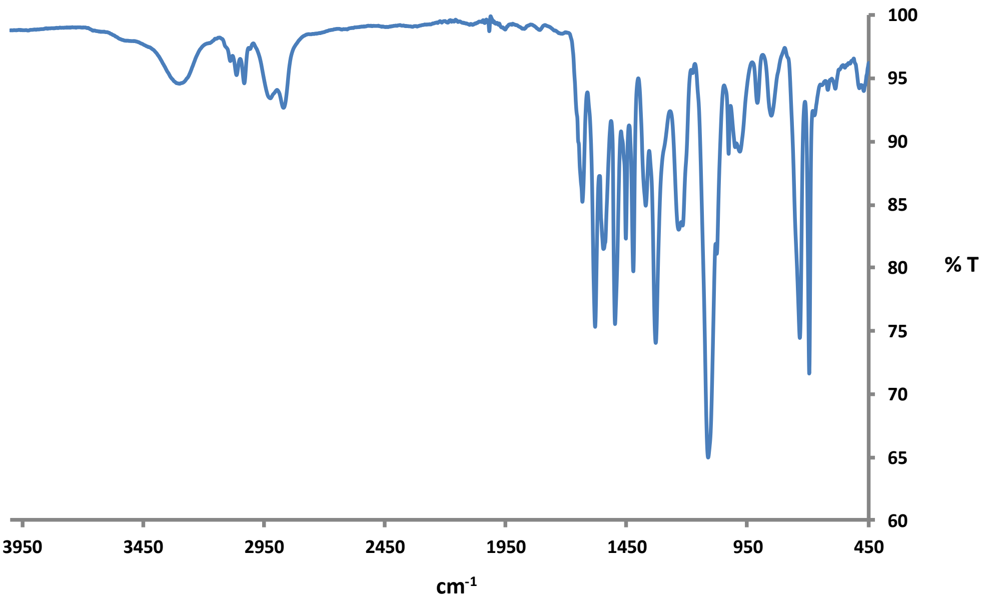

Figure S11. IR spectrum of 2[G0]-GaOBn.

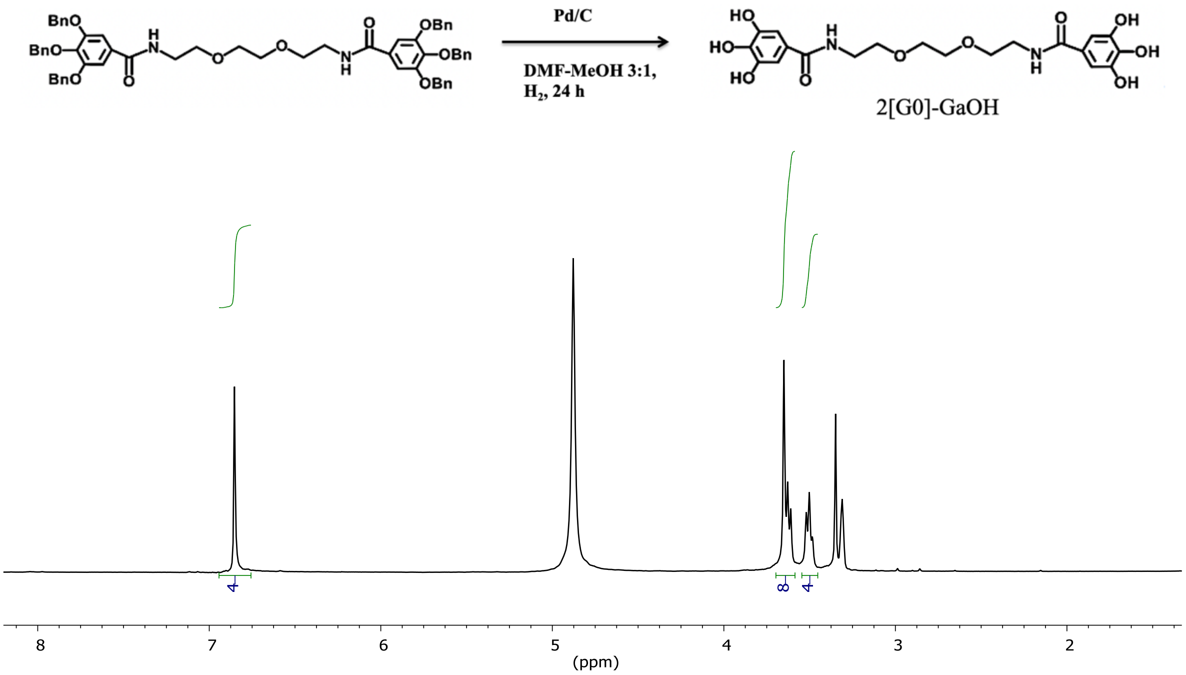

Figure S12. ^{1}H NMR spectrum (CD_3OD) of 2[G0]-GaOH.

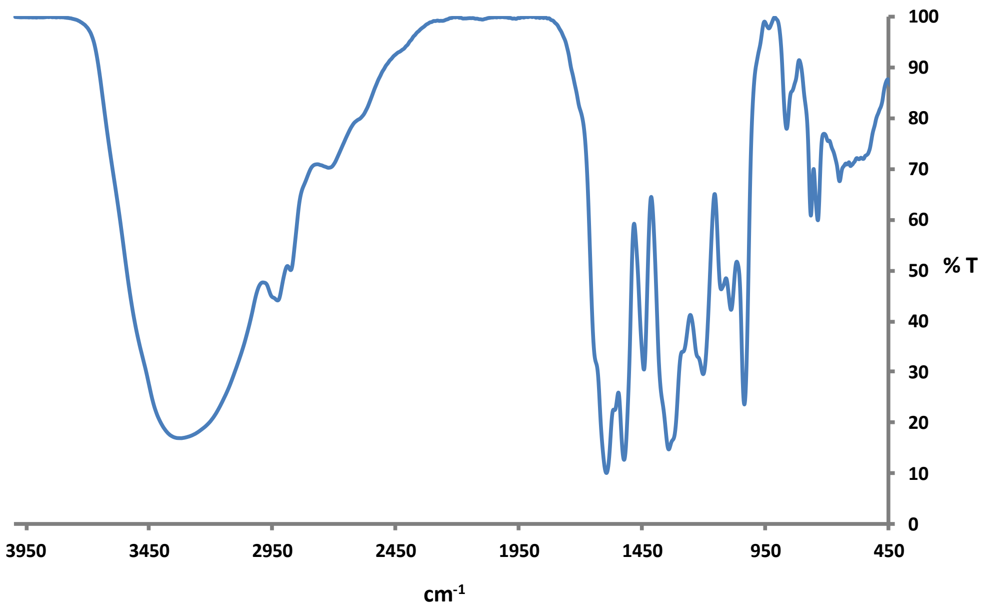

Figure S13. IR spectrum of 2[G0]-GaOH.

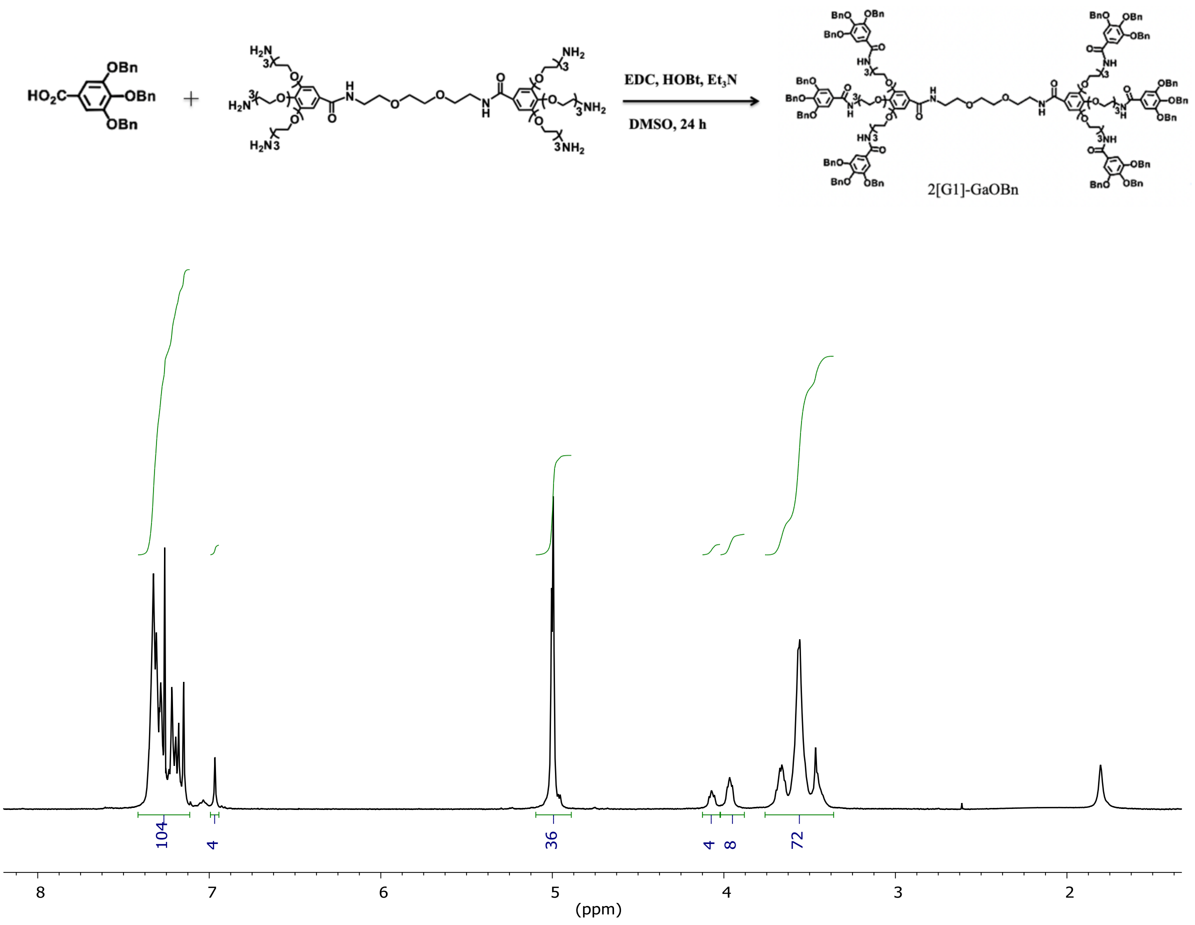

Figure S14. ^1H NMR spectrum ($CDCl_3$) of 2[G1]-GaOBn.

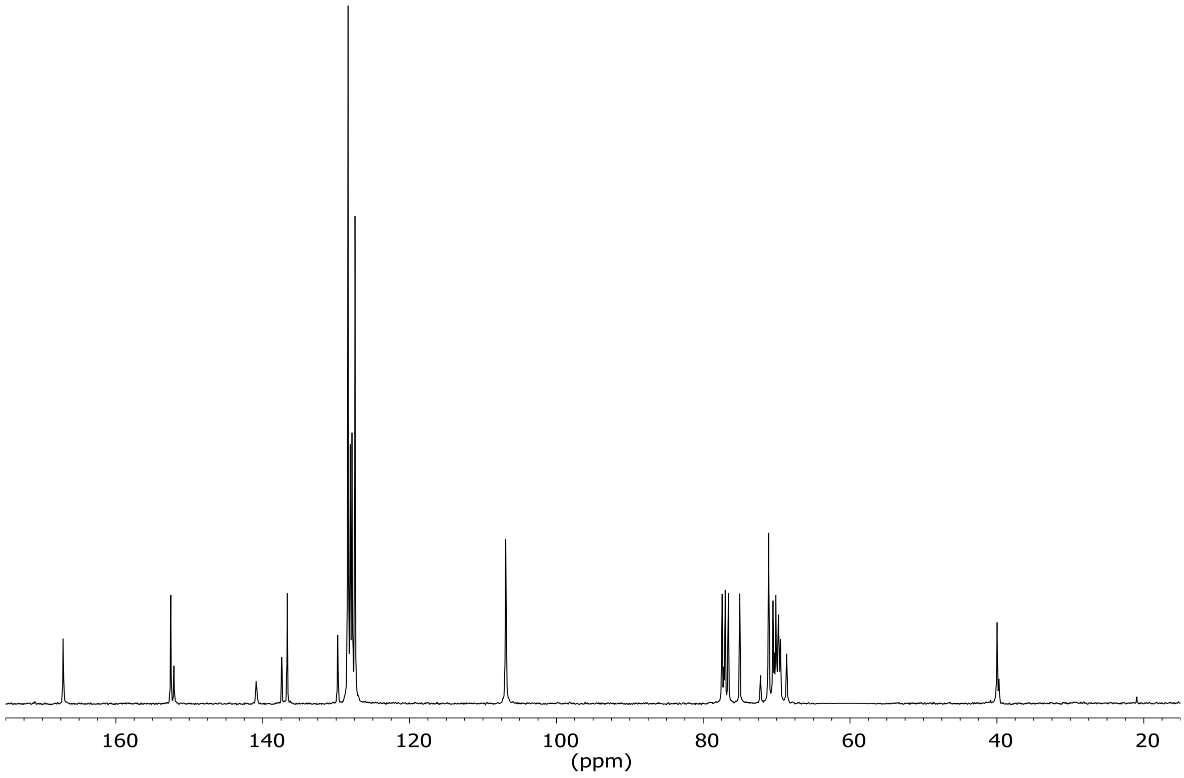

Figure S15. ^{13}C NMR spectrum ($CDCl_3$) of 2[G1]-GaOBn.

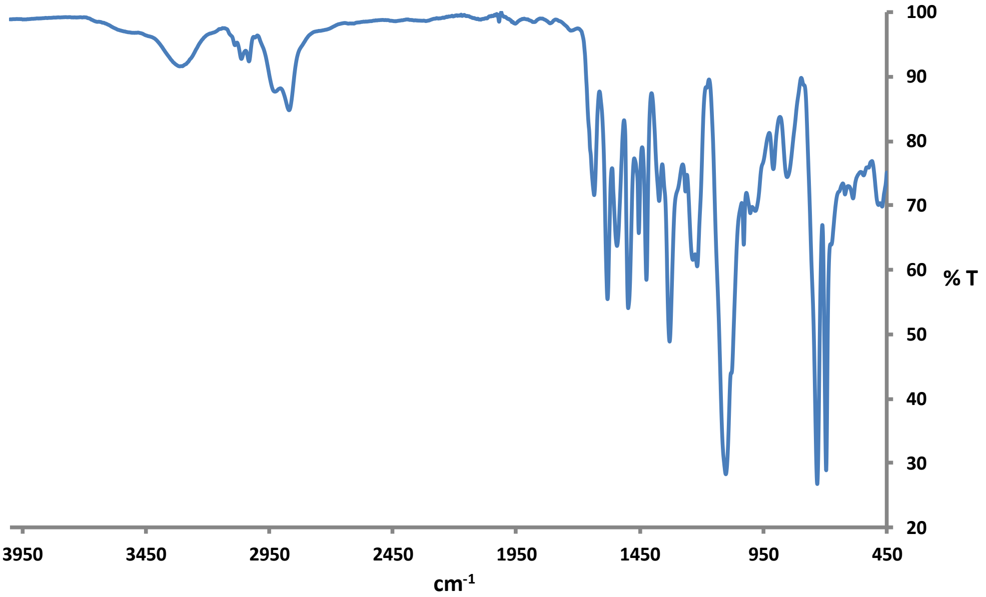

Figure S16. IR spectrum of 2[G1]-GaOBn.

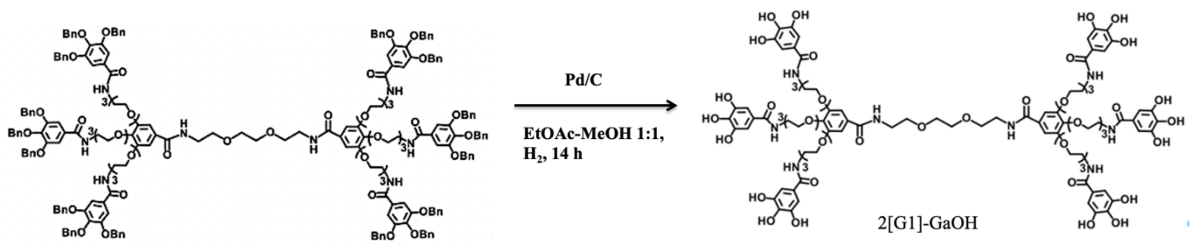

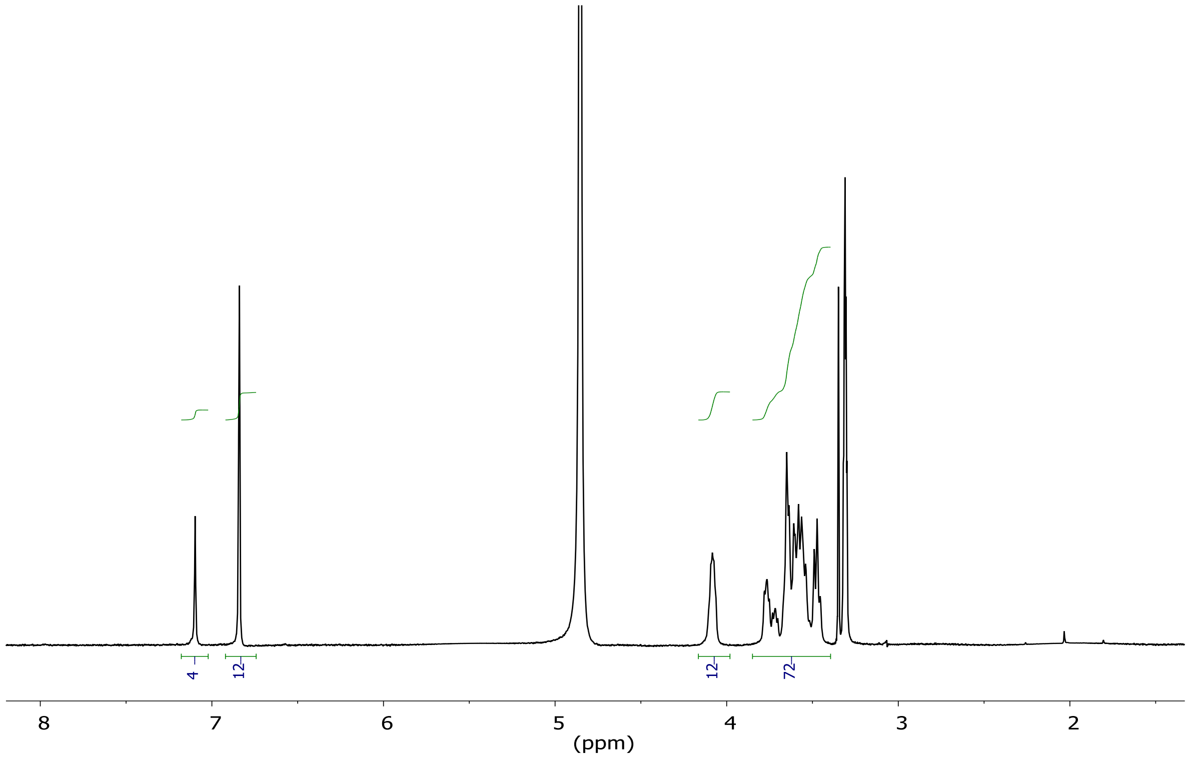

Figure S17. ^{1}H NMR spectrum (CD_3OD) of 2[G1]-GaOH.

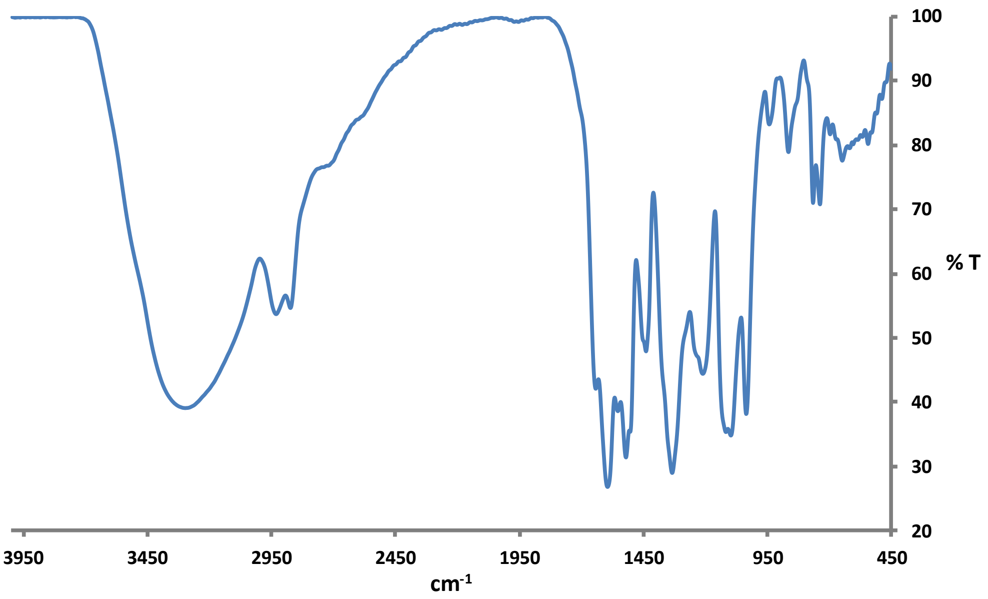

Figure S18. IR spectrum of 2[G1]-GaOH.

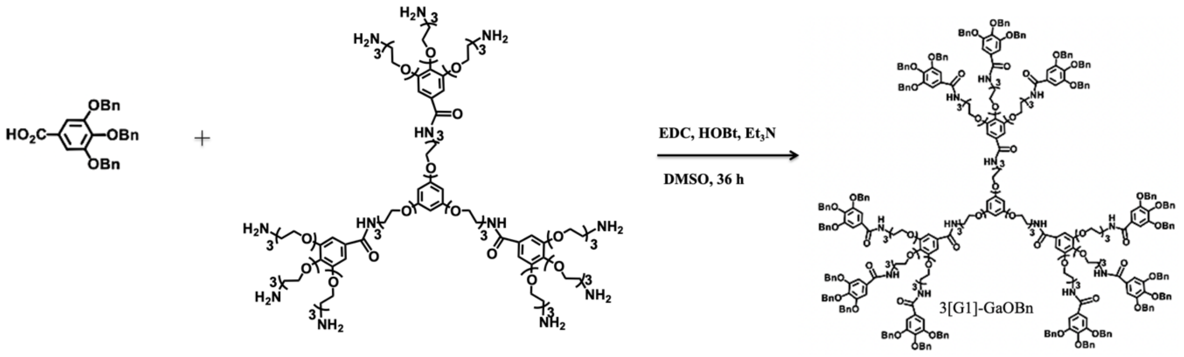

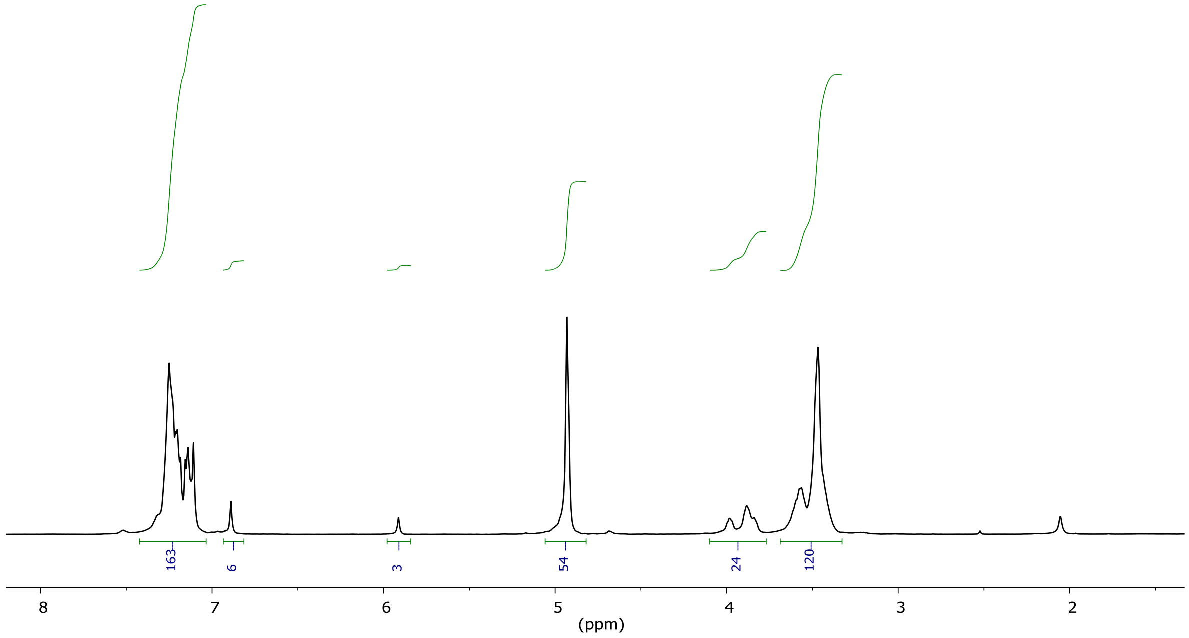

Figure S19. ^{1}H NMR spectrum ($CDCl_3$) of 3[G1]-GaOBn.

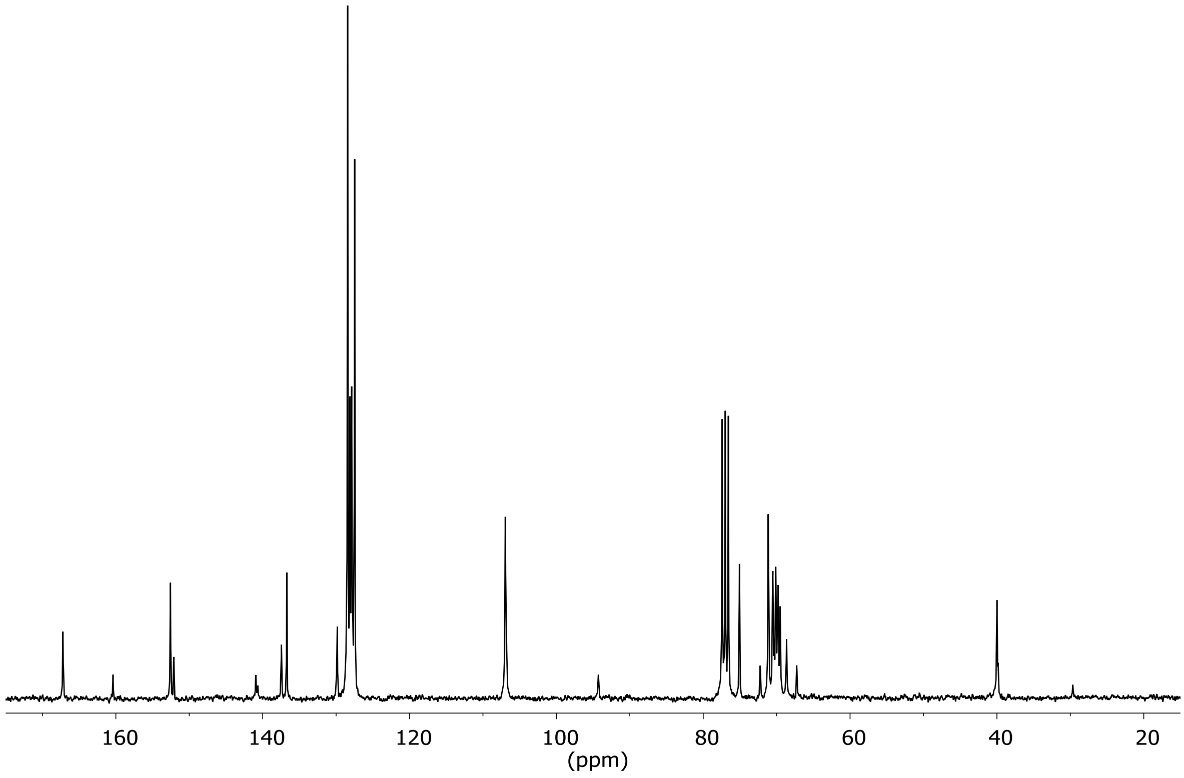

Figure S20. ^{13}C NMR spectrum ($CDCl_3$) of 3[G1]-GaOBn.

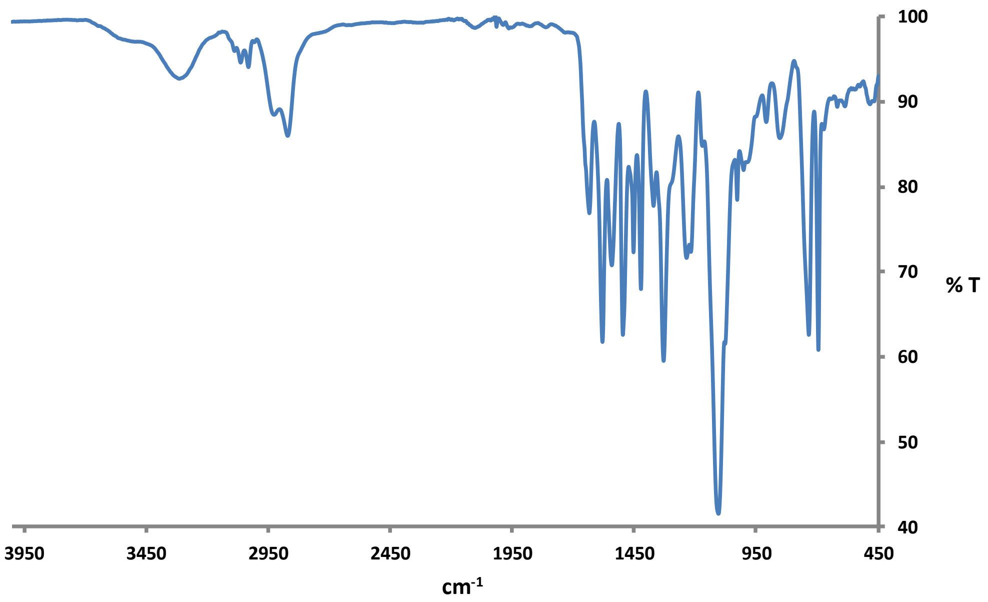

Figure S21. IR spectrum of 3[G1]-GaOBn.

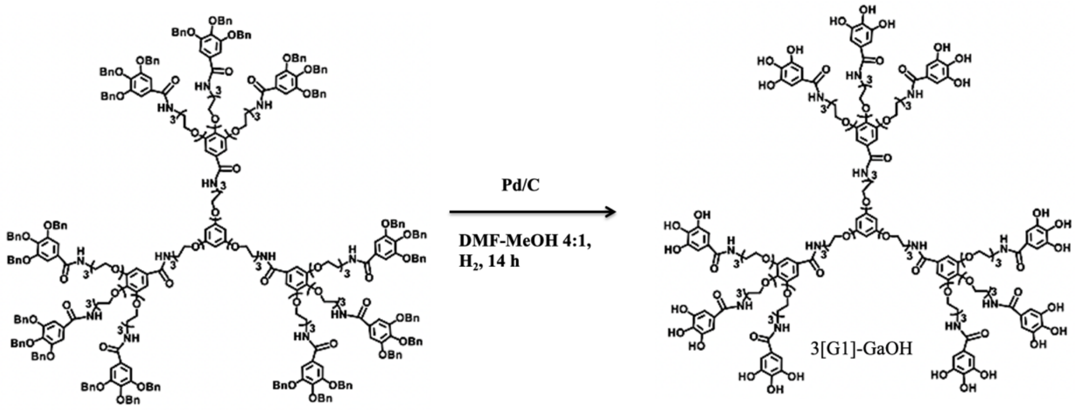

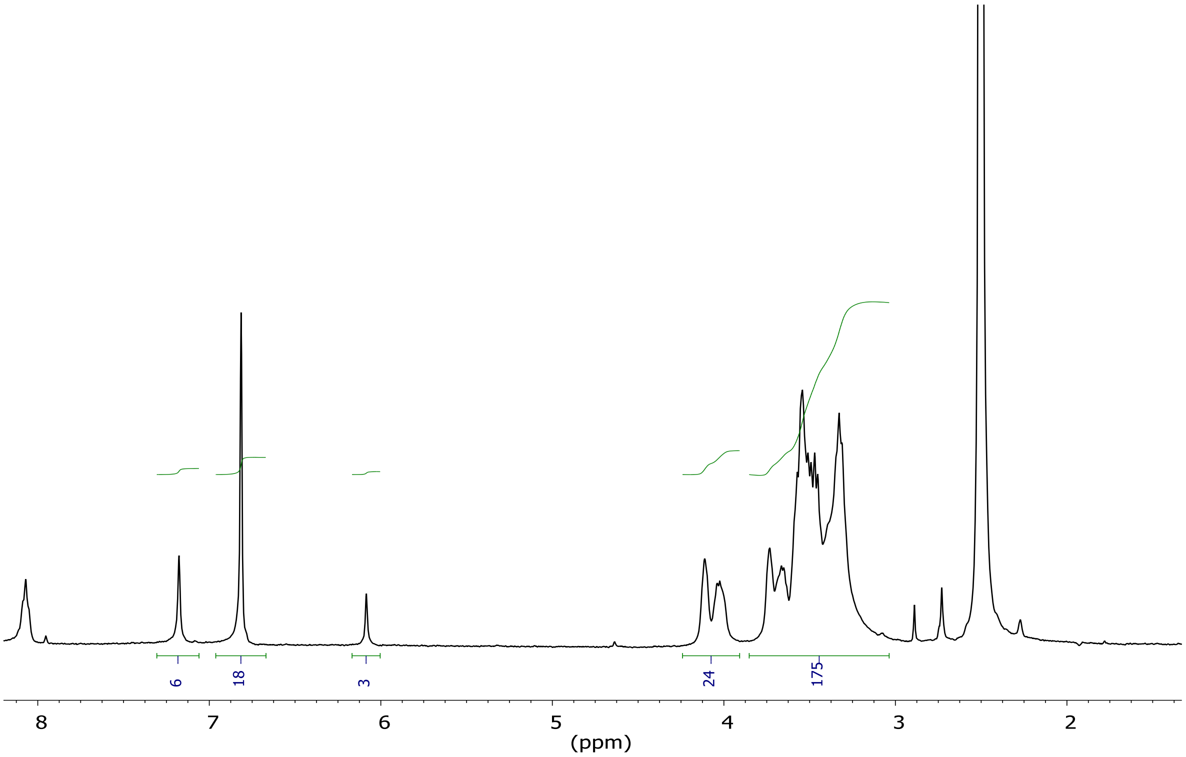

Figure S22. ^{1}H NMR spectrum (DMSO-d_6) of 3[G1]-GaOH.

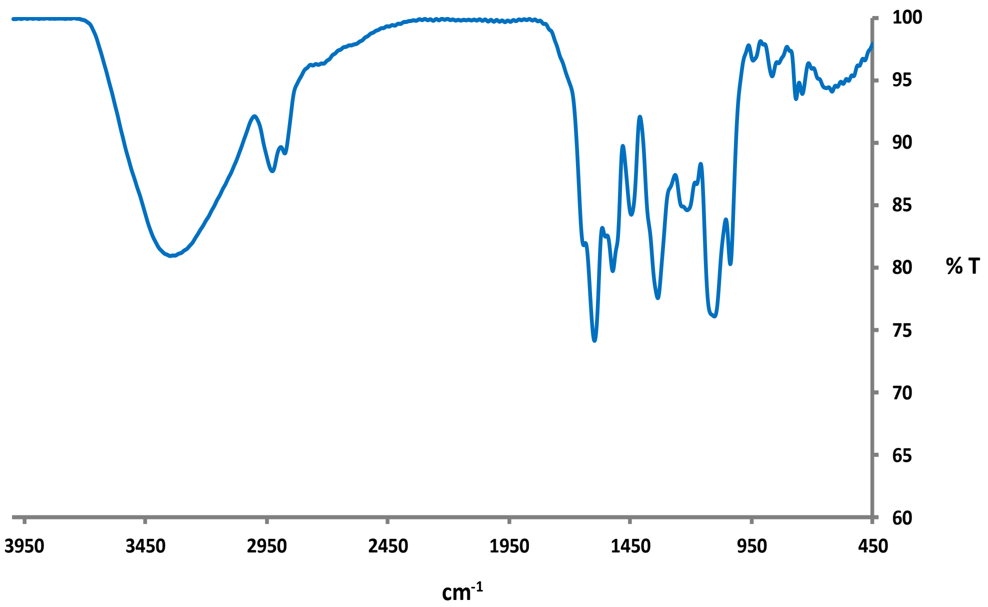

Figure S23. IR spectrum of 3[G1]-GaOH.

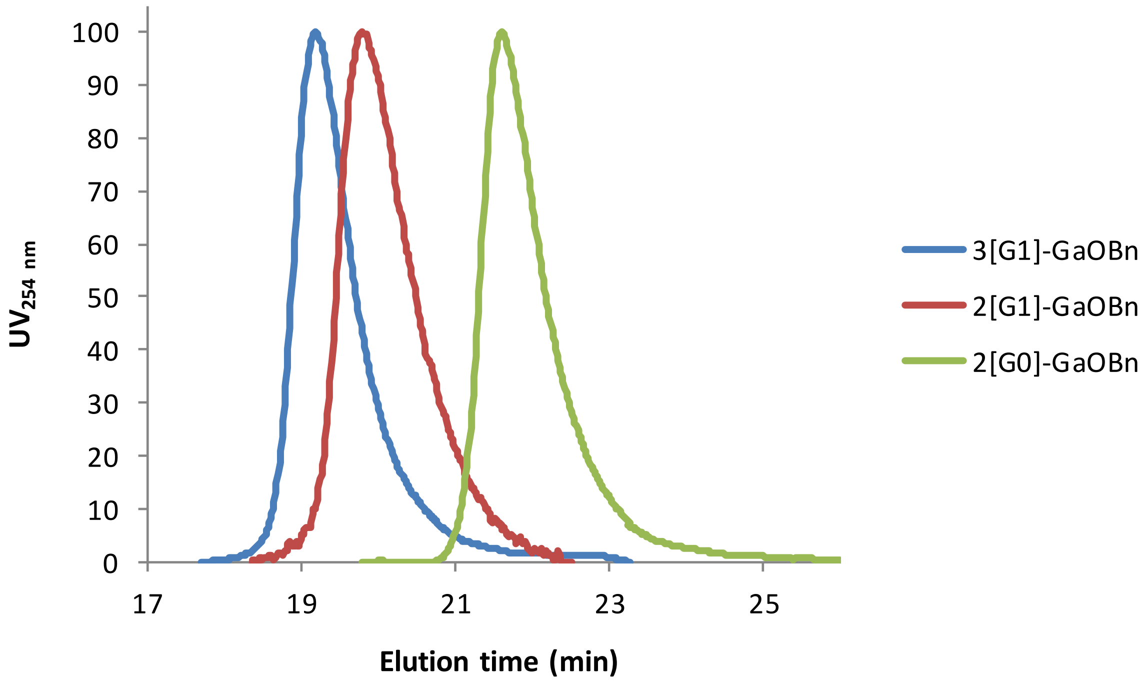

Figure S24. GPC elugrams of x[Gn]-GaOBn (THF).

Interference of the dendrimers in the Aβ supramolecular assembly

Molecular dynamics simulations of the dendrimer-Aβ interactions

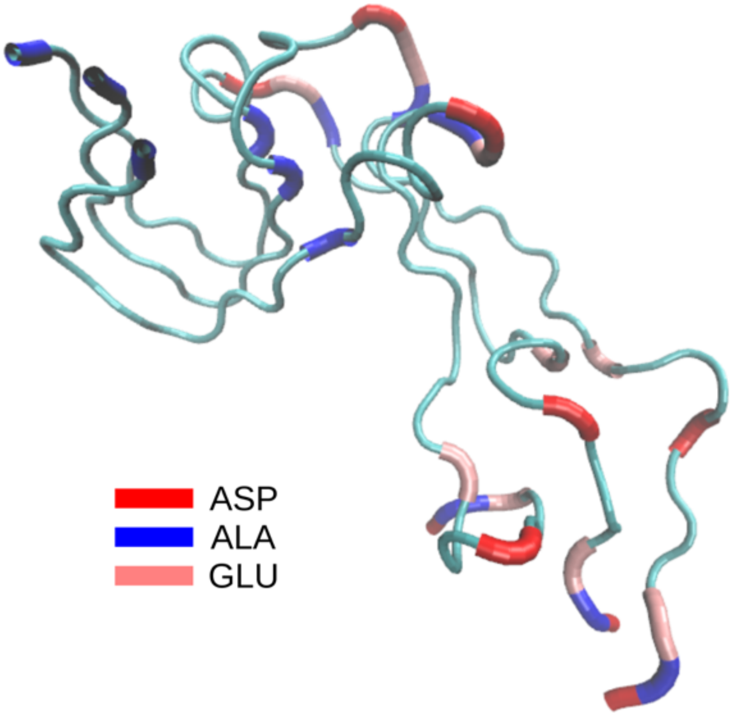

Figure S25. Optimized geometry from MD simulations of 2NAO model (full-length Aβf).

Table S1. MD simulation analysis of the interaction between bull-length $A\beta_f$ (2NAO pdb file) and dendrimers under a $A\beta_f$:dendrimers ratio of 1:1 showing the average number of H-bonds between the peptide chains of $A\beta_f$ and the corresponding dendrimer, as well as the solvent-accessible surface area (SASA) of the region where dendrimers are interacting.

**Disassembly 1:1**	**Net H-Bond (number #)**	**SASA (nm^2)**
**2NAO**	--	145 ± 4
**2NAO - Ga**	23 ± 3	141 ± 4
**2NAO - 2G0-GaOH**	29 ± 3	147 ± 5
**2NAO - 2G1-GaOH**	45 ± 4	153 ± 4
**2NAO - 3G1-GaOH**	64 ± 4	113 ± 3

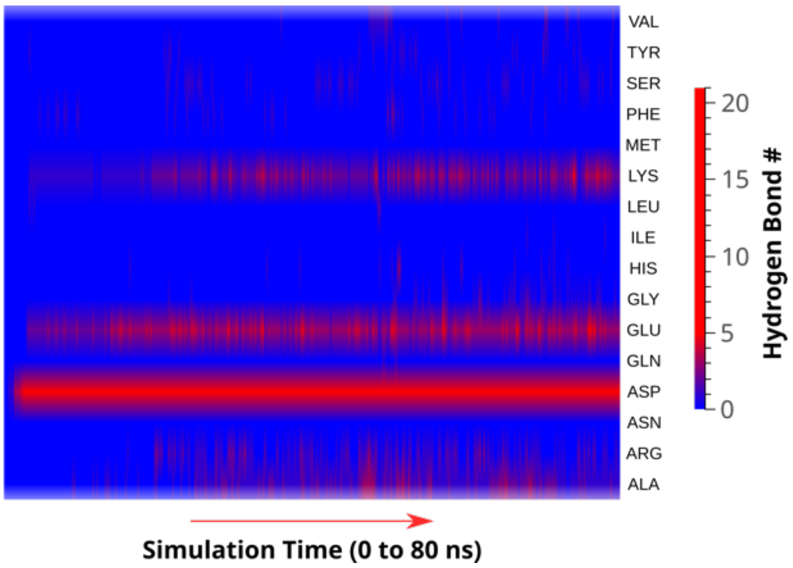

Figure S26. Dynamic interaction energy between full-length Aβ_f (2NAO pdb file) and Ga (1:1 ratio). Preferential binding residues were calculated by the nonbonded interaction energy and the number of H-bonds between each Aβ_f and Ga.

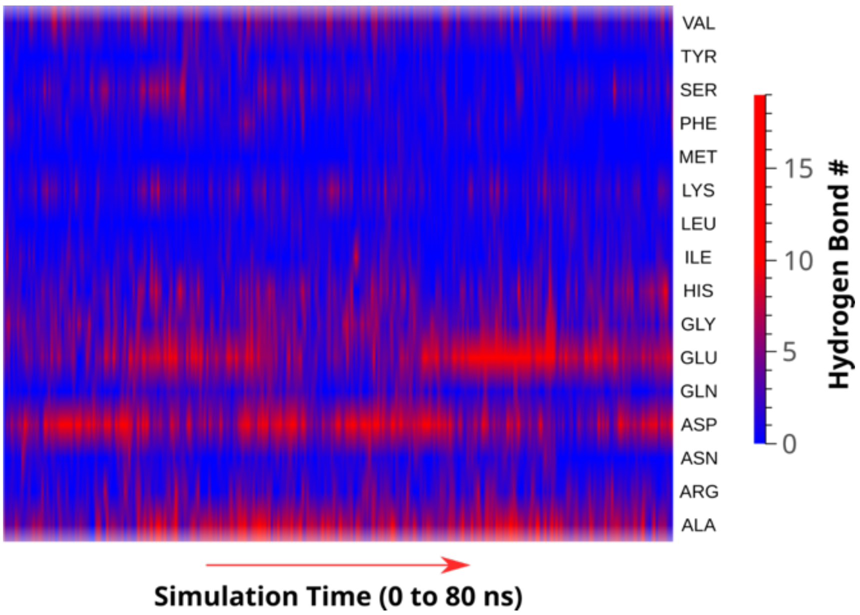

Figure S27. Dynamic interaction energy between full-length Aβ_f (2NAO pdb file) and 2G0-GaOH (1:1 ratio). Preferential binding residues were calculated by the nonbonded interaction energy and the number of H-bonds between each full-length Aβ_f and 2G0-GaOH.

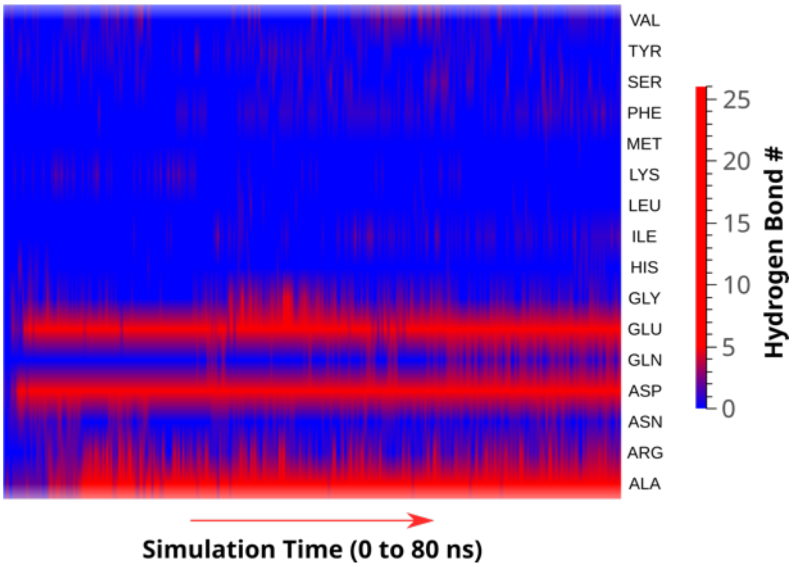

Figure S28. Dynamic interaction energy between full-length Aβ_f (2NAO pdb file) and 2G1-GaOH (1:1 ratio). Preferential binding residues were calculated by the nonbonded interaction energy and the number of H-bonds between each full-length Aβ_f and 2G1-GaOH.

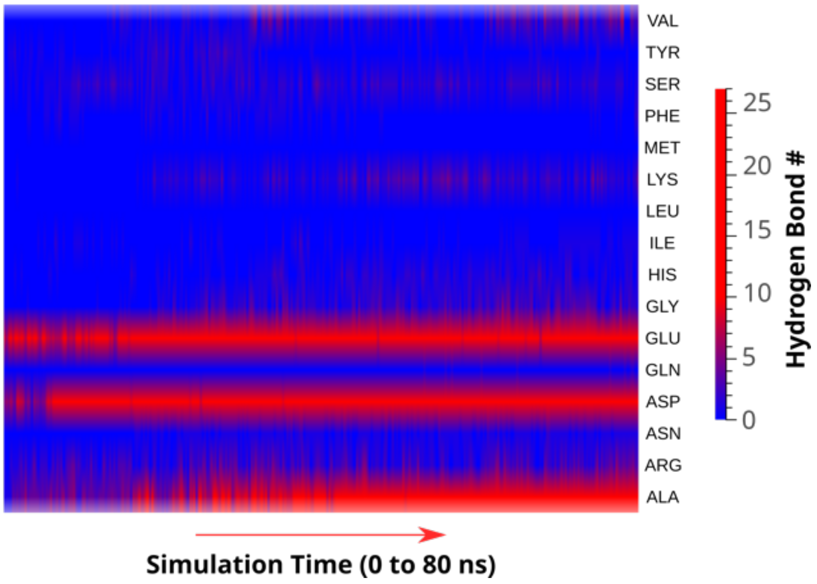

Figure S29. Dynamic interaction energy between full-length Aβ_f (2NAO pdb file) and 3G1-GaOH (1:1 ratio). Preferential binding residues were calculated by the nonbonded interaction energy and the number of H-bonds between each full-length Aβ_f and 3G1-GaOH.

Thioflavin T (ThT) assay

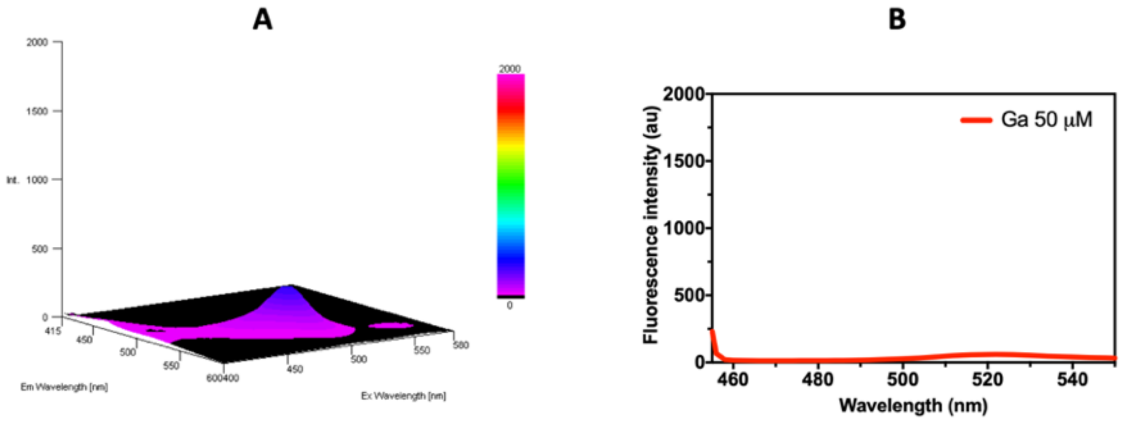

Figure S30. (A) 3D fluorescence spectra of Gallic acid (Ga, 50μM) using the following acquisition parameters: λ_{ex} = 400-580 nm; λ_{em} = 415-600 nm; (B) Fluorescence spectrum of Ga (50μM) acquired using a λ_{ex} = 435 nm and a λ_{em} = 445-600 nm; excitation bandwidth = 5nm; emission bandwidth = 10nm; response = 2s.

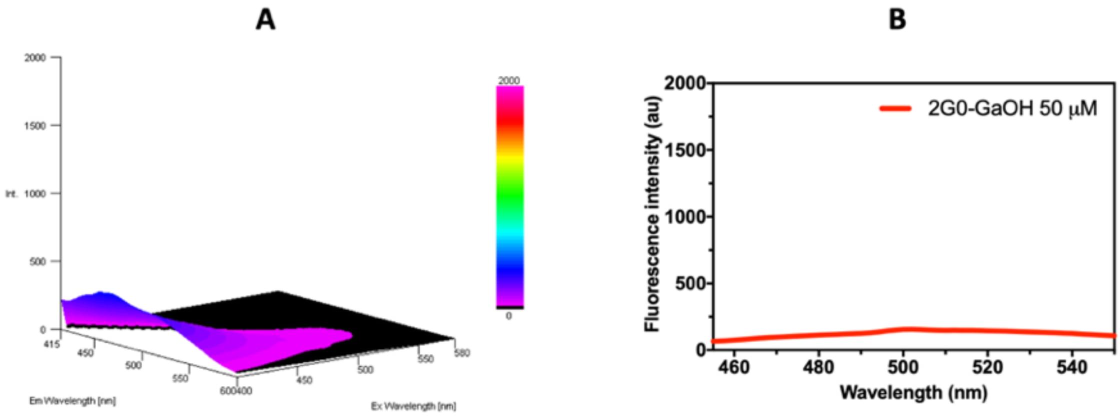

Figure S31. (A) 3D fluorescence spectra of 2G0-GaOH (50μM) using the following acquisition parameters: λ_{ex} = 400-580 nm; λ_{em} = 415-600 nm; (B) Fluorescence spectrum of 2G0-GaOH (50μM) acquired using a λ_{ex} = 435 nm and a λ_{em} = 445-600 nm; excitation bandwidth = 5nm; emission bandwidth = 10nm; response = 2s.

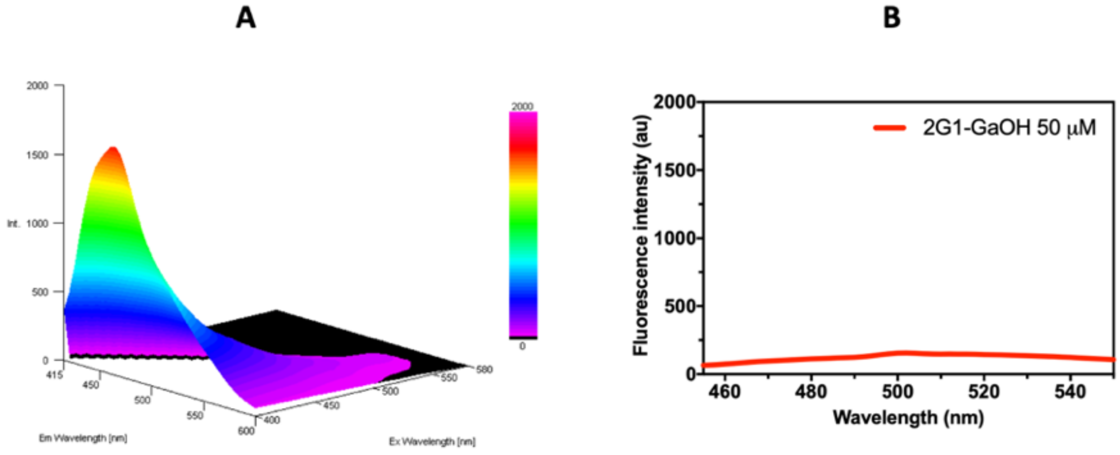

Figure S32. (A) 3D fluorescence spectra of 2G1-GaOH (50μM) using the following acquisition parameters: λ_{ex} = 400-580 nm; λ_{em} = 415-600 nm; (B) Fluorescence spectrum of 2G1-GaOH (50μM) acquired using a λ_{ex} = 435 nm and a λ_{em} = 445-600 nm; excitation bandwidth = 5nm; emission bandwidth = 10nm; response = 2s.

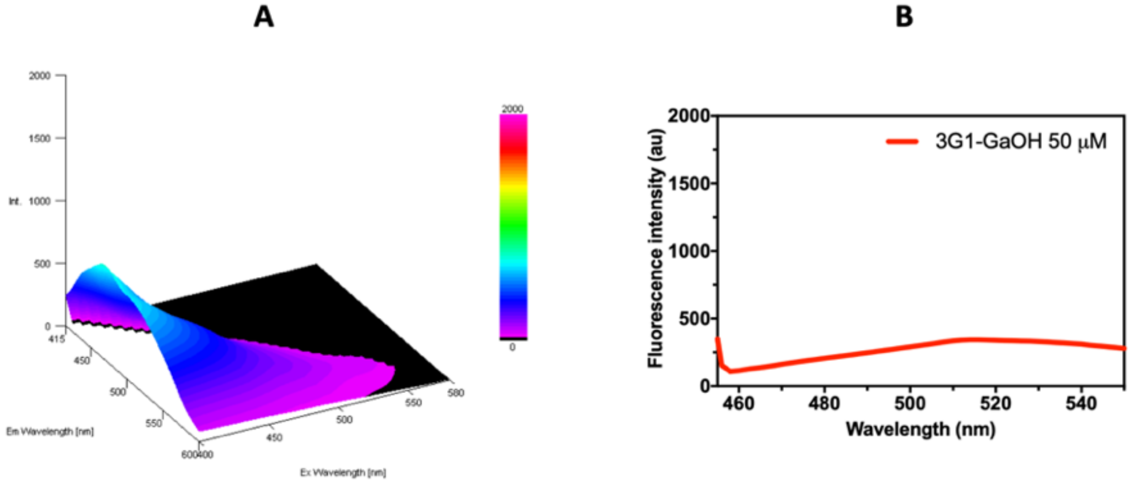

Figure S33. (A) 3D fluorescence spectra of 3G1-GaOH (50μM) using the following acquisition parameters: λ_{ex} = 400-580 nm; λ_{em} = 415-600 nm; (B) Fluorescence spectrum of 3G1-GaOH (50μM) acquired using a λ_{ex} = 435 nm and a λ_{em} = 445-600 nm; excitation bandwidth = 5nm; emission bandwidth = 10nm; response = 2s.

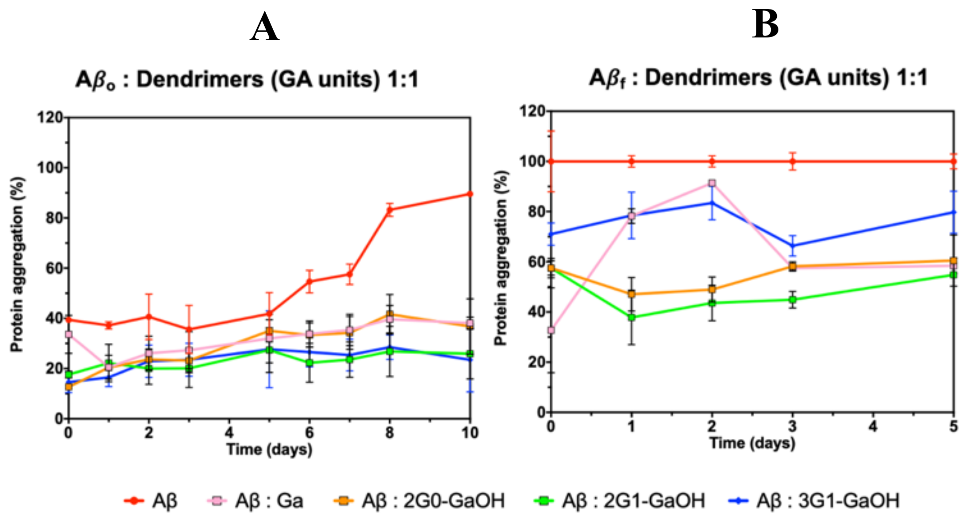

Figure S34. (A) Multivalent inhibition of the $A\beta_o$ aggregation kinetics evaluated using the ThT assay. Ga, 2G0-GaOH, 2G1-GaOH and 3G1-GaOH inhibited the Aβ fibril elongation at an Aβ:Ga molar ratio of 1:1. Dendrimers were mixed with $A\beta_o$ in the lag-phase of the Aβ fibril formation and fluorescence was measured over 10 days. All experiments were performed at room temperature. (B) Multivalent disassembling of $A\beta_f$ fibrils evaluated using the ThT assay. Ga, 2G0-GaOH, 2G1-GaOH and 3G1-GaOH promoted the disassembly of Aβ fibrils at an Aβ:Ga molar ratio of 1:1. Compounds were mixed with $A\beta_f$ at the plateau phase and fluorescence was measured over 5 days. All experiments were performed at room temperature.

Circular Dichroism (CD)

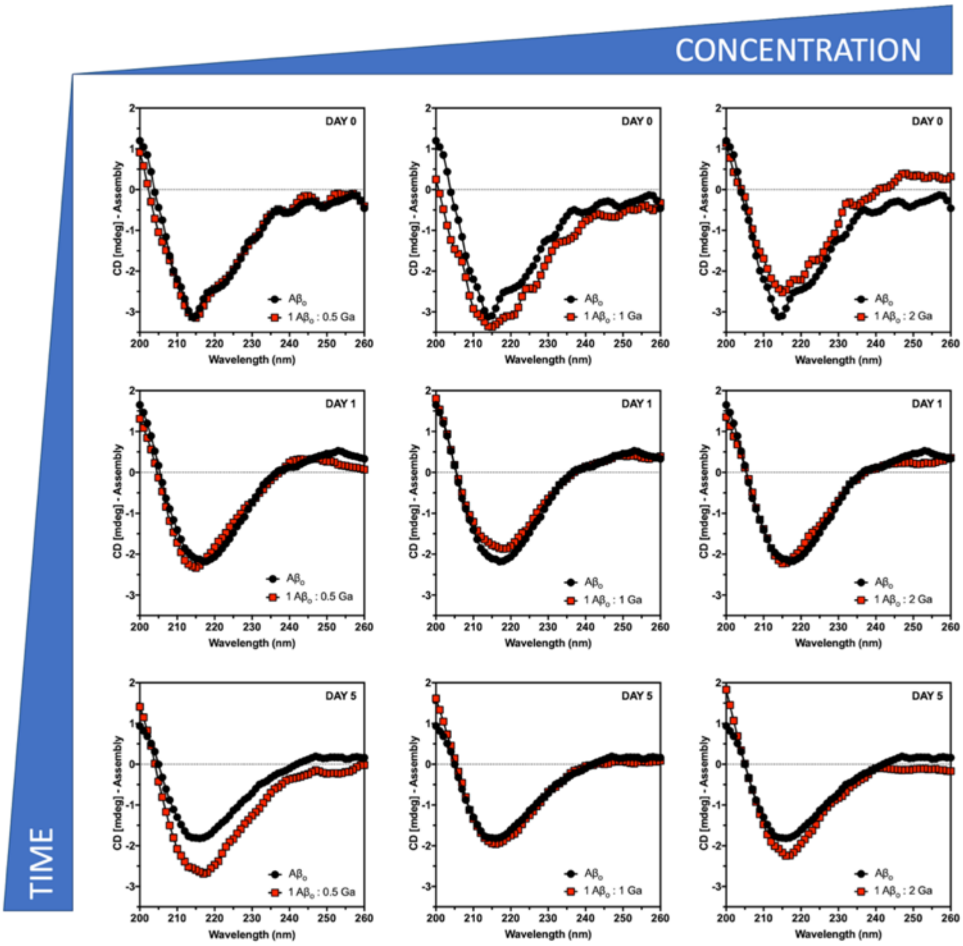

Figure S35. CD spectra of Aβo recorded for 5 days in the presence of different molar ratios of Ga. In all the cases, incubation was made at room temperature, under constant agitation.

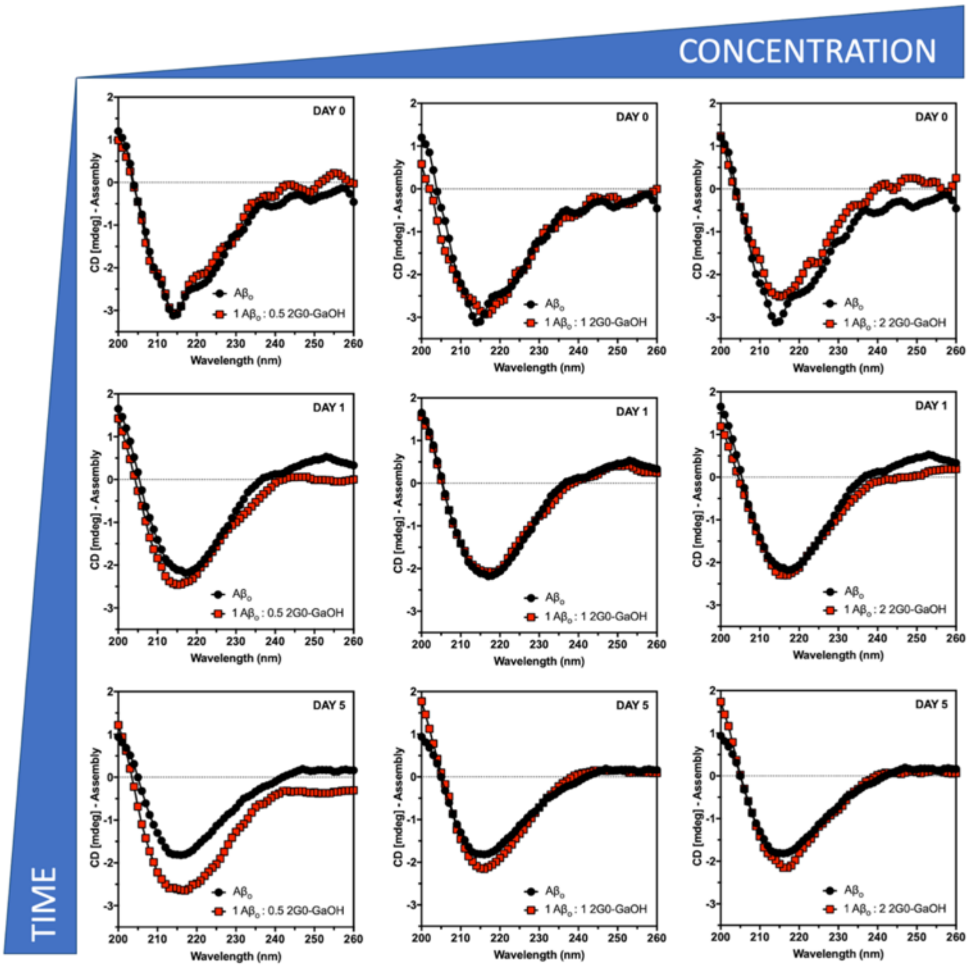

Figure S36. CD spectra of Aβo recorded for 5 days in the presence of different molar ratios of 2G0-GaOH. In all the cases, incubation was made at room temperature, under constant agitation.

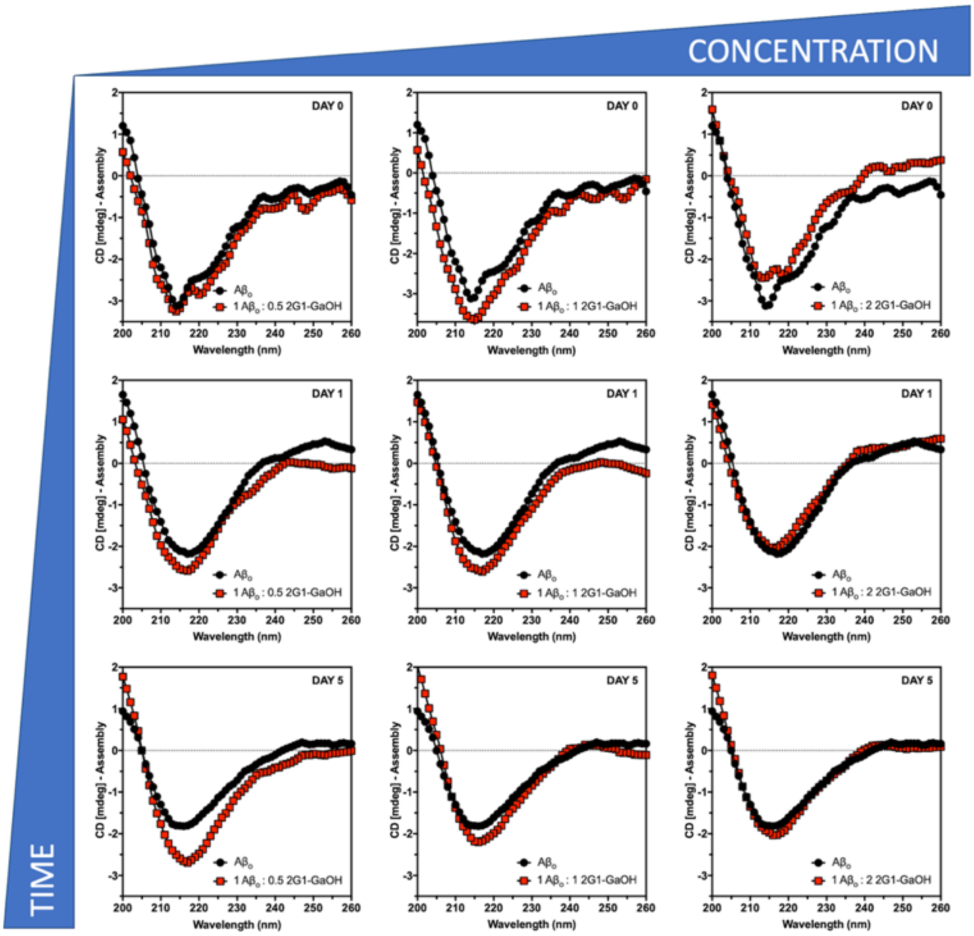

Figure S37. CD spectra of Aβₒ recorded for 5 days in the presence of different molar ratios of 2G1-GaOH. In all the cases, incubation was made at room temperature, under constant agitation.

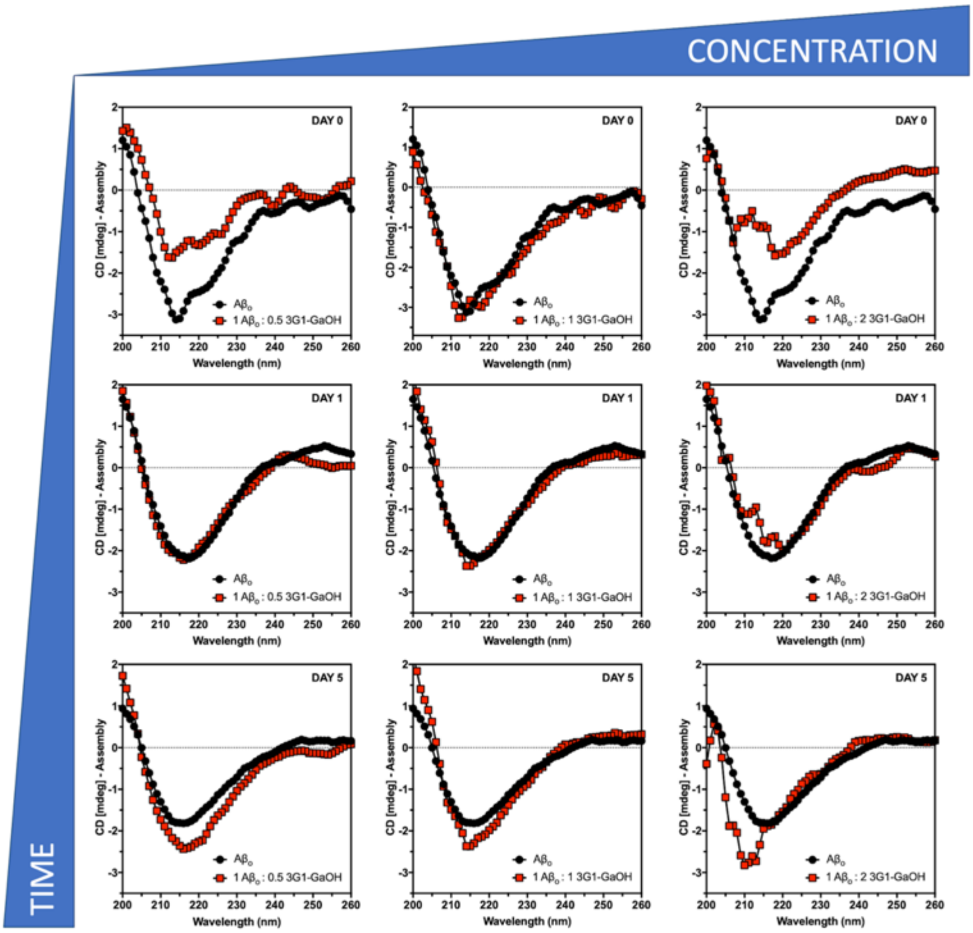

Figure S38. CD spectra of Aβₒ recorded for 5 days in the presence of different molar ratios of 3G1-GaOH. In all the cases, incubation was made at room temperature, under constant agitation.

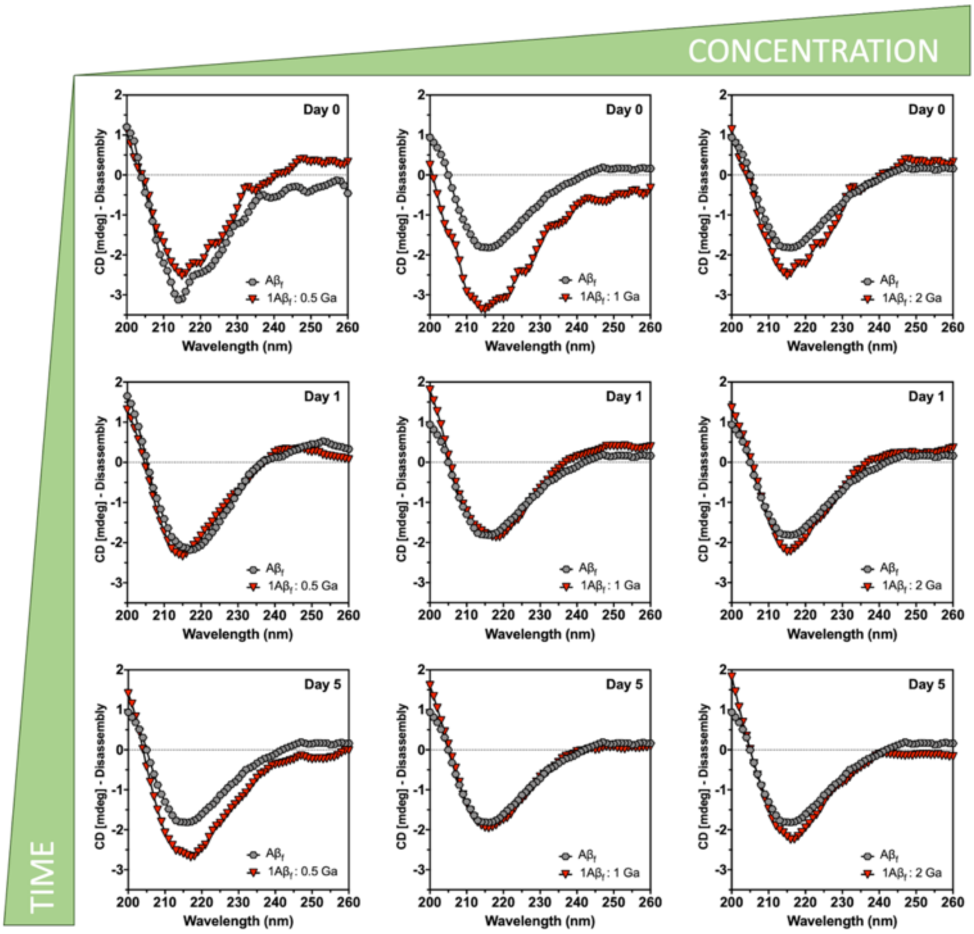

Figure S39. CD spectra of $A\beta_f$ recorded for 5 days in the presence of different molar ratios of Ga. In all the cases, incubation was made at room temperature, under constant agitation.

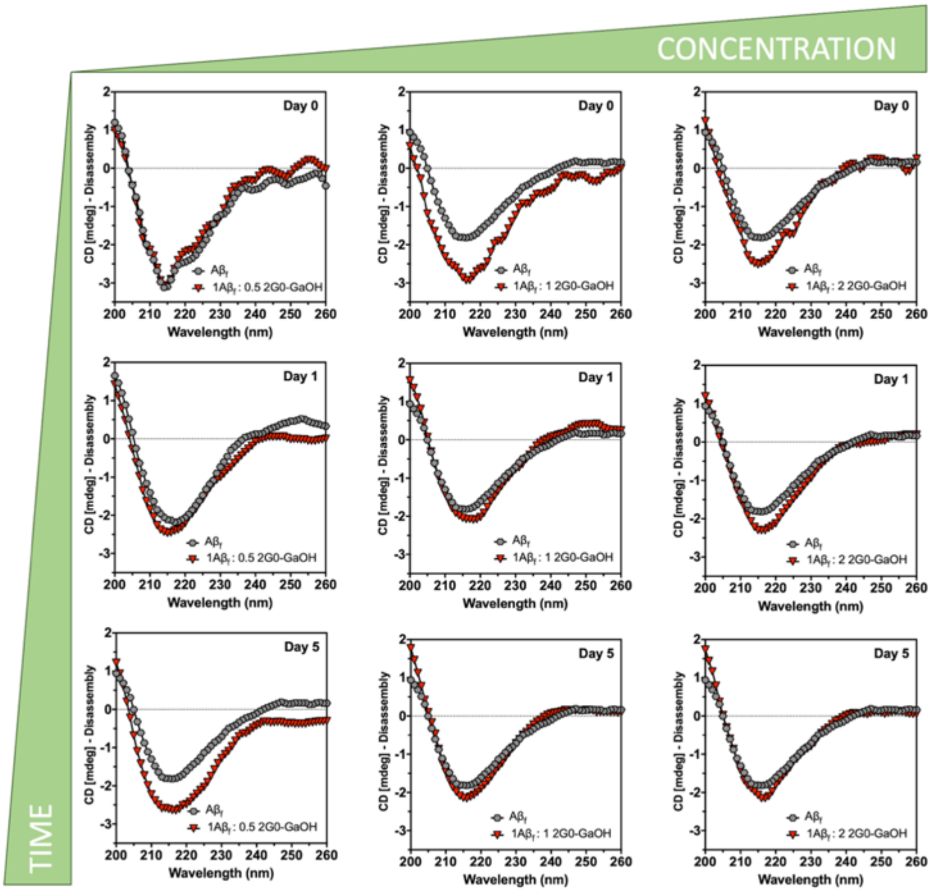

Figure S40. CD spectra of Aβ_f recorded for 5 days in the presence of different molar ratios of 2G0-GaOH. In all the cases, incubation was made at room temperature, under constant agitation.

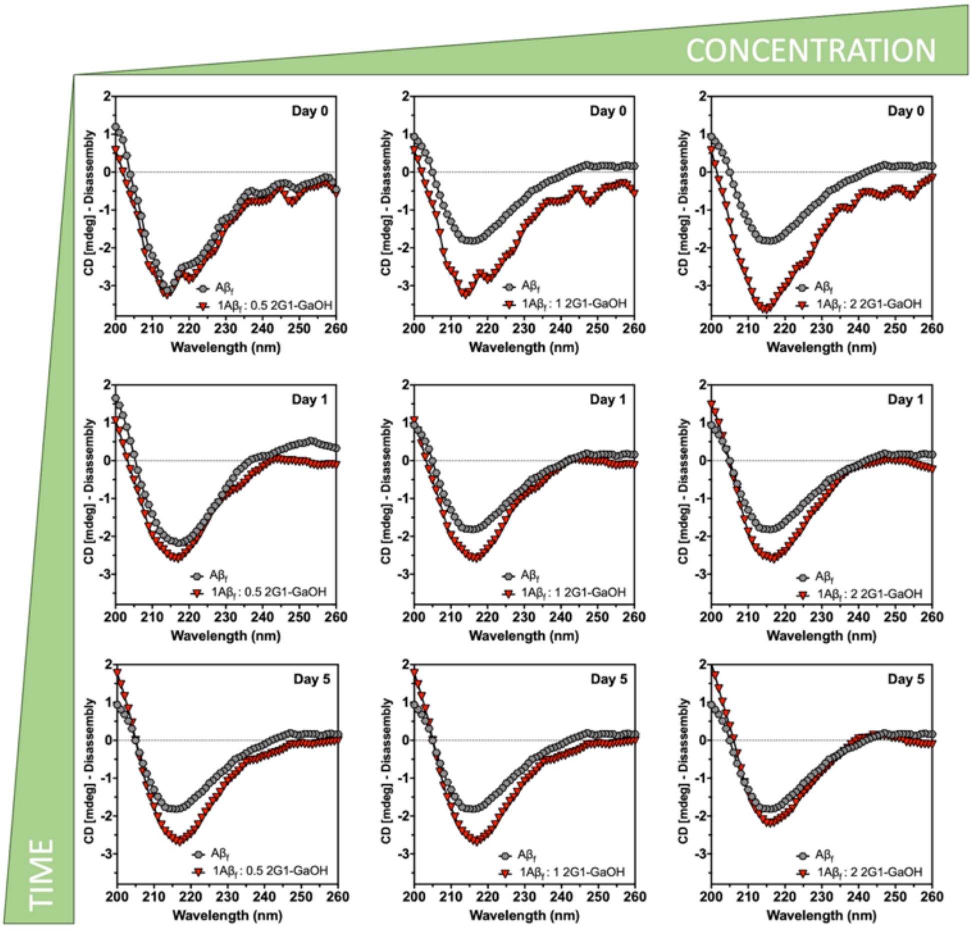

Figure S41. CD spectra of $A\beta_f$ recorded for 5 days in the presence of different molar ratios of 2G1-GaOH. In all the cases, incubation was made at room temperature, under constant agitation.

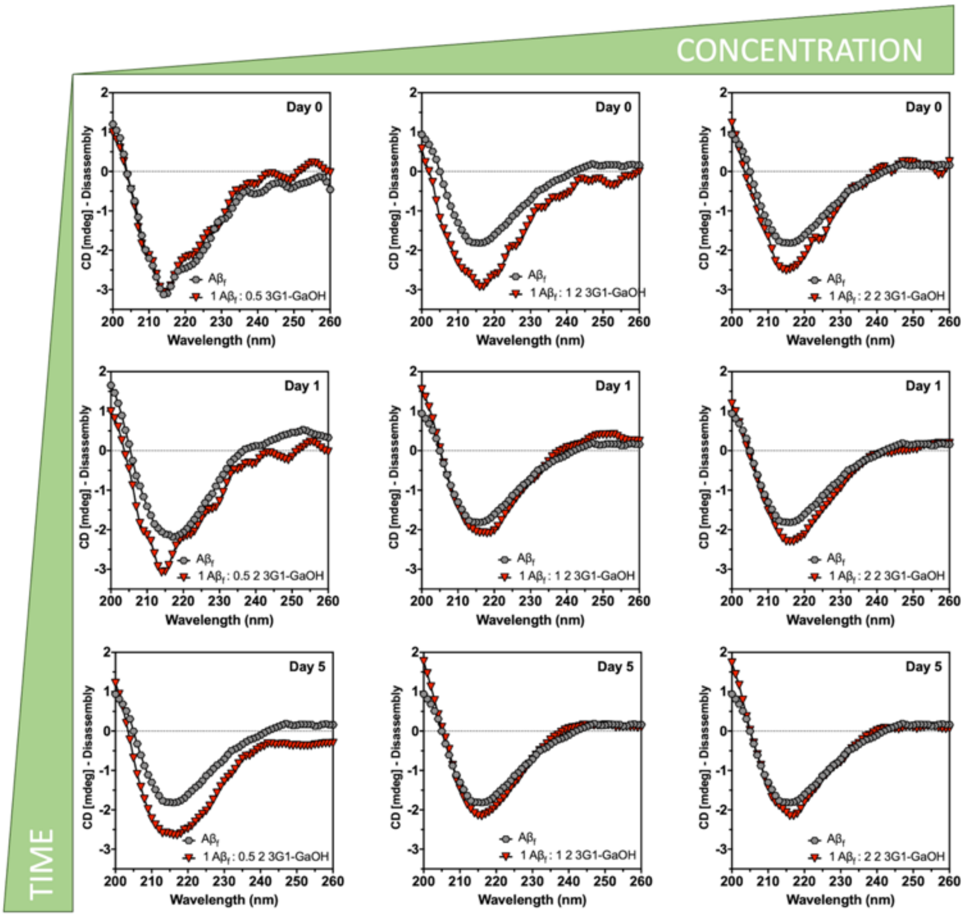

Figure S42. CD spectra of $A\beta_f$ recorded for 5 days in the presence of different molar ratios of 3G1-GaOH. In all the cases, incubation was made at room temperature, under constant agitation.

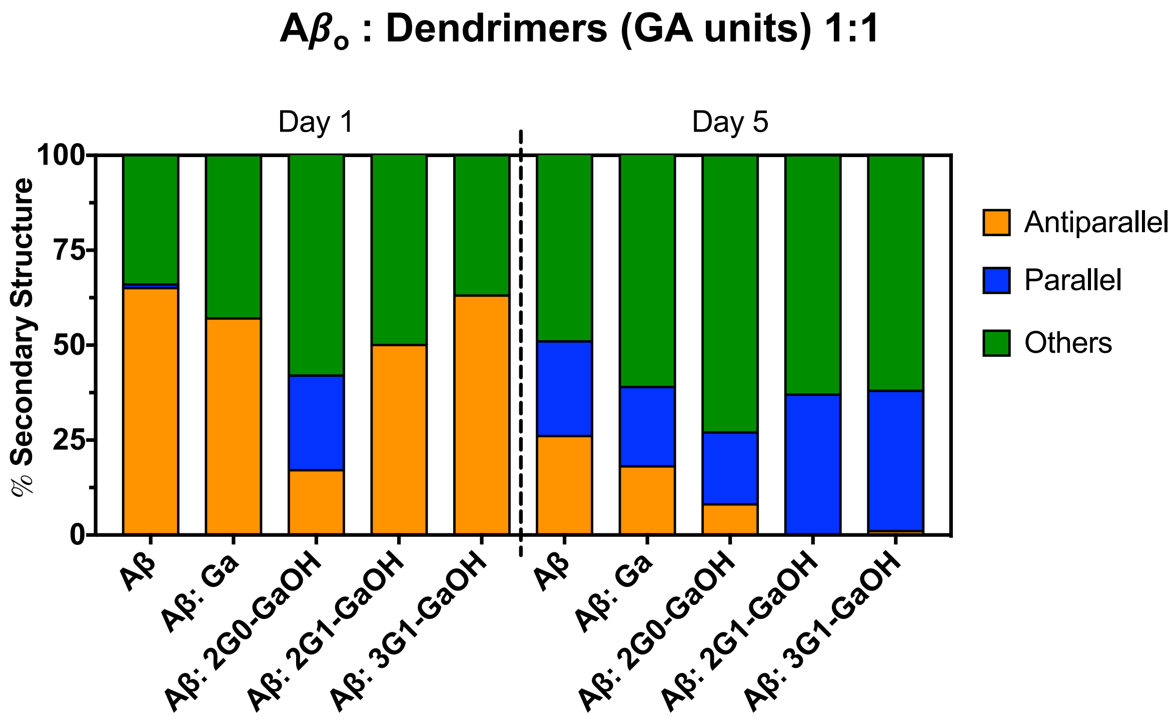

Figure S43. Loss of antiparallel β-sheets for the aggregation pathway ($A\beta_o$) followed by CD during 1 and 5 days. All experiments were done using an Aβ:Ga molar ratio of 1:1; [Aβ]=25µM; and under constant agitation at 37 °C. Error bars = SD; n = 3. CD data were fitted using BeStSel - RMSD: 1.0283; NRMSD: 0.04966.

Scanning transmission electron microscopy (STEM)

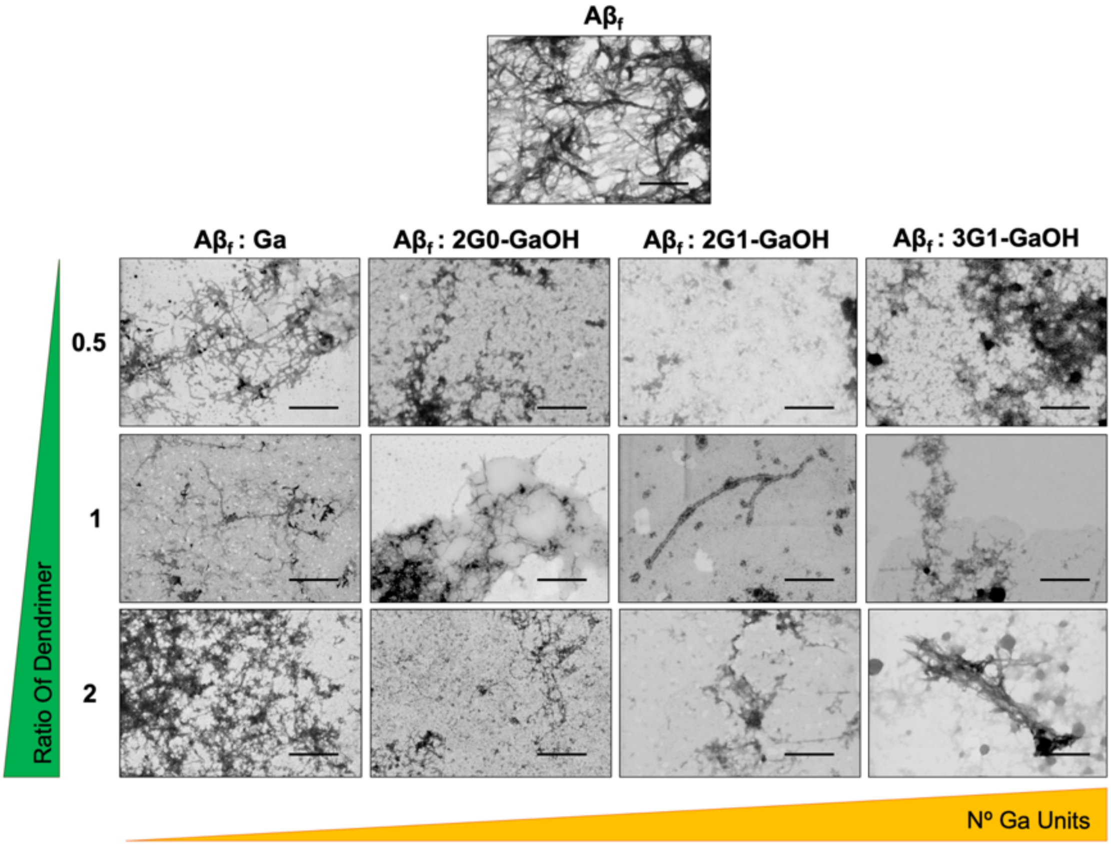

Figure S44. Representative STEM images of Aβ_f samples. Each dendrimer was added into an Aβ_f solution (under Aβ:dendrimer concentration ratios of 1:0.5, 1:1 and 1:2) and left to incubate for 24h under constant agitation. Both compounds directly altered Aβ_f morphological presentation. Scale bars = 500 nm.

Atomic Force Microscopy (AFM)

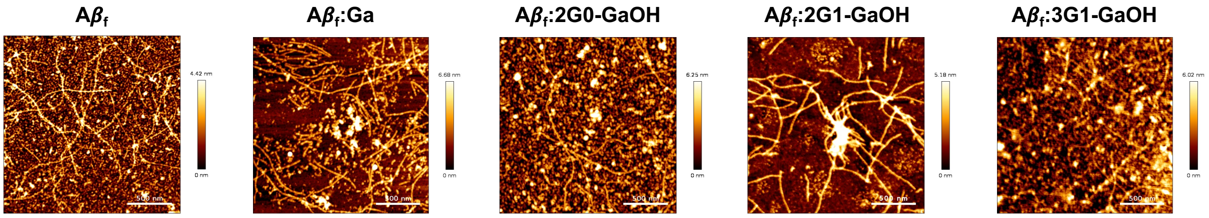

Figure S45. Representative AFM images of Aβ_f. Each dendrimer was added into an Aβ_f solution (Aβ:Ga molar ratio of 1:1) and left to incubate for 24h under constant agitation. The dendrimers with higher number of Ga units (i.e., 2G1-GaOH and 3G1-GaOH) were more effective than Ga to alter the Aβ_f presentation. Scale bars = 500 nm.

Western Blot (WB) analysis

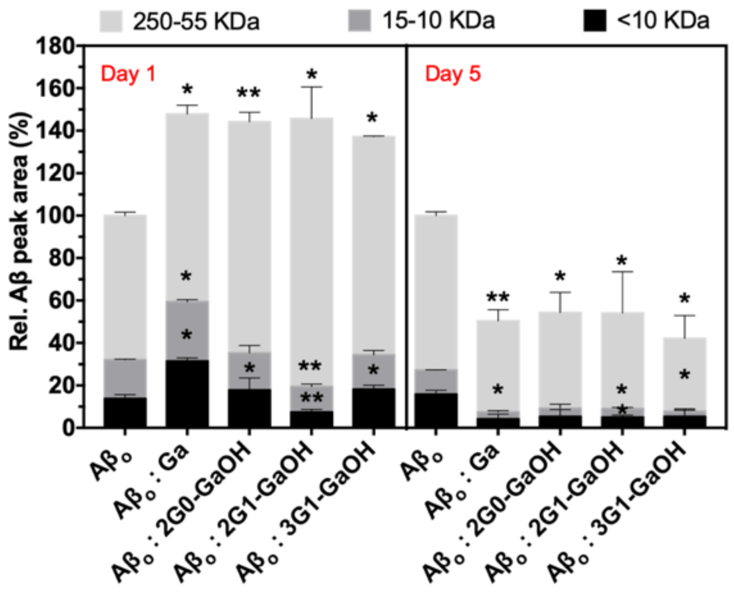

Figure S46. Relative densitometric bar graphs of $A\beta_o$ (25µM) assembly quantified by WB (using the antibody 6E10); experiments were executed at 37°C in PBS, during 1 and 5 days. Error bars = SD, * $p < 0.05$, ** $p < 0.01$ and *** $p < 0.001$ vs control 25µM $A\beta_o$; n=3.

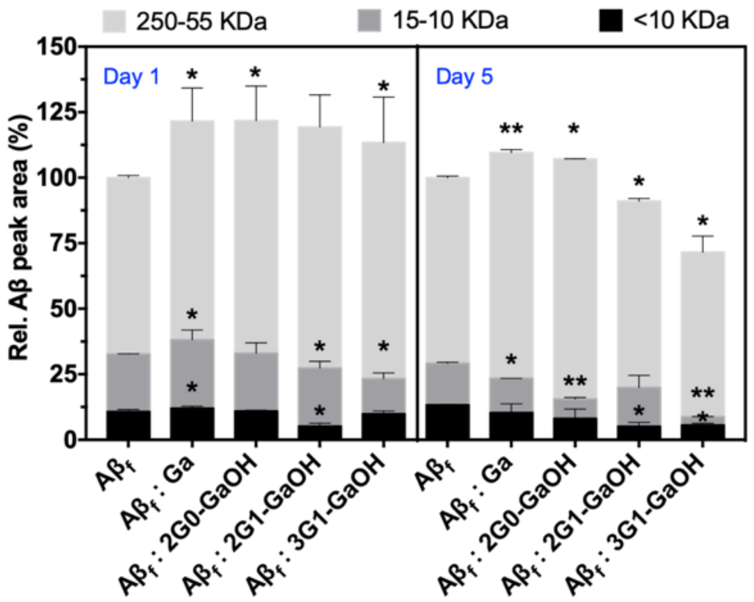

Figure S47. Relative densitometric bar graphs of $A\beta_f$ (25µM) disassembly quantified by WB (using the antibody 6E10); experiments were executed at 37°C in PBS, during 1 and 5 days. Error bars = SD, * $p < 0.05$, ** $p < 0.01$ and *** $p < 0.001$ vs control 25µM $A\beta_f$; n=3.

2 CELL STUDIES

Ability of the dendrimers to modulate the Aβ cytotoxicity

AlamarBlue® and Live/Dead assays

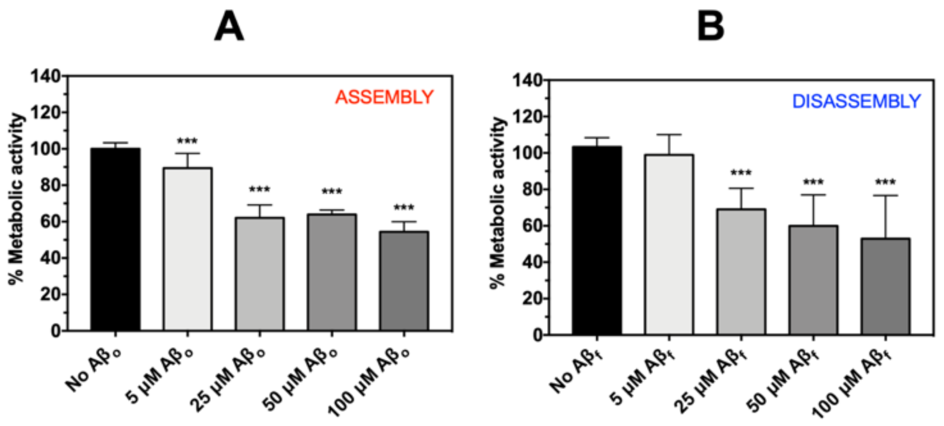

Figure S48. SH-SY5Y cell viability in the presence of (A) Aβ oligomers (Aβ$_o$, 25 µM) and (B) Aβ fibrils (Aβ$_f$, 25 µM) during 24h (using AlmarBlue® assay). Cells were incubated during 24h with different concentrations of Aβ. * $p < 0.05$, ** $p < 0.01$, *** $p < 0.001$ (vs control); n=3.

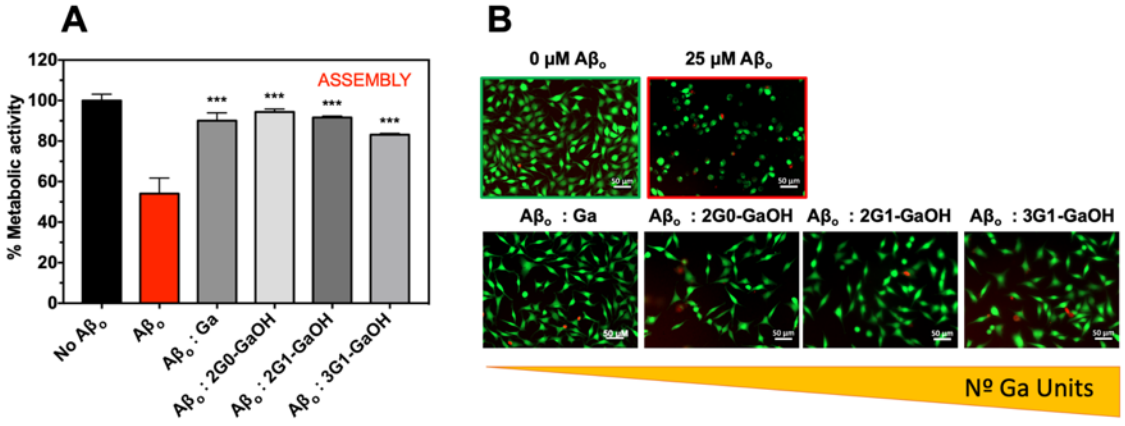

Figure S49. (A) Metabolic activity measured by AlamarBlue® assay of SH-SY5Y in the presence of a solution of Aβ$_o$ oligomeric species (25µM) during 24h, for a Aβ:dendrimers concentration ratio of 1:1. *** $p < 0.001$ (vs fibrillar Aβ); n=3; (B) Representative images of the Live/Dead assay. Scale bar = 50µm; n=3.

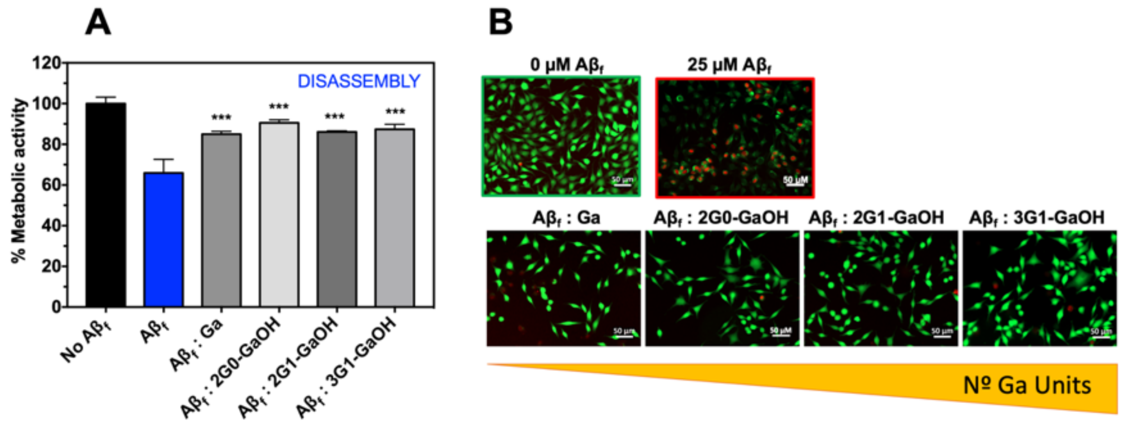

Figure S50. (A) Metabolic activity measured by AlamarBlue® assay of SH-SY5Y in the presence of a solution of Aβ_f pre-formed fibrils (25μM) during 24h, for a Aβ:dendrimers concentration ratio of 1:1. *** $p < 0.001$ (vs No Aβ); n=3. (B) Representative images of the Live/Dead assay. Scale bar = 50μm; n=3.

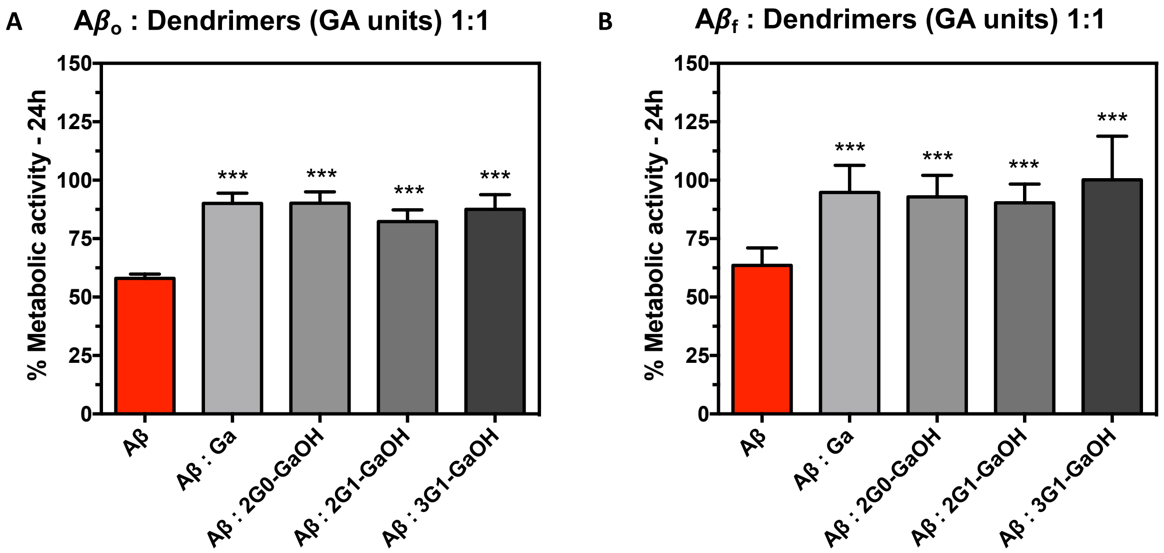

Figure S51. Metabolic activity measured by AlamarBlue® assay of SH-SY5Y in the presence of a solution of (A) Aβ_o and (B) Aβ_f pre-formed fibrils (25μM) during 24h. Experiments done at an Aβ:Ga molar ratio of 1:1. *** $p < 0.001$ (vs Aβ); n=3.

Quantification of the β-sheet content under cell culture determined by ThT assay

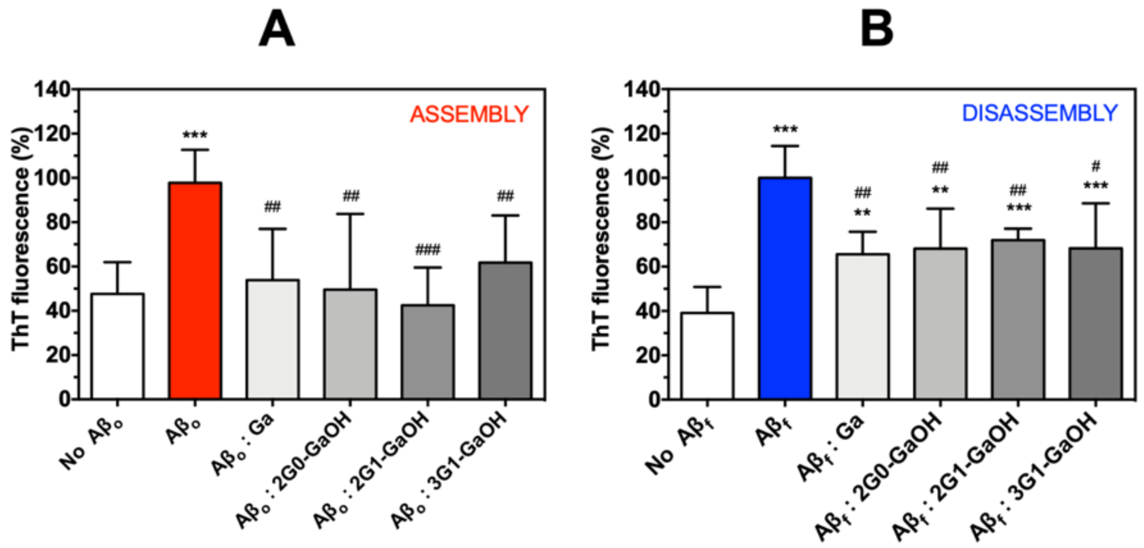

Figure S52. ThT fluorescence measurements in SH-SY5Y cell culture, after incubation with $A\beta_o$ (A) and $A\beta_f$ (B) species and dendrimers for a Aβ:dendrimers concentration ratio of 1:1, during 1 day of culture. Error bars = SD, $^{###}$ $p < 0.01$ (vs control 25μM Aβ); * $p < 0.05$, ** $p < 0.01$ and *** $p < 0.001$ vs No Aβ; n=3.

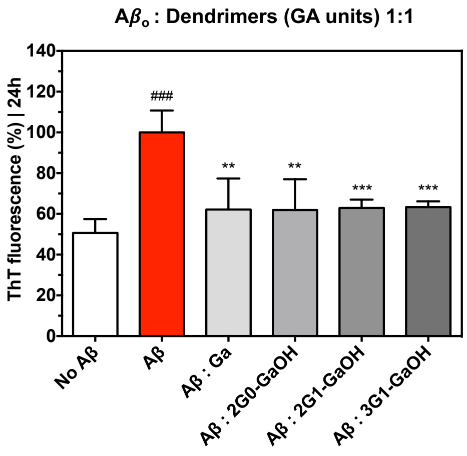

Figure S53. ThT fluorescence measurements in SH-SY5Y cell culture, after incubation with $A\beta_o$ species and dendrimers (Aβ:Ga molar ratio of 1:1), during 1 day of culture. Error bars = SD, $^{###}$ $p < 0.01$ (vs No Aβ); ** $p < 0.01$ and *** $p < 0.001$ vs control 25μM Aβ; n=3.

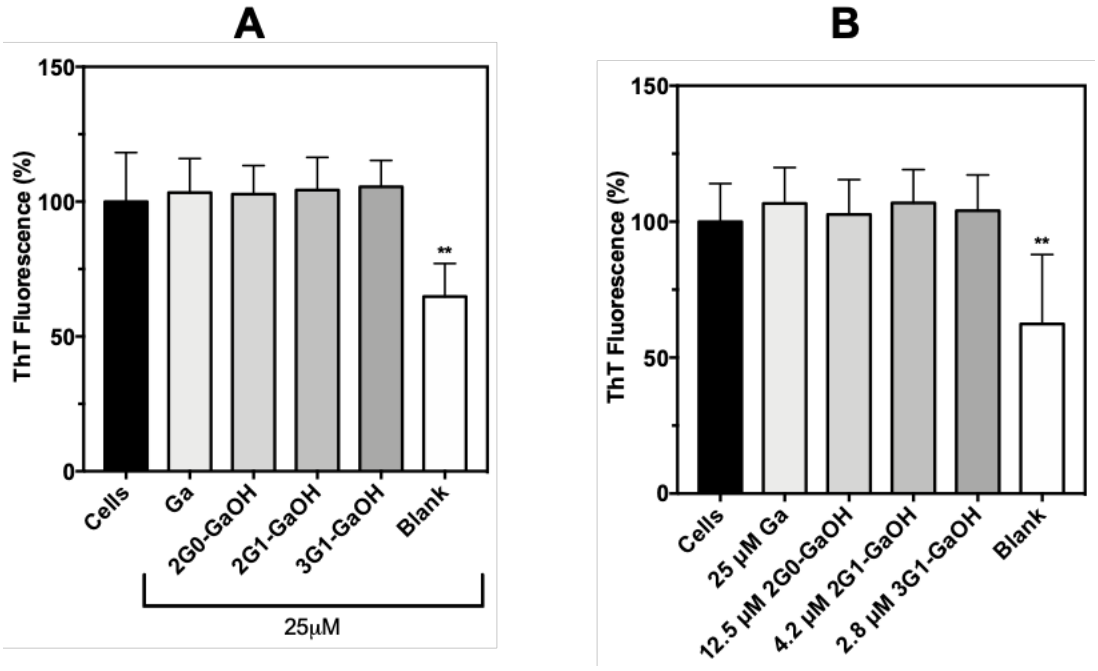

Figure S54. ThT fluorescence measurements in SH-SY5Y cell culture, after 1 day of incubation with A. solution of dendrimers at 25 μM concentration and B. solution of dendrimers at the concentrations used in the multivalency experiments (i.e., Aβ:Ga 1:1 molar ratio, per gallate). Error bars = SD, ** $p < 0.01$; n=3.

Protein expression

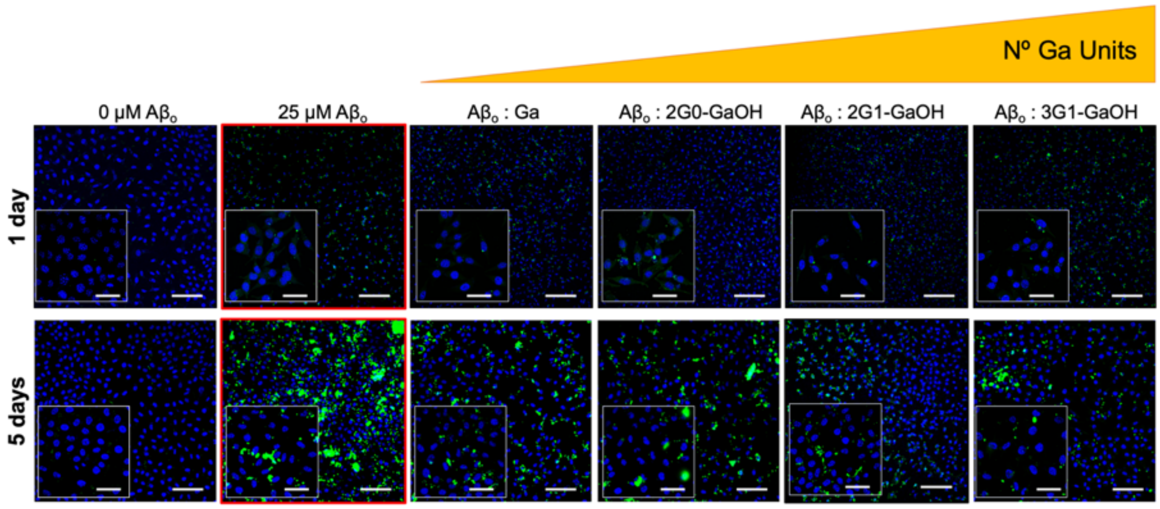

Figure S55. Immunofluorescence analysis of Aβ$_o$ species in the SH-SY5Y cell culture visualized by confocal microscopy (mAb 6E10, green) after incubation with dendrimers for a Aβ:dendrimer concentration ratio of 1:1, during 1 and 5 days (Aβ: green, cell nuclei: blue). Scale bar: 200μm (50μm for the insets).

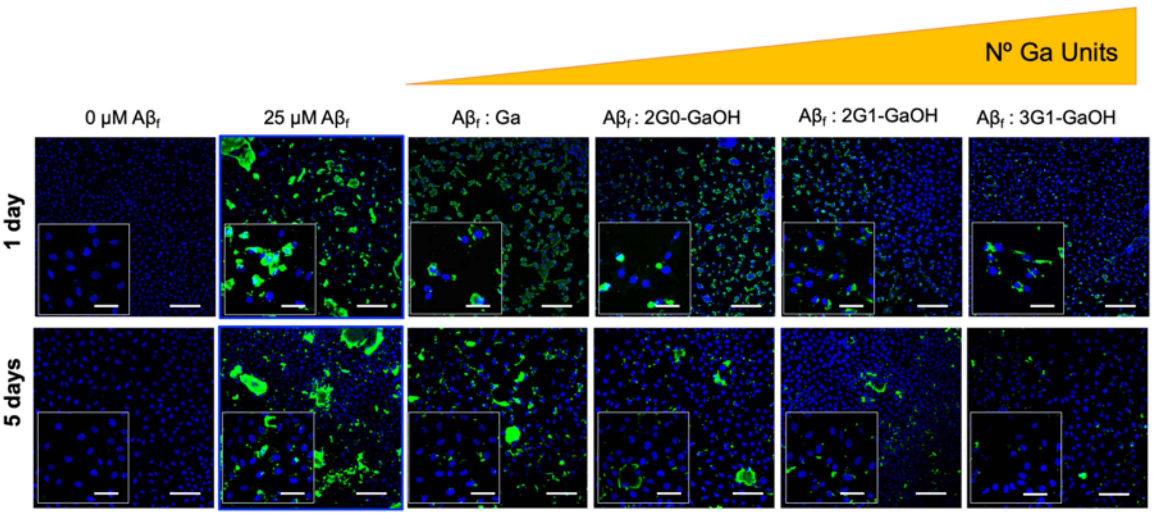

Figure S56. Immunofluorescence analysis of Aβ$_f$ species in the SH-SY5Y cell culture visualized by confocal microscopy (mAb 6E10, green) after incubation of dendrimers for a Aβ:dendrimers concentration ratio of 1:1, during 1 and 5 days (Aβ: green, cell nuclei: blue). Scale bar: 200 μm (50 μm for the insets).

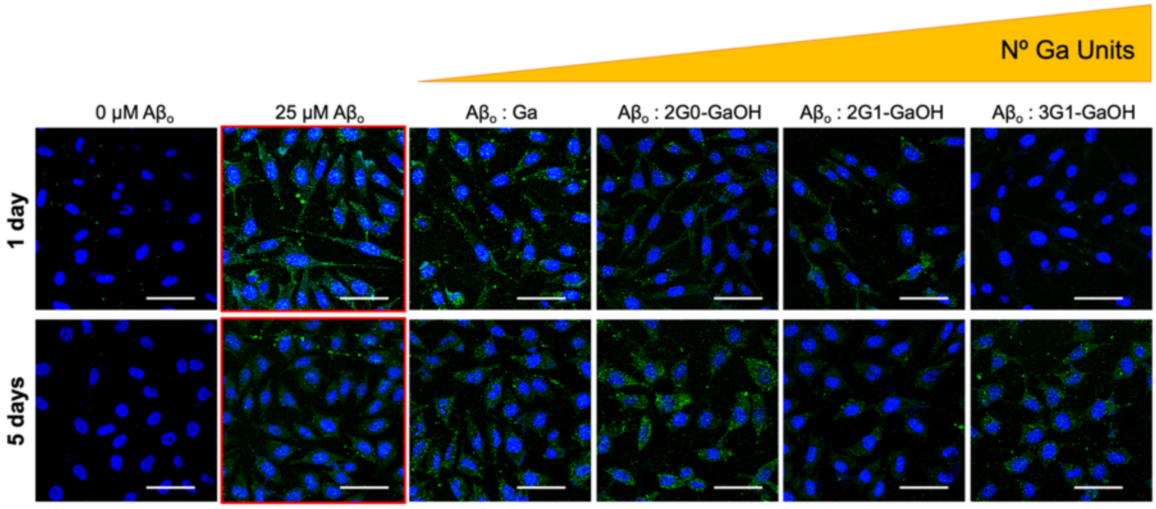

Figure S57. Immunofluorescence analysis of Aβₒ species in the SH-SY5Y cell culture visualized by confocal microscopy (A11 antibody, green) after incubation of dendrimers for a Aβ:dendrimers concentration ratio of 1:1, during 1 and 5 days (Aβ: green, cell nuclei: blue). A11 antibody specifically bind to oligomeric Aβ species. Scale bar: 50µm.

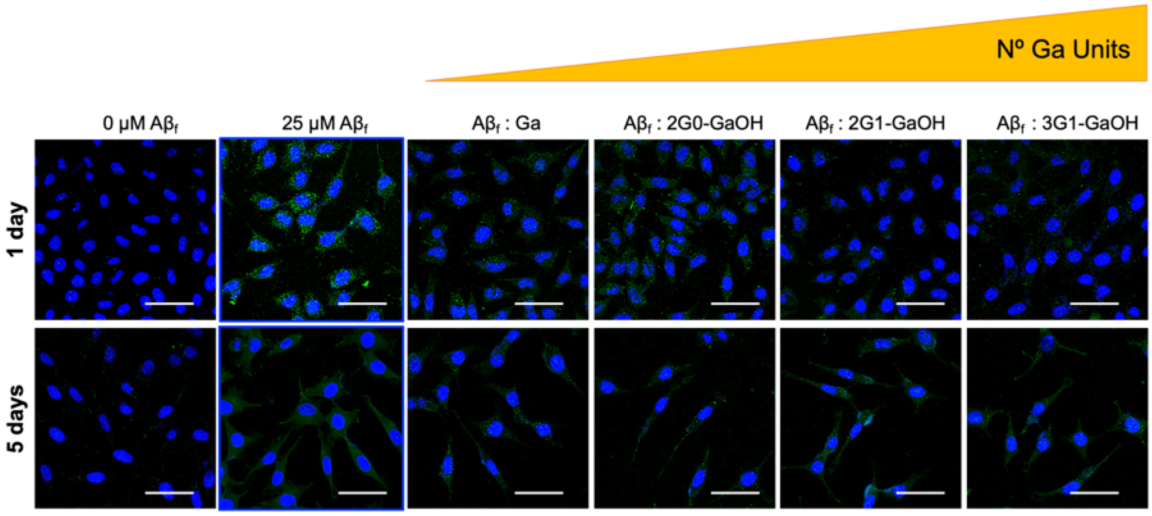

Figure S58. Immunofluorescence analysis of $A\beta_f$ species in the SH-SY5Y cell culture visualized by confocal microscopy (A11 antibody, green) after incubation of dendrimers to a 1:1 ratio, for 1 and 5 days (Aβ: green, cell nuclei: blue). A11 antibody specifically bind to oligomeric Aβ species. Scale bar: 50 µm.

Bio-AFM experiments

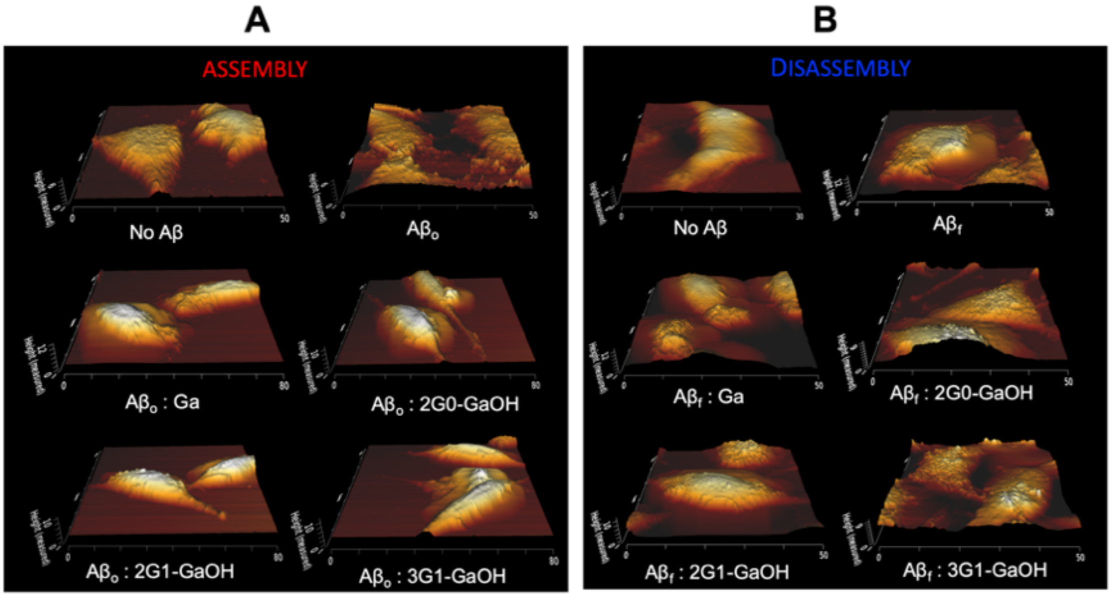

Figure S59. Representative AFM topographic images of SH-SY5Y cells cultured during 24h in the presence of (A) $A\beta_o$ (assembly) and (B) $A\beta_f$ (disassembly) and Ga, 2G0-GaOH, 2G1-GaOH and 3G1-GaOH using a Aβ:dendrimers ratio of 1:1.

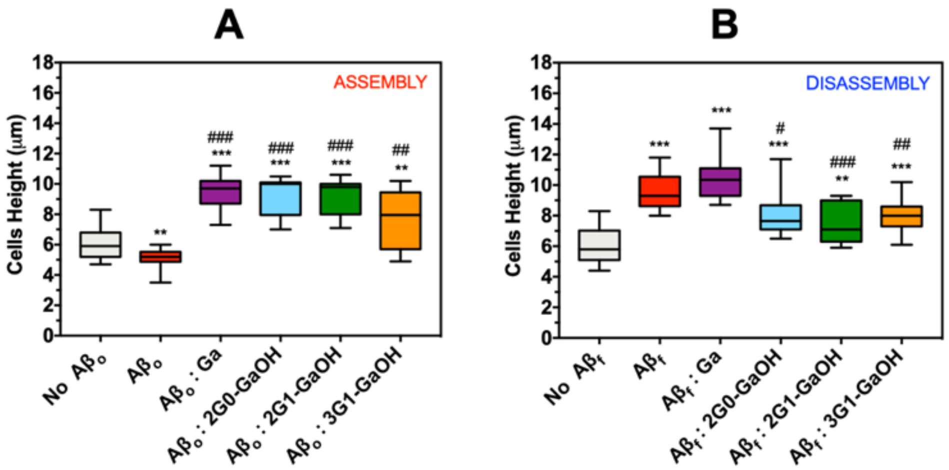

Figure S60. Statistical comparison of the cells height profile from the nanomechanical properties obtained by AFM topographic images of normal SH-SY5Y cells with 1:1 ratio of Aβ:dendrimers, under different conditions: (A) assembly pathway ($A\beta_o$) and (B) disassembly pathway ($A\beta_f$). All experiments were conducted after 24h of incubation with Aβ (25µM). Error bars = SD, $^{###}$ $p < 0.01$ (vs No Aβ); ** $p < 0.01$ and *** $p < 0.001$ vs control 25µM $A\beta_o/A\beta_f$; n=3.
